# Expulsion mechanism of the substrate-translocating subunit in ECF transporters

**DOI:** 10.1038/s41467-023-40266-1

**Published:** 2023-07-25

**Authors:** Chancievan Thangaratnarajah, Mark Nijland, Luís Borges-Araújo, Aike Jeucken, Jan Rheinberger, Siewert J. Marrink, Paulo C. T. Souza, Cristina Paulino, Dirk J. Slotboom

**Affiliations:** 1 Faculty of Science and Engineering, Groningen Biomolecular Sciences and Biotechnology, Membrane Enzymology Group, University of Groningen, 9747 AG Groningen, The Netherlands; 2 Faculty of Science and Engineering, Groningen Biomolecular Sciences and Biotechnology, Electron Microscopy Group, University of Groningen, 9747 AG Groningen, The Netherlands; 3 Molecular Microbiology and Structural Biochemistry, CNRS and University of Lyon, 69367 Lyon, France; 4 Biochemistry Center, University of Heidelberg, Im Neuenheimer Feld 328, 69120 Heidelberg, Germany; 5 Faculty of Science and Engineering, Groningen Biomolecular Sciences and Biotechnology, Molecular Dynamics Group, University of Groningen, 9747 AG Groningen, The Netherlands

**Keywords:** Cryoelectron microscopy, Membrane proteins

## Abstract

Energy-coupling factor (ECF)-type transporters mediate the uptake of micronutrients in many bacteria. They consist of a substrate-translocating subunit (S-component) and an ATP-hydrolysing motor (ECF module) Previous data indicate that the S-component topples within the membrane to alternately expose the binding site to either side of the membrane. In many ECF transporters, the substrate-free S-component can be expelled from the ECF module. Here we study this enigmatic expulsion step by cryogenic electron microscopy and reveal that ATP induces a concave-to-convex shape change of two long helices in the motor, thereby destroying the S-component’s docking site and allowing for its dissociation. We show that adaptation of the membrane morphology to the conformational state of the motor may favour expulsion of the substrate-free S-component when ATP is bound and docking of the substrate-loaded S-component after hydrolysis. Our work provides a picture of bilayer-assisted chemo-mechanical coupling in the transport cycle of ECF transporters.

## Abstract (web summary)

The final step in an ECF transporters transport cycle involves the expulsion of the membrane embedded substrate binding protein (the S-component) from the motor (the ECF module). Here the authors show how the motor uses ATP binding to load a molecular spring, and adjusts the shape of the membrane, to achieve this step.

## Introduction

Energy-coupling factor (ECF)-type transporters mediate the import of micronutrients, such as folate, cobalamin and pantothenate, in the majority of prokaryotes. They belong to the phylogenetically and mechanistically distinct type III clade of the ATP-binding cassette (ABC) transporter superfamily^1^, which was first described in the late 1970s^2^, with the molecular identity only uncovered in the late 2000s^3^. A tripartite ATP-hydrolysing motor (ECF module), composed of a membrane-embedded scaffold protein (EcfT) and two cytosolic nucleotide-binding proteins or ATPase subunits (often hetero-dimeric - EcfA and EcfA’), forms a functional core complex with a membrane-embedded substrate-translocating protein (S-component). Structural and biophysical analyses of ECF transporter complexes from three different organisms, with different substrate specificities, and from different laboratories revealed that the core complex and minimal functional unit exists in a 1:1:1:1 subunit stoichiometry^4–12^. Deviating subunit stoichiometries have been reported^13,14^ before the first structural insights became available^8,9^, which may indicate the existence of transient higher order complexes and have been previously discussed elsewhere^2^. In group I ECF transporters, the ECF module interacts with a dedicated S-component^3^, while in group II ECF transporters, S-components with different substrate specificities compete with each other for association with the ECF module on a single docking site^6,12^. In the latter group, S-components are hypothesized to dynamically associate with and dissociate from the ECF module during turnover, with both the motor and the S-component existing transiently as solitary units embedded in the lipid bilayer^4,12^. Structural information on ECF transporters is sparse. Only structures of the substrate-bound conformation of solitary S-components^4,15–19^, the isolated water-soluble ATPase subunits (EcfAA’)^12,14^, and the inward-facing post-substrate-release state of full ECF transporter complexes^4–10^, have been determined to date. Based on these fragmented structural insights, a model for the transport mechanism has been proposed in which a substrate-free solitary S-component with an outward-facing binding site topples within the membrane upon substrate acquisition, associates with the ECF module, and exposes the binding site to the cytosol^4,12^, thereby moving the substrate across the membrane. Coarse-grained molecular dynamics (MD) simulations have indicated that membrane deformations around the ECF module may provide a favourable local environment to facilitate toppling of the S-component^20,21^. A cryogenic electron microscopy (cryo-EM) structure of the group II folate-specific ECF transporter from *Lactobacillus delbrueckii* (ECF-FolT2) in lipid nanodiscs corroborated that the transporter indeed induces local deformations to the membrane^5^, which is likely further modulated in different conformations. Using the isolated soluble ATPase subunits from *Thermotoga maritima*^12,14^, it has been demonstrated that ATP binding switches the dimer of ATPase subunits from an open conformation, with the two subunits partially separated, to a closed tightly associated dimer. It has been hypothesized that in the context of the full complex, this movement would lead to expulsion of the S-component into the lipid bilayer environment, and subsequent reorientation of the S-component from the toppled inward-facing to the straight outward-facing conformation for renewed substrate binding and transport^4,12^. However, the limited structural data available precludes mechanistic understanding of this highly unusual expulsion step, which is central to the mechanism of transport in group II ECF transporters.

Here, our work uncovers a long sought-after key step delineating how Mg-ATP binding expels the S-component into the membrane and leads to resetting of group II ECF transporters for a new transport cycle.

## Results

### Nucleotide binding fails to induce conformational changes in nanodiscs

**Fig. 1:**
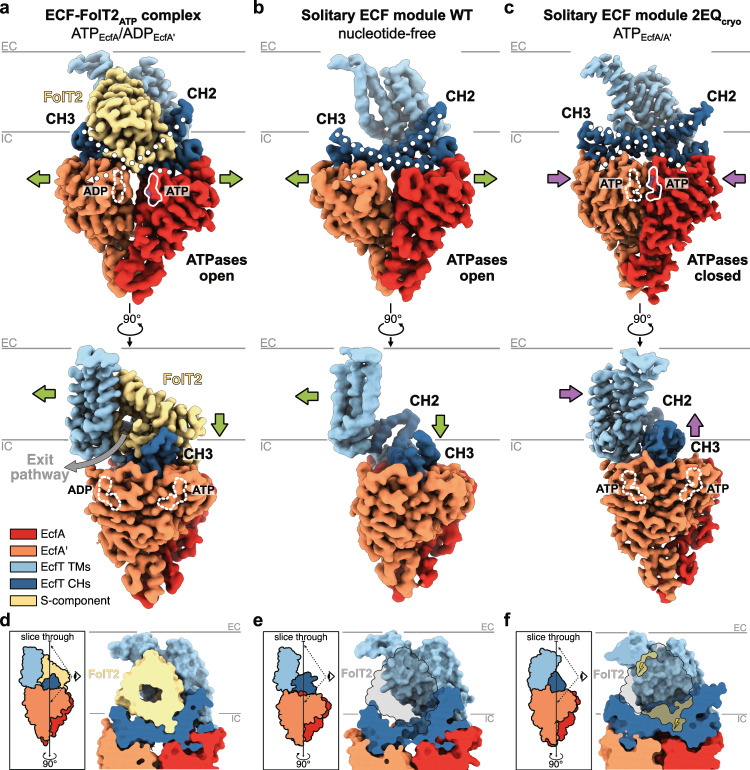
Conformational states of ECF transporters. Cryo-EM maps of the nucleotide-bound ECF-FolT2_ATP_ complex (**a**), the nucleotide-free wild-type solitary ECF module (**b**), and the ATP-bound mutant solitary ECF module 2EQ_cryo_ (**c**), all in lipid nanodiscs, viewed from the membrane plane onto the docking site of FolT2 (top) and rotated by 90° onto the site of the cytoplasmic substrate exit pathway (grey arrow) for folate from the cavity in FolT2. The shape of the coupling helices (CH2 and CH3) is indicated by white dotted lines with the terminal triangles indicating the conserved X-Arg-X anchor motifs. Bound nucleotides are indicated by white contours (solid lines - located towards the front, dashed lines - towards the back) and labels. The green arrows in (**a**) and (**b**) indicate the direction in which the structural elements have moved relative to (**c**). Similarly, the purple arrows indicate the opposite movement. Cross sections of the ECF-FolT2_ATP_ complex (**d**), the wild-type solitary ECF module (**e**), and the mutant solitary ECF module 2EQ_cryo_ (**f**) revealing the shape of the interaction surface of the coupling helices and the S-component. Models are shown in surface representation. The schematic representations in the boxes indicate the position of the slice through and the zoomed in region of the cross section. An outline of FolT2 is shown highlighting a good fit without clashes with the ECF module in the ATPase open conformation (**e**) or clashes with the ECF module in the ATPase closed conformation (**f**). **f** The surfaces of the clashes are shaded in yellow and are indicated with a lightning symbol. **a**–**f** The approximate boundaries (grey lines) of the membrane and the extracellular (EC) and intracellular (IC) sides are indicated. The individual subunits/regions are coloured as follows: EcfA in red, EcfA’ in orange, EcfT coupling helices (CHs) in dark blue, EcfT transmembrane domain (TMs) in light blue, and the S-component FolT2 in yellow.

Our previous work on ECF-FolT2 in lipid nanodiscs provided a starting point for examining asymmetric ~115 kDa ECF transporters at high-resolution by cryo-EM^5,22^. We aimed to obtain structures of the complex under turnover conditions, a strategy that was used previously for other transporters^23–25^. Accordingly, we incubated ECF-FolT2 with Mg-ATP and folate prior to sample vitrification. Although ATP hydrolysis takes place in this condition^4,5^, we only found a single conformation of the complex at 3.2 Å resolution (Fig. 1a and Supplementary Figs. 1 and 2), which strongly resembles our previous structure determined in the absence of nucleotides and folate (PDB: 7NNU, rmsd: ~0.7 Å)^5^. The S-component for folate (FolT2) remains docked to the EcfT subunit in the substrate-free inward-facing toppled state (Fig. 1a). The ATPase dimer adopts the open conformation, albeit with bound nucleotides (Supplementary Fig. 3a). The nucleotides interact only with motifs from a single ATPase subunit and are separated from the signature motif (LSGGQ) located in the other ATPase (Supplementary Fig. 3a). Using a similar workflow, we incubated the complex with the non-hydrolysable ATP analogue AMP-PNP (ECF-FolT2_AMP-PNP_) instead of Mg-ATP, a strategy that was used to obtain a crystal structure of the isolated water-soluble ECF ATPase dimer from *T. maritima* in a closed conformation^12^. Our 3.6 Å resolution cryo-EM reconstruction (Supplementary Figs. 4–6), again revealed the complex in the same overall conformation, with AMP-PNP bound to both nucleotide-binding sites, but not engaged with the signature motif (Supplementary Fig. 3b). As such, ECF-FolT2 adopts the same conformational state irrespective of the presence or absence of either ATP or AMP-PNP. Apparently, the isolated ATPase subunits in aqueous solution^12,14^ behave differently from the full ECF-FolT2 complex in lipid nanodisc environment.

Notably, the nucleotide occupancy in the nucleotide-binding sites of ECF-FolT2_ATP_ is asymmetric with ATP and ADP bound to the EcfA and EcfA’ subunits, respectively (Supplementary Fig. 3a), which has been also observed in other ABC transporters^23^. ADP is bound exclusively by residues from the EcfA’ subunit (Supplementary Fig. 3a), and its presence suggests that ATP hydrolysis had occurred. In contrast, ATP remained present in the EcfA subunit, where the adenine moiety of ATP is sandwiched through π-interactions between Phe13 in the EcfA subunit and Arg174 in the carboxy-terminal end of the S-component FolT2 (Supplementary Fig. 3a). This interaction was also observed with AMP-PNP in ECF-FolT_AMP-PNP_ (Supplementary Fig. 3b). The presence of positively charged residues at the carboxy-terminal end of different S-components is a conserved feature^5^. We hypothesise that the direct interaction between the S-component and the nucleotide could affect the hydrolysis of ATP. Possibly, ATP at this site can only be hydrolysed once the S-component has changed conformation, which apparently does not happen in the nanodisc environment.

For the ABC transporter MsbA^26^, it has been shown that conformational freedom is restricted by the nanodisc belt. The lipid nanodisc assembly may lead to a similar constrain in ECF-FolT2, hindering conformational rearrangements required for the release of the S-component and for ATP hydrolysis in both sites. To assess this possibility, we tried to obtain samples for cryo-EM with ECF-FolT2 in DDM micelles instead of lipid nanodiscs. While structures of ECF-FolT2 in DDM micelles have been determined previously in the absence of Mg-ATP and folate, the protein complex rapidly aggregated under turnover conditions prior to sample vitrification, preventing structure determination. Nonetheless, this observation indicates that conformational changes take place under turnover conditions in detergent solution. The aggregation possibly occurred due to the expulsion of the S-component and the associated fission of the micelle leading to the exposure of hydrophobic surfaces to the aqueous environment.

### In vitro complex assembly and structure of the solitary ECF module

**Fig. 2:**
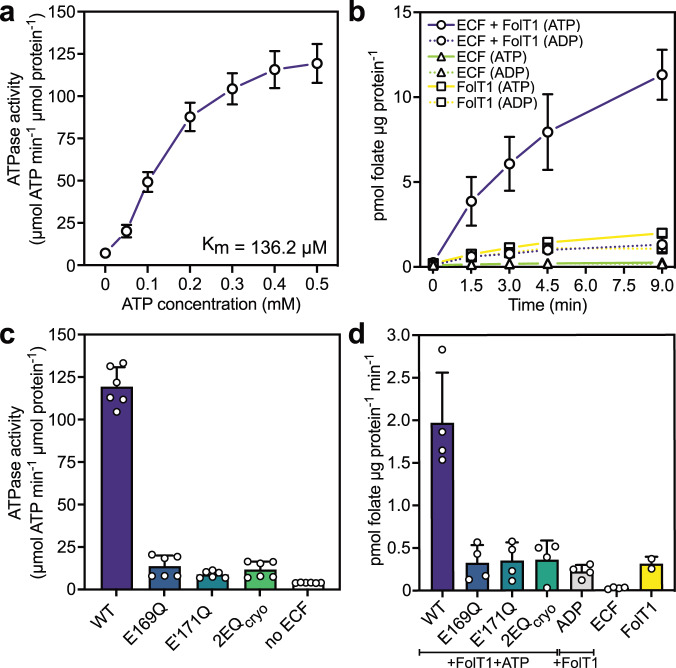
Functional characterisation of the purified ECF module. **a** ATPase activity of the solitary ECF module embedded in lipid nanodiscs. The Vmax and K_M_ values for ATP hydrolysis are 132.6 µmol min^−1^ µmol^−1^ and 136.2 µM, respectively. **b** Transport of folate into liposomes with co-reconstituted purified solitary ECF module and solitary FolT1 (circles), or reconstituted with only solitary ECF module alone (triangles) or solitary FolT1 alone (squares). Proteoliposomes were either loaded with 5 mM Mg-ATP (solid lines) or 5 mM Mg-ADP (dashed lines). **c** Comparison of the ATPase activity of nanodisc-embedded solitary ECF modules. Wild-type (purple), E169Q mutant (blue), E’171Q mutant (teal), 2EQ_cryo_ mutant (green) or without protein (white). Experiments were performed in presence of 0.5 mM Mg-ATP. **d** Comparison of folate transport into liposomes co-reconstituted solitary ECF modules and FolT1. Wild-type (purple), E169Q mutant (blue), E’171Q mutant (teal), 2EQ_cryo_ mutant (green), the wild-type solitary ECF module alone (white), and FolT1 alone (yellow) in the presence of 5 mM Mg-ATP, and with the wild-type solitary ECF module + FolT1 in the presence of 5 mM Mg-ADP (grey). **a**–**d** Data were obtained from two biological repeats, each containing three technical repeats (ATPase assays) or two technical repeats (transport assays), with the exception of the transport assays for FolT1, which is data from a single experiment with two technical repeats. Data are presented as the mean with the error bars indicating the sample standard deviation. Source data are provided as a Source Data file.

Next, we shifted our attention to the solitary ECF module of ECF-FolT2, which according to in vivo^27,28^ and in vitro^6^ studies must represent, at least transiently, an existing functional state in cells. The ~92 kDa solitary ECF module itself represents a mechanistically important state, as eight different S-components (FolT1, FolT2, CbrT, PanT, PdxU1, PdxU2, RibU and BioY) can compete for dynamic interaction with it during turnover^3,5,6^. For the group I cobalt ECF transporter^29^ and the group II riboflavin ECF transporter^14^, it has been shown that a solitary ECF module in the absence of a co-expressed S-component can be purified as a stable complex. We overexpressed and purified the solitary ECF module from the *L. delbrueckii* complex in the absence of a co-expressed S-component (Supplementary Fig. 7a). To exclude the possibility that the ECF module misfolds into an inactive state, we first reconstituted the solitary ECF module into lipid nanodiscs (Supplementary Fig. 7b) and showed that the ATPase activity is retained in the absence of an S-component (Fig. 2a). We then co-reconstituted the solitary ECF module into liposomes with the separately purified solitary folate-specific S-component FolT1 (Supplementary Fig. 7a)^4^. Uptake of folate into the proteoliposome lumen only occurred in the presence of lumenal Mg-ATP and a co-reconstituted solitary S-component FolT1 (Fig. 2b). Together, these experiments demonstrate that the solitary ECF module retains its integrity upon purification, and importantly can re-associate with a solitary S-component to form a functional transporter complex, excluding the possibility that the complex had collapsed in an artefactual, inactive state and reinforcing the notion that a solitary ECF module can exist in cells.

We then obtained a cryo-EM reconstruction of the nucleotide-free solitary ECF module in lipid nanodiscs at 3.8 Å resolution that shows a vacant docking site for the S-component (Fig. 1b and Supplementary Figs. 8 and 9). The ATPase dimer adopts an open conformation (Fig. 1b), and the structure of the module is very similar to the conformation observed in full ECF transporter complexes presented above (rmsd: 1.8 Å) (Fig. 1a), as well as to the ECF modules of previously determined structures of the folate-, pantothenate- and cobalamin-specific ECF transporters from *L. delbrueckii* (rmsd: 1.6–1.8 Å)^4–7^. The main difference is that transmembrane helix 3 and the regions close to the extracellular side of the EcfT subunit in the solitary ECF module are less well resolved (Supplementary Fig. 9d). We attribute this to a higher degree of flexibility caused by the absence of any stabilising effect typically exerted by a bound S-component. This flexibility agrees with the capacity for the EcfT subunit to accommodate different S-components^4–7^. Notably, in the solitary ECF module, the docking site for the S-component remained structurally intact and unaltered compared to the docking site in the ECF-FolT2 complex. Yet, in contrast to the full complex, it is accessible to the lipid nanodisc environment (Supplementary Fig. 10a). Moreover, the docking site is hydrophobic (Supplementary Fig. 10b), and, therefore, compatible to interact with the surrounding environment of the lipid nanodisc. Such membrane-accessible interaction sites have been also observed in structures of the endoplasmic reticulum membrane protein complex (EMC)^30–34^. In the space between the ATPase dimer, we found an extra protein-resembling density (Supplementary Fig. 10a), which we attributed to the MSP2N2 belt protein. The location of this density coincides with the C-terminal part of transmembrane helix 6 of the S-component FolT2 in the full ECF transporter complex (Supplementary Fig. 10a). To exclude that the conformation of the solitary ECF module is impacted by the belt protein, we also obtained a reconstruction of the wild-type solitary ECF module in DDM micelles at 4.3 Å resolution (Supplementary Figs. 10c and 11). The conformation of the solitary ECF module is virtually identical, but without the extra protein-resembling density, excluding a nanodisc-induced artefactual conformation.

### Structure of the solitary ECF module in the ATP-bound closed state

Because the conformations of the ATPases dimers obtained in all structures so far were virtually identical, we reasoned that alternative conformations of the ATPase dimer in the ECF module (for instance, a closed conformation) may be short-lived, and difficult to investigate structurally. We, therefore, opted for a mutagenesis approach, rather than simply adding Mg-ATP, to increase the probability of capturing alternative conformations under ATP hydrolysing conditions. Similar mutagenesis approaches have often facilitated the conformational trapping of short-lived states, such as demonstrated by ATP-bound conformations of various canonical ABC transporters^23, 35–37^. We substituted the catalytic glutamates in the Walker B motif in both ATPase subunits, which are required for ATP hydrolysis, to glutamine. We first tested the effect of the mutations on transport in vivo, by using an *E. coli* strain that depends on a functional ECF transporter to take up the substrate cobalamin (ECF-CbrT from *L. delbrueckii*). The assay revealed that expression of each of the single mutants (EcfA E169Q and EcfA’ E’171Q), and of the double mutant led to a phenotype with severe growth defects in conditions where uptake of cobalamin was essential (Supplementary Fig 12), confirming the importance of the glutamates. We then purified the mutant ECF modules (Supplementary Fig. 7a) and assessed their function in vitro. Neither of the mutant motors exhibited ATP hydrolysis activity above background in lipid nanodiscs (Fig. 2c and Supplementary Fig. 7b), nor were they able to transport folate when co-reconstituted with FolT1 into liposomes (Fig. 2d). While the purified double mutant (referred to as 2EQ_cryo_) cannot hydrolyse ATP (Fig. 2c), it is still expected to bind nucleotides, which prompted us to use it for cryo-EM analysis. We obtained a reconstruction of the mutant solitary ECF module 2EQ_cryo_ in lipid nanodiscs at 2.6 Å resolution (Fig. 1c and Supplementary Figs. 13 and 14), which shows a markedly distinct conformation from any structure reported so far. In both ATPase subunits, Mg-ATP is now bound and engaged with the signature motif (Supplementary Fig. 3c). Compared to the open conformation, the ATPase subunits have moved by ~4 Å towards each other to adopt a closed conformation (Fig. 1c and Supplementary Fig. 15). Strikingly, this relatively small movement induced a much larger change in the EcfT subunit (Supplementary Fig. 15a, b and Supplementary Movie 1). Because the two long coupling helices (CH2 and CH3) of the EcfT subunit are anchored into the grooves of the EcfA and EcfA’ subunits, respectively, with conserved X-Arg-X motifs at their C-terminal ends (Supplementary Fig. 15c)^14,38^, the closing of the ATPase subunits into a tight dimer forces these C-terminal ends to move towards each other (Supplementary Fig. 15c). This movement thrusts the coupling helices from the membrane interface into the lipid environment by ~9 Å (Supplementary Fig. 15a), changing their shape from concave to convex (Fig. 1c). The convex conformation of the coupling helices adopted by the solitary ECF module upon Mg-ATP binding becomes incompatible with the shape of the S-component (Fig. 1f). In this state it can no longer provide a fitting platform for the docking of a toppled S-component, as found in the open and concave conformation (Fig. 1d, e). Concomitantly, the transmembrane regions of the EcfT subunit that are connected to the coupling helices, undergo a ~13.5 Å rigid body translation and a ~25 Å rotation towards the coupling helices within the lipid nanodisc, thereby moving into the space where S-components dock. These rearrangements make the module more compact than in the full complexes or the wild-type solitary ECF module (Fig. 1c and Supplementary Fig. 15b). Together, the conformational changes observed in our structure upon Mg-ATP binding provide an explanation on how S-components are expelled from the ECF module.

### Conformation-specific membrane perturbations

**Fig. 3:**
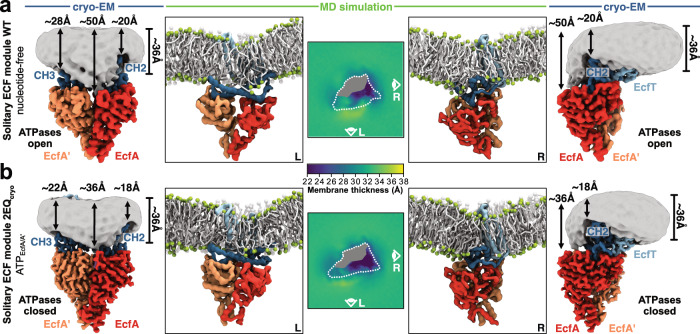
Conformation-specific membrane deformations mediated by the solitary ECF module. **a** Cryo-EM map of the wild-type solitary ECF module with open ATPases embedded in a lipid nanodisc viewed as in Fig. 1a–c from the membrane plane onto the docking site of FolT2 (left panel) or rotated by 90° (right panel). **b** Cryo-EM map of the mutant solitary ECF module 2EQ_cryo_ with closed ATPases embedded in a lipid nanodisc from the same viewpoints as in **a**. The approximate nanodisc thickness is indicated with black arrows in various regions determined using Chimera X^68^. The nanodisc densities (grey) were obtained from an unsharpened map lowpass filtered to 6 Å and contoured at 6.5 σ. Coarse-grained MD snapshots of both wild-type (**a**) and mutant (**b**) solitary ECF modules inserted in a bacterial model membrane (POPE: POPG: cardiolipin) from the same viewpoints as the cryo-EM maps (second panels from left and right). Lipid tails are coloured in white and lipid phosphodiester beads in green. Middle panels show membrane thickness maps around both wild-type (**a**) and mutant (**b**) solitary ECF modules. Outline represents the boundaries of the EcfT subunit with the region of transmembrane domain filled in grey. For each leaflet, lipid glycerol beads were mapped onto a *xy* grid, and the average grid cell lipid position determined. Membrane thickness was defined as the average z-distance between the corresponding pair of top and bottom leaflet grid cells. The individual subunits/regions are coloured as in Fig. 1.

The cryo-EM density profiles of the lipid nanodiscs surrounding the proteins vary extensively in thickness in the different reconstructions. This variation is especially pronounced around the docking site on the EcfT subunit for the S-component (Fig. 3a, b and Supplementary Fig. 10e), hinting at a role of conformational-specific local membrane deformations in the transport mechanism. In the presence of a bound S-component to the ECF module, the lipid nanodisc density is particularly thin (only ~23 Å) at the exposed face of the S-component (Supplementary Fig. 10e)^5^. This local thinning is needed to prevent exposure of the positively charged cytoplasmic base of the toppled S-component to the hydrophobic lipid environment (Supplementary Fig. 10f)^5^. In the absence of an S-component, the nanodisc shape depends strongly on the conformation of the ATPase dimer. In the ATPase open conformation, the lipid nanodisc has a dramatically increased thickness around the empty docking site (Fig. 3a). The observed thickness of ~50 Å in this area is needed to avoid the exposure of the hydrophobic docking site to the aqueous environment. Notably, the deformation of the lipid nanodisc aligns with the membrane-facing hydrophobic surface of the coupling helices (Supplementary Fig. 10a, b). The movement of the coupling helices towards the membrane plane upon Mg-ATP binding (Fig. 1c and Supplementary Fig. 15a), shifts the hydrophobic surface boundary, thereby reducing the thickness of the nanodisc density to ~36 Å in this region (Fig. 3b and Supplementary Fig. 10d, g). The thinner bilayer in the closed ATPase conformation matches the size of the leaving solitary S-component better than the thick belt around the open state, likely providing a more suitable environment to facilitate its dissociation.

As long-range membrane deformations cannot be observed in these reconstructions due to the limited size of the bilayer patch in the nanodisc assemblies, we conducted coarse-grained MD simulations of the solitary ECF module embedded into a bacterial model membrane. The simulations revealed that the solitary ECF module in both conformations was tilted relative to the membrane plane (Fig. 3a, b). Additionally, not only were the experimentally observed membrane thickness variations reproduced in the simulation (Fig. 3), also a strong negative curvature was revealed in the bilayer surrounding the docking site in both conformations (Supplementary Fig. 16a, b). These results are also consistent with previous atomistic simulations, which were performed with the ATPase open conformation only^20^. Together, the tilting of the module and the bending of the membrane lead to differences in the relative directions of the bilayer on either side of the module, which likely assist the S-component in the association with or expulsion from the ECF module during the transport cycle. Finally, we performed coarse-grained MD simulations of the full ECF transporter complex to obtain insight in the initial steps leading to the expulsion of the S-component from the ECF module. We simulated ECF modules in ATPase open and closed conformations, onto each of which we docked the S-component (Supplementary Fig. 16c, d). In the open conformation, the S-component expectedly remained stably positioned at a tilt-angle of ~50° with the membrane normal (Supplementary Fig. 16c, e). But in the closed conformation, the S-component started to rotate adopting a smaller tilt angle of ~40° (Supplementary Fig. 16d, e). This movement likely represents the first step in the reorientation of the S-component from the inward- to the outward-facing orientation and its subsequent expulsion from the ECF module into the membrane. The reorientation of the S-component likely precedes dissociation, as the S-component remained associated with the ECF module for the duration of the simulation.

## Discussion

ABC transporters utilise free energy released by ATP binding, hydrolysis, and inorganic phosphate dissociation in a chemo-mechanical coupling mechanism to alternately expose the substrate binding site to either side of the membrane^1^. In well characterised canonical ABC transporters of Type I (e.g., the maltose importer MalFGK_2_)^35, 36^ and Type IV (e.g., the exporters MRP1^36^ and TmrAB^23,39^), the conformational switch between outward- and inward-exposed binding sites in the substrate-loaded state is described as a power stroke. For instance, in exporters, the binding of ATP, and associated closure of the ATPase dimer, directly powers the conversion form an inward-facing substrate-bound state to an outward-facing state^23,36,39^. This step also lowers the substrate binding affinity, so that release of the substrate on the outside is favoured, and the reverse transport is disfavoured. In the maltose importer, the release of inorganic phosphate and associated opening of the ATPase dimer powers the conversion of the outward-oriented maltose-bound transporter into an inward-facing state^36^, again with concomitant loss of substrate binding affinity, a mechanism that allows for accumulation of maltose in the cytoplasm.

**Fig. 4:**
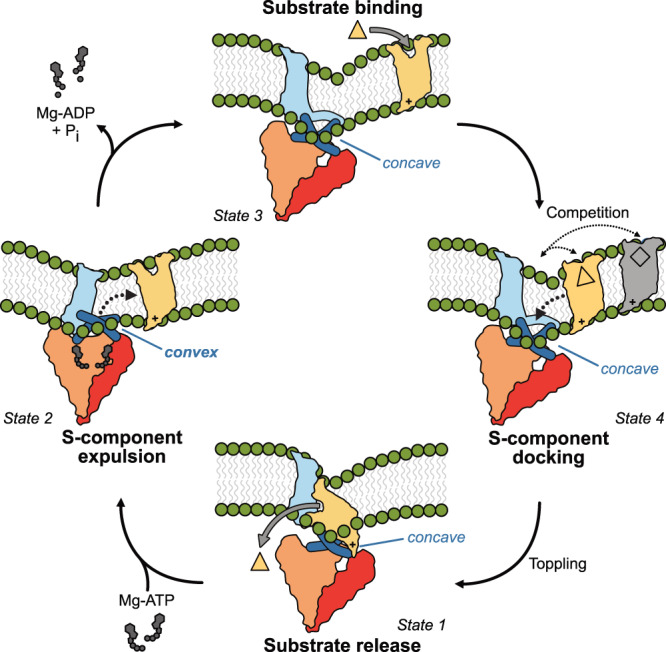
Bilayer-assisted chemo-mechanical coupling in the transport cycle of group II ECF transporters. Schematic representation of the different states in the transport cycle of group II ECF transporters with exchangeable S-components. State 1: The inward-facing apo conformation of the ECF transporter complex, from which substrate has been released into the cell. The S-component locally deforms the membrane to keep the positively charged base solvent exposed. The docking surface on the EcfT subunit for the S-component displays concave-shaped coupling helices. State 2: Upon binding of Mg-ATP, the ATPase dimer changes from an open to a closed configuration. This leads to a propagated change in the coupling helices that move into the membrane to adopt a convex shape (spring loaded), and to a reshaping of the local bilayer. Both features guide the reorientation to an outward-facing state and the expulsion of the S-component from the complex into the lipid bilayer. The EcfT transmembrane domain takes the space left by the expelled S-component into the membrane. State 3: Upon the hydrolysis of ATP and release of ADP and P_i_, the coupling helices return to their original concave shape with the EcfT transmembrane domain moving away from the docking site for the S-component, which restores the interaction surface (spring release) and creates space for the binding of a new S-component, for the same or a different substrate (competition). State 4: The solitary ECF module assumes a tilted conformation to locally thin and bend the membrane, guiding a substrate-bound S-component to topple within the membrane to form a stable complex with the ECF module.

ECF transporters use an entirely different mechanism for powering transport, which we summarise below (Fig. 4). The substrate-loaded S-component switches from an outward- to an inward-facing state (Fig. 4–right half), while the substrate-free S-component converts from an inward-facing to an outward-facing state (Fig. 4–left half), with the use of ATP conferring unidirectionality to the cycle (Fig. 4).

The inward-facing post-substrate-release state (Fig. 4–State 1), in which the S-component is trapped in the toppled conformation^4–10^, represents a structurally stable low energy state, as suggested by the numerous virtually identical structures obtained for this state so far. Consequently, energy input is required to reorient the toppled S-component and release its tight interaction with the ECF module. Our work here, uncovers the structural basis for this long sought-after key step in the transport cycle (Fig. 4–State 2 and Supplementary Movie 2). A relatively small conformational change upon Mg-ATP binding and closing of the ATPase dimer is amplified to a set of large motions in the EcfT subunit (Fig. 4–State 1 to 2 and Supplementary Fig. 15a, b). The coupling helices move into the membrane and shift from a concave to a convex shape, thereby disrupting the interaction surface (Fig. 1d, f and Supplementary Fig. 15a). This motion is markedly different than the previously proposed closing-scissor-like^10^ or sliding^12^ movement. Simultaneously, the transmembrane domains in the EcfT subunit move and rotate towards the coupling helices. Together, the two movements result in a structural mismatch of the interaction surface and will likely lead to the expulsion of the S-component from the ECF module into the lipid bilayer (Fig. 4–State 1 to 2). The ATP-driven expulsion mechanism presented here is consistent with biochemical data on the riboflavin-specific ECF transporter from *Listeria monocytogenes*^12^, showing that Mg-ATP binding and not ATP hydrolysis is required for the expulsion of the S-component in detergent solution^12^.

The ECF module with mutant Walker B motifs (EcfA E169Q and EcfA’ E’171Q) is trapped in a conformation with ATP bound to both sites. While this structure reveals how ATP binding leads to disruption of the tight interaction between the ECF module and the S-component, it is likely that binding of ATP to the two sites happens consecutively rather than simultaneously. Similarly, subsequent ATP hydrolysis may happen non-synchronously. An indication from such asymmetry comes from the structure of the nanodisc-reconstituted full complex ECF-FolT2_ATP_ determined here in the presence of ATP, resembling turnover conditions (Fig. 1a and Supplementary Fig. 3a). Intriguingly, this structure adopts an open conformation, but where ATP hydrolysis has occurred only in one of the sites, while ATP remained associated with the second site, and was apparently protected from hydrolysis (Supplementary Fig. 3a). We speculate that the S-component, which remains associated with the ECF module in the nanodisc environment, hinders the conformational transition needed for hydrolysis of the second ATP molecule. This observation may hint that S-component expulsion is required for subsequent ATP hydrolysis at the second site. Further studies will be needed to establish the order of binding and hydrolysis in the two sites.

Two models have been postulated for the process of substrate transport across the membrane (Fig. 4–right half)–namely the power stroke and thermal ratchet mechanism^2^. In the power stroke mechanism, it was proposed that a substrate-loaded S-component binds to the ECF module in the nucleotide-bound and closed conformation^2,12^; followed by ATP hydrolysis and opening of the ATPase dimer to power the toppling of the S-component. Instead, in the thermal ratchet mechanism, ATP hydrolysis and opening of the ATPase dimer occurs prior to the binding of a substrate-loaded S-component, and acts as a resetting step to restore the docking site for the S-component. Subsequently, toppling occurs sponteneously^2,4^. The data presented here is more consistent with the latter model. The structure of the ECF module in the closed conformation shows that the docking site for the S-component on the EcfT subunit dramatically changes shape upon ATP binding and closure of the ATPase dimer, making it unsuitable to keep the S-component bound to the motor (Fig. 4–State 1 to 2). In contrast, the surface on the S-component that interacts with the ECF module is rigid and does not change upon substrate binding^4^. Therefore, ATP hydrolysis and conversion to an open ATPase dimer are required to allow the ECF module to accommodate an S-component again (Fig. 4–State 2 to 3). Moreover, the imposed tension by the convex shape of the coupling helices in the closed conformation may not be energetically favourable and was captured due to the mutations introduced. Thus, the solitary ECF module in the open conformation likely represents an energetically more favourable post-ATP-hydrolysis and relaxed state (Fig. 4–State 3). It is noteworthy that the alternative power-stroke mechanism was proposed for the group I biotin ECF transporter BioMNY with a dedicated ECF module for a single S-component^40,41^, which may indicate that there is mechanistic diversity among ECF transporters.

The structures of substrate-bound and substrate-free FolT differ only in the conformation of two extracellular gating loops, leading to an open or occluded binding site (Fig. 4–State 3 and 4)^4^. The similarity between the two states raises the question what causes the substrate-free S-component to be expelled from the complex (Fig. 4–left half), yet the substrate-bound S-component to associate with the ECF module (Fig. 4–right half). In the substrate-free state the open gating loops expose a hydrophilic surface^4^. In the solitary outward facing conformation, these loops protrude to the aqueous extracellular side of the membrane. In the toppled conformation, these hydrophilic surfaces interact with the docking site of EcfT (Fig. 4–State 1 and 3). At the same time, the cytoplasmic base of S-components is positively charged^5^, and remains water-exposed in both conformations. Consequently, a reorientation of the substrate-free toppled S-component to an outward-facing, requires that both of hydrophilic extremes remain shielded from the hydrophobic part of the bilayer. The thin, bent bilayer formed around the docking site on the ECF module in the ATP-bound and closed conformation -but not the thick bilayer in the open state- matches the hydrophobic belt of the substrate-free S-component (Figs. 3b and 4–State 2 and 3). Therefore, reorientation and expulsion of the substrate-free S-component would be favoured by ATP binding (Fig. 3b and Supplementary Fig. 16d). In contrast, the substrate-bound S-component buries the hydrophilic gating loops into the protein interior^4^. The extracellular surface consequently becomes less hydrophilic, and may be more easily accommodated in the thick bilayer around the docking site found in the open conformation of the ECF module (Figs. 3a and 4–State 3 to 4), thereby favouring the substrate-bound S-component to topple over and dock to the ECF module, likely assisted again by the locally bent bilayer (Fig. 4–State 4 to 1). It is possible that continuous ATP turnover in the solitary ECF module (Fig. 2a) dynamically modulates and reshapes the membrane geometry to assist in S-component toppling. The synergy between local membrane morphology and conformational changes of the transporter could provide the means to reduce the energetic cost for the dynamic interplay between the ECF module and the S-component during every step of the transport cycle.

Upon expulsion, the S-component may linger around the ECF module or diffuse further out. It is possible that S-components for different substrates may differ in the range of diffusion, dependent on their exact shapes. Such structural differences could also explain observed variation in the kinetics of transport^6^, and ability to compete with other S-components for docking on the ECF module^3,5,6^. Similarly, in group I transporters such as BioMNY, the S-component may not be separated from the ECF module to a great extent^40,41^, explaining differences in apparent complex stability found in vitro experiments compared to group II transporters.

Our data suggests a model, where the energy harnessed from ATP binding and hydrolysis is used to reset the substrate-free transporter. ATP binding charges the coupling helices like springs in order to expel the S-component into the bilayer, while ATP hydrolysis is utilised to release the tension of the springs and modulates the membrane morphology in order to prepare for S-component docking and binding. The toppling and thermal ratchet mechanism is unprecedented and is a manifestation of the diversity of transport solutions adopted by the ABC transporter superfamily. The fundamental structural and mechanistic insights presented here may have wider implications, since exchange of S-components from different organisms upon horizontal gene transfer may contribute to the ability of bacteria to rapidly acquire new metabolic functionality, or even antibiotic resistance^42^.

## Methods

### Cloning

For the expression of the solitary ECF module, the operon (GenBank: CR954253.1–region 360354 to 362831) containing the genes encoding EcfA (Uniprot: Q1GBJ0), EcfA’ (Uniprot: Q1GBI9) and EcfT (Uniprot: Q1GBI8) from *L. delbrueckii* were cloned downstream of the first arabinose inducible promoter of the p2BAD expression vector into the multiple cloning site between the *Bsp*E1 and *Blg*II sites, in-frame with a sequence encoding an N-terminal deca-histidine tag and a TEV protease site (ENLYPQG)^4^. For the expression of ECF-FolT2, the gene (GenBank: CR954253.1–region 1400317 to 1400847) encoding FolT2 (Uniprot: Q1G292) from *L. delbrueckii* was cloned downstream of the second arabinose inducible promoter of the p2BAD expression vector into the multiple cloning site between the XbaI and XhoI sites, in-frame with a sequence encoding for a C-terminal STREPII tag (WSHPQFEK)^4^.

Site-directed mutagenesis was performed by overlap extension PCR to introduce glutamate-to-glutamine substitutions in the Walker B motifs of EcfA and EcfA’, and confirmed through Sanger sequencing by the Genomic Services of Eurofins Genomics. The primers used to introduce the substitutions are listed in Supplementary Table 1. We noted that the expression of the EcfA’ subunit in the 2EQ mutant variant was affected (Supplementary Fig. 7a). It was not possible to move the affinity tag onto EcfA’ as the genes are arranged within an operon, and purified protein could not be obtained when the affinity tag was placed on the carboxy-terminal end of EcfT. The subunit stoichiometry could be restored to wild-type levels (Supplementary Fig. 7a) by cloning in a second copy of the gene encoding EcfA’ E’171Q downstream of the second arabinose inducible promoter of the p2BAD expression vector into the multiple cloning site between the *Xba*I and *Xho*I sites (referred to as 2EQ_cryo_). The resulting plasmids were transformed into chemically competent *Escherichia coli* MC1061 cells.

For the expression of solitary FolT1, the gene (GenBank: CR954253.1–region 1399702 to 1400232) encoding FolT1 (Uniprot: Q1G930) from *L. delbrueckii* was cloned into the pREnHis vector downstream an N-terminal octo-histidine tag^4^, which was then converted into a *Lactococcus lactis* expression vector using the vector backbone exchange protocol^43^. The resulting plasmid was transformed into electrocompetent *L. lactis* NZ9000 cells.

### Protein expression and membrane vesicle preparation

ECF-FolT2, the ECF module and the ECF module mutant variants in *E. coli* MC1061 cells were grown in 5-L baffled flasks containing 2 L 2xYT liquid medium supplemented with 2.5 mM potassium phosphate (KPi), pH 7.5, 0.5% (v/v) glycerol, 100 µg/mL ampicillin, and 200 µL L^−1^ Antifoam 204 (Sigma-Aldrich) at 37 °C with shaking at 200 rpm for 2 h. The temperature was subsequently reduced to 25 °C, and protein expression was induced at an optical density of 600 nm of ∼0.8 with 0.01% (w/v) L-arabinose. Cells were harvested 3 h postinduction (7500 × *g*, 15 min, 4 °C), washed in storage buffer (50 KPi, pH 7.5, 10% (v/v) glycerol).

FolT1 in *L. lactis* NZ9000 cells were grown semi-anaerobically in rich medium containing 2% (w/v) Gistex LS (Strik BV), 65 mM KPi, pH 7.0, 1% (w/v) glucose and 5 µg/mL chloramphenicol in a 3-L bioreactor (Applikon Biotechnology) at 30 °C with a stirring speed of 200 rpm. Protein expression was induced at an optical density (OD_600_) of ~2 with a 1:500 dilution of the spent medium of the Nisin A producing *L. lactis* NZ9700 strain, and additional 1% (w/v) glucose was added. The pH was maintained at pH 6.5 with 4 M KOH during the growth. Cells were harvested 3 h postinduction (7500 × *g*, 15 min, 4 °C), washed in storage buffer (50 KPi, pH 7.5, 10% (v/v) glycerol).

For the preparation of membrane vesicles, the cell suspension was supplemented with 5 mM MgCl_2_ and 4 µg mL^−1^ DNaseI (Sigma-Aldrich), and lysed by two passages through a high-pressure homogeniser (HPL6, Maximator) at 25 kpsi for *E. coli* cells and 35 kpsi for *L. lactis* cells, 4 °C. Cell debris was removed by low speed centrifugation (27,500 × *g*, 60 min, 4 °C) and membrane vesicles were pelleted by ultracentrifugation (210,000 × *g*, 3 h, 4 °C). Membrane vesicles were homogenised in storage buffer (50 mM KPi, pH 7.5, 10% (v/v) glycerol) and *E. coli* and *L. lactis* membrane vesicles were stored in aliquots containing 60 and 30 mg total protein, respectively, as determined by the bicinchoninic acid assay kit (Thermo Fisher Scientific) at −80 °C after flash freezing in liquid nitrogen.

### Growth assays

The cobalamin-dependent growth assay was performed with the cobalamin-deficient *E. coli* ΔFEC strain^7^, which was co-transformed with two expression vectors in order to express the ECF module and the S-component. The p2BAD expression vector was used to express the ECF module variants and the pACYCara expression vector to express the S-component specific for cobalamin CbrT (Uniprot: Q1G7W0) or specific for folate FolT1. Pre-cultures of the strains carrying various expression vectors were grown in M9 medium supplemented 0.00001% (w/v) arabinose (Sigma-Aldrich) and 50 µg mL^−1^ methionine at 37 °C for 24 h, which were diluted 100-fold in fresh media to grow cells until an exponential growth phase was reached. Exponentially growing cells were diluted into 200 µL M9 medium supplemented with 0.00001% (w/v) arabinose in the presence of either 50 µg mL^−1^ methionine or 1 nM cyano-cobalamin (Acros Organics) to the same starting optical density at 600 nm (OD600) and grown in 96-well plates (Greiner) at 37 °C. The plates were sealed with sterile and gas-permeable foil (Breathe-Easy, Diversified Biotech) and the OD600 was measured every 10 min on a SpectraMax ABS Plus Microplate reader (Molecular Devices) for 36 h. The measurements were performed as biological triplicate containing each technical triplicate, and collected using the instruments’ SoftMax Pro version 7.1.2 (Molecular Devices).

### Purification

All purification steps were performed at 4 °C. For the purification of ECF-FolT2^5^, membrane vesicles containing 60 mg total protein were buffered in 50 mM KPi, pH 7.5, 300 mM NaCl, 1% (w/v) dodecyl-β-maltoside (DDM, Glycon), and solubilized for 1 h at the lowest speed on a rotator. The solubilised sample was clarified by ultracentrifugation at 286,625 × *g* for 35 min, and the supernatant was incubated with 0.5 mL Nickel-Sepharose 6 Fast Flow beads (Cytiva) for 1 h at the lowest speed on a rotator, which were previously equilibrated with wash buffer (50 mM KPi, pH 7.5, 300 mM NaCl, 50 mM imidazole, pH 8.0, 0.05% (w/v) DDM). The suspension was packed into an empty polypropylene column (Bio-Rad), and the beads were washed with 30 column volumes wash buffer. Bound proteins were eluted with elution buffer (50 mM KPi, pH 7.5, 300 mM NaCl, 500 mM imidazole, pH 8.0, 0.04% (w/v) DDM) in 250-, 850-, and 250-µL fractions. The second elution fraction was used for further purification by size-exclusion chromatography (SEC) using a Superose 6 Increase 10/300 GL column (Cytiva) on an ÄKTA Pure 25 (Cytiva) equilibrated with SEC buffer (20 mM Tris, pH 8.0, 150 mM NaCl, 0.0261% (w/v) DDM). Peak fractions containing ECF-FolT2 were pooled and used for reconstitution into lipid nanodiscs.

For the purification of the solitary ECF module variants, the steps were the same as for the purification of ECF-FolT2. After the wash step, the beads were resuspended in a final volume of 1 mL wash buffer and the ECF module was recovered from the beads by on-column digestion with 700 µg of home-made tobacco etch virus (TEV) protease in the presence of an additional 50 mM imidazole, pH 8.0 and 5 mM dithiothreitol (DTT) overnight for 14 h. The mobile phase with the untagged ECF module was separated by gravity flow from the resin with empty Bio-Spin columns (Bio-Rad). An additional 0.4 mL wash buffer was passed through to recover residual protein. The recovered sample was purified further by SEC using a Superdex 200 Increase 10/300 GL column (Cytiva) equilibrated with SEC buffer (20 mM Tris, pH 8.0, 150 mM NaCl, 0.017% (w/v) DDM) on an ÄKTA Pure 25 or ÄKTA Go (Cytiva). Peak fractions containing the ECF module were pooled and used either for assessing the activity in liposome-based transport assays, for cryo-EM of the ECF module in detergent micelles, or for reconstitution into lipid nanodiscs. For the purification of the mutant ECF module variant 2EQ_cryo_ destined for cryo-EM, 1 mM ATP (Roche) and 1 mM MgCl_2_ were added during solubilisation and in the wash buffer. Representative SEC elution profiles and Coomassie blue-stained sodium dodecyl sulfate–polyacrylamide gel electrophoresis (SDS-PAGE) gel images of the peak fraction are shown in Supplementary Fig. 7a.

Higher yields were obtained with FolT1 compared to FolT2 (~93% sequence identity and similar binding affinities for folate). For the purification of FolT1^4^, membrane vesicles containing 30 mg total protein were buffered in 50 mM KPi, pH 7.0, 200 mM KCl, 1% (w/v) DDM), and solubilised for 1 h on a rotator at the lowest speed. The solubilised sample was clarified by ultracentrifugation at 286,625 × *g* for 35 min, and the supernatant was incubated with 0.5 mL Nickel-Sepharose 6 Fast Flow beads for 1 h at the lowest speed on a rotator, which were previously equilibrated with wash buffer (50 mM KPi, pH 7.0, 200 mM KCl, 50 mM imidazole, pH 8.0, 0.05% (w/v) DDM). The suspension was packed into an empty polypropylene column, and the beads were washed with 30 column volumes wash buffer. Bound proteins were eluted with elution buffer (50 mM KPi, pH 7.0, 200 mM KCl, 500 mM imidazole, pH 8.0, 0.04% w/v DDM) in 250, 850, and 250 μL fractions. The second elution fraction was used for further purification by SEC using a Superdex 200 Increase 10/300 GL column equilibrated with SEC buffer (50 mM KPi, pH 7.0, 150 mM KCl, 0.017% (w/v) DDM) on an ÄKTA Pure or ÄKTA Go. Peak fractions containing FolT1 were pooled and used for assessing the activity in liposome-based transport assays. A representative SEC elution profile and a Coomassie blue-stained sodium dodecyl sulfate–polyacrylamide gel electrophoresis (SDS-PAGE) gel image of the peak fraction is shown in Supplementary Fig. 7a.

### Reconstitution into liposomes

*E. coli* polar lipid extract (Avanti Polar Lipids) and egg phosphatidylcholine (Avanti Polar Lipids) dissolved in chloroform were mixed at a 3:1 ratio and dried under a stream of nitrogen gas. The obtained lipid film was dissolved to a concentration of 20 mg ml^−1^ in 50 mM KPi, pH 7.5 buffer, and then extruded eleven times through a 400 nm polycarbonate filter (Avestin). The liposomes were destabilised by adding Triton X-100, until half of the initial absorbance at 540 nm was reached. Freshly purified proteins were reconstituted at a 1:250 protein: lipid ratio (w/w). In the case of the co-reconstitution of the ECF module and the S-component, the protein: lipid ratio for each protein was maintained separately, which resulted in a ~4.5 fold molar excess of the S-component compared to the ECF module. The protein-lipid suspension was incubated for 30 min at room temperature, after which 32 mg of Bio-Beads SM-2 (Bio-Rad) per 1 ml suspension were added and incubated for another 30 min. After a second addition of 19 mg of Bio-Beads per 1 ml suspension, the incubation was continued at 4 °C for 60 min followed by an overnight incubation at 4 °C with another addition 24 mg of Bio-Beads per 1 ml suspension. On the next day, the suspension was incubated with a final addition of 36 mg of Bio-Beads per 1 ml suspension for 60 min at 4 °C. The proteoliposomes were collected by ultracentrifugation at 336,896 × *g* at 4 °C for 30 min, and aliquots were flash-frozen and stored in liquid nitrogen until use.

### Folate uptake assay with proteoliposomes

Proteoliposomes were initially thawed, supplemented with Mg-ATP or Mg-ADP (Sigma-Aldrich) to a final concentration of 5 mM, and then subjected to three freeze-thaw cycles in order to enclose the nucleotides in the proteoliposome lumen. Proteoliposomes were extruded eleven times through a 400 nm polycarbonate filter to obtain a homogenous size distribution. Nucleotides on the exterior of the proteoliposomes were removed by washing with 50 mM KPi, pH 7.5 and ultracentrifugation steps at 4 °C for 30 min. Proteoliposomes were resuspended in 50 mM Kpi, pH 7.5 to a final protein concentration of 0.8 µg protein per µL. Liposomes containing 1 µg ECF module only, 1 µg FolT1 only or 2 µg ECF module and FolT1 were added to 200 µL 50 mM KPi, pH 7.5 containing 100 nM folate (95 nM cold folate and 5 nM radiolabelled [3, 5, 7, 9-3 H] folate (American Radiolabeled Chemicals)). The reaction was incubated at 30 °C for 0, 1.5, 3, 4.5 and 9 min, after which 2 mL ice-cold 50 mM KPi, pH 7.5 was added and filtered over a BA-85 nitrocellulose filter (Cytiva). Filters were washed once more with 50 mM KPi, pH 7.5, and dissolved subsequently in 1.8 mL Filter Count Scintillation liquid cocktail (Perkin Elmer). The accumulation of radioactivity into the proteoliposome lumen was determined with a Perkin Elmer Tri-carb 2800TR Scintillation counter. The measurements were performed as biological duplicate containing each technical duplicate with the exception of the FolT1 only sample, which was performed once containing a technical duplicate.

### Reconstitution into lipid nanodiscs

All reconstitution steps were performed at 4 °C if not stated otherwise. The membrane scaffold protein MSP2N2 was purified according to established protocols^44^. *E. coli* polar lipid extracts in chloroform were vacuum dried and rehydrated in 20 mM Tris, pH 8.0, 150 mM NaCl, 1.75% (w/v) DDM to a concentration of 20 mg mL^−1^. For the reconstitution of ECF-FolT2 into lipid nanodiscs, freshly purified protein was mixed with purified untagged MSP2N2 and detergent-solubilised lipids at a 1:5:250 molar ratio of ECF-FolT2: MSP2N2: lipids and kept on ice for 30 min, after which 200 mg mL^−1^ washed Bio-Beads were added and left to incubate at 4 °C on a rotator at the lowest speed. Another portion of 200 mg/mL Bio-Beads was added and left to incubate for 16 h overnight. The reconstitution mixture was separated from the Bio-Beads and incubated with 0.5 mL Nickel-Sepharose 6 Fast Flow beads for 1 h at 4 °C at the lowest speed on a rotator, which were previously equilibrated with wash buffer (20 mM Tris, pH 8.0, 150 mM NaCl). The suspension was packed into an empty polypropylene column, and the beads were washed with 40 column volumes wash buffer. Bound proteins were eluted with elution buffer (20 mM Tris, pH 8.0, 150 mM NaCl, 500 mM imidazole, pH 8.0) in 250-, 850-, and 250-μL fractions. The second elution fraction was used for further purification by SEC using a Superose 6 Increase 10/300 GL column equilibrated with SEC buffer (20 mM Tris, pH 8.0, 150 mM NaCl) on an ÄKTA Pure 25 system. Peak fractions containing the ECF-FolT2 in lipid nanodiscs were pooled and used for grid preparation.

For the reconstitution of the solitary ECF module into lipid nanodiscs, the freshly purified protein was mixed with untagged MSP2N2 and detergent-solubilised lipids at a 1:2:100 molar ratio of ECF module: MSP2N2: lipids, and the initial steps were the same as for the reconstitution of ECF-FolT2. After an overnight incubation for 19 h at 4 °C, the reconstitution mixture was separated from the Bio-Beads, and further purified by SEC using a Superdex 200 Increase 10/300 GL column equilibrated with SEC buffer (20 mM Tris, pH 8.0, 150 mM NaCl) on an ÄKTA Pure 25. Peak fractions containing the ECF module in lipid nanodiscs were pooled and used either for assessing the activity of the ATPases by an enzyme-coupled spectrophotometric assay or for cryo-EM. For the reconstitution of the mutant ECF module variant 2EQ_cryo_ destined for cryo-EM, the same reconstitution ratios and steps were used with minor changes during the initial stages. The freshly purified protein was incubated with 1 mM ATP, 1 mM MgCl and lipids for 30 min at 10 °C on a nutator, and then for 15 min at room temperature. Purified untagged MSP2N2 was added to the sample and incubated for 15 min at room temperature, after which 200 mg mL^−1^ washed Bio-Beads were added and left to incubate for 30 min at room temperature. Another portion of 200 mg mL^−1^ washed Bio-Beads were added and left to incubate for 19 h overnight. Representative SEC elution profiles and Coomassie blue-stained sodium dodecyl sulfate–polyacrylamide gel electrophoresis (SDS-PAGE) gel images of the final sample are shown in Supplementary Fig. 7b.

### ATPase activity assay

The ATPase activity of various ECF module variants in lipid nanodiscs was determined using an enzyme-coupled spectrophotometric assay^45^. The assay was performed in 96-well plates (Greiner) with a final volume of 200 µL containing 20 mM Tris, pH 8.0, 150 mM NaCl, 0.3 mM NADH (Roth), 4 mM phosphoenolpyruvate (Roth), 3.5 µL pyruvate kinase-lactic dehydrogenase solution (Sigma-Aldrich) corresponding to 2.1 to 3.5 units pyruvate kinase and 3.2 to 4.9 units lactic dehydrogenase, varying concentrations of Mg-ATP (0, 0.05, 0.1, 0.2, 0.3, 0.4 and 0.5 mM), and 1.1 µg of nanodisc-embedded ECF module variants. The ATPase activity was determined by monitoring the decrease of NADH over time by measuring the absorbance at 340 nm on a Spark 10 M microplate reader (TECAN) at 37 °C. The concentration of NADH was subsequently calculated by applying the Beer-Lambert law using the extinction coefficient of 6.22 cm^−1^ mM^−1^ at 340 nm. The pathlength of light through the solution was determined by measuring the absorbance (A) of 200 µL water at 900 and 977 nm and using the formula (A_977nm_–A_900nm_) x 0.18^−1^ cm. The measurements were performed as biological duplicate containing each technical triplicate, and collected using the instruments’ SparksControl software version 2.3 (TECAN).

### Cryo-EM sample preparation and data acquisition

The peak fractions containing ECF-FolT2 in lipid nanodiscs were pooled and concentrated to 6.2 to 7.5 mg mL^−1^ with a 100-kDa molecular weight cut-off (MWCO) centrifugal concentrator (Millipore). For ECF-FolT2_ATP_, 5 mM Mg-ATP and 10 µM folate were added to the concentrated sample and incubated at 37 °C for 10 min. For ECF-FolT2_AMP-PNP_, 5 mM Mg-AMP-PNP (Roche) and 10 µM folate were added to the concentrated sample and incubated at 37 °C for 10 min. For the wild-type ECF module in lipid nanodiscs, peak fractions were pooled and concentrated to 4.2 to 6.5 mg mL^−1^ with a 100-kDa MWCO centrifugal concentrator (Supplementary Fig. 7c). For the wild-type ECF module in DDM micelles, peak fractions were pooled and concentrated to 5.7 mg mL^−1^ with a 100-kDa MWCO centrifugal concentrator (Supplementary Fig. 7d). For the mutant ECF module variant 2EQ_cryo_ in lipid nanodiscs, fractions of peak 1 and peak 2 were pooled separately and concentrated to 4.9 and 4.6 mg mL^−1^ with a 100-kDa MWCO centrifugal concentrator (Supplementary Fig. 7e), respectively.

Holey-carbon cryo-EM grids (Quantifoil Au R1.2/1.3 300-mesh) were glow-discharged (Edwards Scancoat 6) at 5 mA for 45 s. In all cases prior to sample application, fluorinated Fos-choline 8 (Anatrace) was added to a final concentration of 2.9 mM to obtain an even distribution of well-separated particles. Samples were immediately applied at a volume of 2.5 µL onto grids, blotted for 3.5–4 s using a Vitrobot Mark IV (Thermo Fisher Scientific) at 15 °C and 100% humidity, and subsequently plunge frozen in a liquid ethane-propane mixture and stored in liquid nitrogen until use.

Datasets ECF-FolT2_ATP_, ECF-FolT2_AMP-PNP_, wild-type ECF module in lipid nanodiscs and DDM micelles were collected in-house on a Talos Arctica cryo-TEM (Thermo Fisher Scientific) operating at 200 keV with a BioQuantum post-column energy filter (Gatan) in zero-loss mode and a 20-eV slit width, and a 100 µm objective aperture on a K2 Summit direct electron detector (Gatan) in counting mode. Movies were recorded in an automated fashion with EPU version 2.7.0 (Thermo Fischer Scientific), or SerialEM version 3.8.0 beta or 3.9.0 beta using a 3 × 3 multi-shot array^46,47^, at a physical pixel size of 1.022 Å (calibrated magnification of 48,924x, nominal magnification 130,000x), a defocus range from −0.8 to −1.8 μm, an exposure time of 9 s with a subframe exposure time of 150 ms (60 frames), and a total electron exposure on the specimen level of 50.1 electrons per Å2. Optimal regions for data acquisition on the grid were screened and selected with an inhouse–written script to calculate the ice thickness with a Digital Micrograph (Gatan) script when EPU was used or through a script implemented in SerialEM^48^, which was set between 30 and 50 nm. The data quality was monitored on-the-fly using the software FOCUS version 1.1.0^49^. For the mutant ECF module 2EQ_cryo_, initial datasets were also collected in-house from grids with samples from peak 1 and 2 with the same setup as described above, which provided initial reconstructions at 7.1 and 4.9 Å resolution, respectively, revealing a closed conformation of the ATPase dimer. The final dataset for the mutant ECF module 2EQ_cryo_ was collected from grids containing peak 2 on Krios 1 (Titan Krios G1 cryo-TEM, Thermo Fisher Scientific) at the Netherlands Centre for Electron Nanoscopy (NeCEN) operating at 300 keV with a BioQuantum post-column energy filter (Gatan) in zero-loss mode and a 20-eV slit width, and a 100 µm objective aperture on a K3 BioQuantum direct electron detector (Gatan) in super-resolution counting mode with hardware binning by a factor of 2 (Counted Super Resolution Bin 2). Movies were recorded in an automated fashion with EPU version 2.8.1 (Thermo Fisher Scientific), with Aberration Free Image Shift (AFIS) enabled, with a physical pixel size of 0.834 Å (calibrated magnification of 59,809x, nominal magnification 105,000x), a defocus range from −0.9 to −1.9 µm, an exposure time of 2.6 s with a subframe exposure time of 35 ms (75 frames), and a total electron exposure on the specimen level of 60 electrons per Å2. Optimal regions for data acquisition on the grid as described above with a Digital Micrograph script^48^, and two exposures per foil hole were recorded. The data quality was monitored on-the-fly using the software Warp version 1.0.9^50^.

### Cryo-EM image processing

All movies from each dataset were subjected to motion correction and dose weighting of frames in MotionCor2 version 1.4.0^51^. The CTF parameters were estimated on the movie frames with CTFFIND4.1.14^52^. Low quality images were removed on the basis of visual inspection of the images showing contamination and poor CTF estimation, and duplicate exposures were removed using an in-house written script. The remaining images were used for further processing. Motioncor2 and CTFFIND4 were executed within the FOCUS software. Particles were picked with crYOLO version 1.7.5, 1.7.6 or 1.8.2^53,54^ using the PhosaurusNet architecture with anchors set to 180 for the ECF-FolT2 datasets and 160 for the ECF module datasets. Models were created and trained on selected micrographs, which were subsequently used for picking particles on all micrographs with a relaxed threshold of 0.2. Particles were extracted with imported particle coordinates from crYOLO in RELION version 3.1.3^55^, which were subsequently imported into cryoSPARC version 3.2 or 3.3 for further processing^56^. Bayesian polishing^57^ and 3D classification steps without image alignment were performed in RELION. The UCSF PyEM collection of Python scripts^58^ was used to convert cryoSPARC output files to STAR files for import into RELION (csparc2star.py) and to create Euler angle distribution plots (star2bild.py). All reported resolutions were estimated with the 0.143 cut-off criterion^59^ with gold-standard Fourier shell correlation (GS-FSC) between two independently refined half maps^60^. During sharpening, high-resolution noise substitution was used to correct for convolution effects of real-space masking on the FSC curve^61^. The directional resolution anisotropy of density maps was quantitatively evaluated using the remote 3DFSC processing server (accessed at https://3dfsc.salk.edu)^62^. Local resolution variations were estimated in cryoSPARC.

### ECF-FolT2_ATP_ in lipid nanodiscs

A total of 502 and 597 movies were recorded with EPU from two grids, and 2187 movies were recorded with SerialEM from a third grid. After manual curation, 498, 487 and 2006 movies, respectively, remained for further processing. Particles were picked from each dataset, merged and a total of 1,207,115 extracted particles with a box size of 256 pixels were subjected to two rounds of 2D classification. A total of 561,582 particles underwent a heterogenous refinement step with seven Ab initio reconstructions as input. The best class containing 167,277 particles was subjected to a round of non-uniform refinement resulting in a 3.5 Å resolution reconstruction, which was improved to 3.3 Å resolution by a Bayesian polishing step. A further improvement was obtained using global CTF refinement based on optics group resulting in the final map at 3.22 Å resolution with a global B-factor of −112.9 Å2. The final cryo-EM data processing workflow is summarised in Supplementary Figs. 1 and 2, and the data collection and processing parameters are summarised in Supplementary Table 2.

### ECF-FolT2_AMP-PNP_ in lipid nanodiscs

A total of 4052 movies were recorded with SerialEM. After manual curation, 3527 movies remained for further processing. Particles were picked and a total of 854,709 extracted particles with a box size of 256 pixels were subjected to two rounds of 2D classification. A total of 425,222 particles underwent a heterogenous refinement step with seven Ab initio reconstructions as input. The best class containing 129,663 particles was subjected to a round of non-uniform refinement resulting in a 3.8 Å resolution reconstruction, which was improved to 3.7 Å resolution using local and global CTF refinement on particle defocus and optics group, respectively. A Bayesian polishing step further improved the resolution to 3.6 Å. The final map was obtained after another round of global and local CTF refinement on optics group and particle defocus, respectively, at 3.58 Å resolution with a global B-factor of −117.9 Å2. The final cryo-EM data processing workflow is summarised in Supplementary Figs. 4 and 5, and the data collection and processing parameters are summarised in Supplementary Table 2.

### Wild-type solitary ECF module in lipid nanodiscs

A total of 2200, 4608, 2101, and 3162 movies were recorded with SerialEM from four grids. After manual curation, 1400, 4205, 1401 and 2981 movies, respectively, remained for further processing. Particles were picked from each dataset, merged and a total of 2,889,054 extracted particles with a box size of 256 pixels were subjected to three rounds of 2D classification. A total of 1,868,626 particles underwent a heterogenous refinement step with five Ab initio reconstructions as input. The best class containing 358,419 particles was subjected to a round of non-uniform refinement resulting in a 4.2 Å resolution reconstruction, which subjected to another round of heterogenous refinement with two Ab initio reconstructions as input. The best class containing 297,970 particles was subjected to a round of non-uniform refinement, which was followed by a Bayesian polishing step resulting in an improved map at 4 Å resolution. A particle set containing 107,197 particles was isolated by 3D classification without alignment, which resulted after another round of heterogenous refinement with two Ab initio reconstructions as input in a 3.8 Å resolution reconstruction containing 93,755 particles. The final map was obtained after a round of global CTF refinement on optics group at 3.78 Å resolution with a global B-factor of −148.4 Å2. The final cryo-EM data processing workflow is summarised in Supplementary Figs. 8 and 9, and the data collection and processing parameters are summarised in Supplementary Table 2.

### Wild-type solitary ECF module in DDM micelles

A total of 1906 movies were recorded with SerialEM. After manual curation, 1862 movies remained for further processing. Particles were picked and a total of 624,168 extracted particles with a box size of 256 pixels were subjected to two rounds of 2D classification. A total of 154,619 particles underwent a heterogenous refinement step with three Ab initio reconstructions as input. The best class containing 84,850 particles was subjected to a round of non-uniform refinement resulting in a 5.4 Å resolution reconstruction, which was subjected to another round of heterogenous refinement with two Ab initio reconstructions as input. The best class containing 54,660 particles was subjected to a round of non-uniform refinement resulting in a 4.5 Å resolution reconstruction. The final map was obtained after a Bayesian polishing step at 4.25 Å resolution with a global B-factor of −159.4 Å2. The final cryo-EM data processing workflow is summarised in Supplementary Fig. 11, and the data collection and processing parameters are summarised in Supplementary Table 2.

### Mutant solitary ECF module 2EQ in lipid nanodiscs

A total of 12,169 and 7444 movies were recorded with EPU from two grids. After manual curation, 9932 and 5326 movies, respectively, remained for further processing. Particles were picked from each dataset and a total of 3,275,263 and 1,634,963 extracted particles with a box size of 320 pixels and downscaled to 1.672 Å/pixel resulting in a box size of 160 pixels, respectively, were subjected to two rounds of 2D classification. A total of 2,302,271 and 744,495 particles, respectively, underwent a heterogenous refinement step with seven Ab initio reconstructions as input. The best classes containing 724,428 and 227,064 particles, respectively, were subjected to a round of non-uniform refinement resulting both in 3.5 Å resolution reconstructions. Particles from both datasets were re-extracted and re-centred with a box size of 320 pixels without downscaling, merged, and subjected to a round of non-uniform refinement resulting in a 3.1 Å resolution reconstruction. A Bayesian polishing step further improved the resolution to 3 Å resolution. Another round of heterogenous refinement with five Ab initio reconstructions as input improved the map to 2.9 Å resolution containing 713,098 particles. A particle set containing 270,770 particles was isolated by 3D classification without alignment resulted in a 2.8 Å resolution reconstruction. Another round of Bayesian polishing with particles re-extracted with a box size of 386 pixels resulted in a further improvement to 2.7 Å resolution. The final map was obtained after a round of local and global CTF refinement on particle defocus and optics group at 2.6 Å resolution with a global B-factor of −89.8 Å2. The final cryo-EM data processing workflow is summarised in Supplementary Figs. 13 and 14, and the data collection and processing parameters are summarised in Supplementary Table 2.

### Model building and validation

All models were build and modified in COOT version 0.9.8.1^63^. Density maps sharpened with DeepEMhancer^64^ were used only to guide the model building process and for preparing the final figures. All models were subjected to an iterative process of real-space refinement against the cryoSPARC-sharpened map in PHENIX version 1.20.1-4487^65,66^. All programs used for image processing were managed through the SBGrid software manager version 2.5.6^67^. The atomic model of ECF-FolT2 in the apo state (PDB: 7NNU) was fitted into the density of ECF-FolT2_ATP_ and ECF-FolT2_AMP-PNP_ with UCSF ChimeraX version 1.3^68^, which was adjusted and improved in COOT. Chain A, B and D of the atomic model of ECF-FolT2 was used for the wild-type solitary ECF module. For the solitary ECF module 2EQ_cryo_ mutant variant, the atomic model of the wild-type solitary ECF module was used as a starting model. In order to accommodate the observed conformational changes, the model was subjected to rigid body movements were applied and manual adjustment and rebuilding. Mg^2+^, and the nucleotides ADP, ATP and AMP-PNP were modelled into the respective density maps with COOT. Water molecules were added to the model of solitary ECF module 2EQ_cryo_ using phenix.douse as part of the PHENIX program, which were subsequently manually curated and retained based on logical interaction geometries and clear and visible densities. The chemo-physical properties of the final models were validated with MolProbity^69^. Refinement parameters are summarised in Supplementary Table 2.

### Coarse-grained molecular dynamics simulations

All simulations were performed with GROMACS version 2020.7 using the Martini 3 coarse-grained model^70^, which is well suited to capture protein induced membrane deformations^20,71,72^. The solitary wild-type ECF module and the mutant solitary ECF module 2EQ_cryo_ were modelled from the cryo-EM reconstructions obtained in this study. The wild-type full ECF transporter complex containing all the four subunits was modelled from the cryo-EM reconstruction of the folate-specific ECF transporter complex (PDB: 7NNU)^5^. For modelling the full ECF transporter complex with the ATPase dimer in the closed conformation, the mutant solitary ECF module 2EQ_cryo_ was first aligned to the wild-type full ECF transporter complex, and combined with the S-component from the wild-type full complex. A multistep minimisation processes was used to resolve structural clashes. All coarse-grained protein models were constructed using Martinize 2, with an applied elastic network with a bond force constant of 700 kJ mol^−1^ nm^−2^ and a cut-off distance of 0.85 nm. Side chain corrections were applied^73^. All protein models were positioned in the membrane as described in previous simulation studies^20^. Initial structures were built and solvated using the inane.py coarse-grained building tool^74^, by arranging the lipids and proteins on a regular array in the bilayer (xy) plane to obtain roughly 325 lipids per leaflet, solvated by roughly 19,000 regular martini water beads in a 15 × 15 × 15 nm simulation box. Counter ions were added to neutralise the systems as necessary, plus 140 mM NaCl ionic strength. The membrane was composed of POPE (1-palmitoyl-2-oleoyl-sn-glycero-3-phosphoethanolamine), POPG (1-palmitoyl-2-oleoyl-sn-glycero-3-phosphoglycerol) and CL (cardiolipin) lipids at a 70:25:5 mol % ratio. This composition is consistent with our previous simulation studies^20^, allowing the validation of the Martini 3 simulations performed here with previous results obtained with atomistic simulations of the open conformation. Non-bonded interactions were cut off at 1.1 nm, and Coulombic interactions were treated using reaction-field electrostatics^75^, with a dielectric constant of 15 and an infinite reaction-field dielectric constant. The particle neighbour list was updated using the Verlet list scheme. A v-rescale thermostat was used^76^, with a coupling time of 4.0 ps to main the temperature at 303 K. Separate temperature coupling was used for the bilayer, the protein and the solvent. Constant pressure was semi-isotropically coupled to 1.0 bar using the Parrinello-Rahman barostat^77^, with a relaxation time of 16.0 ps. After initial energy minimization and pressure/temperature equilibration rus, simulations were run at 20 fs time steps. Solitary ECF module simulations were run for at least 15 µs. For full ECF transporter complex simulations, 5 replicas were run for at least 15 µs. The tilt angles of the S-component in the full ECF transporter complex simulations was determined as the angle between the vector that goes through the helix comprised of residues 106 to 134 of the S-component and the membrane plane, which was approximated as the simulation box z-axis at which no substantial membrane deformations occur. All simulations were analysed making use of in-house developed Python 3 programs using the MDAnalysis package version 2.2.0^78^. IPython version 8.4.0^79^, NumPy version 1.12.0^80^, SciPy version 1.8.1^81^, Scikit-Learn version 1.1.1^82^, Voro + + version 0.4.6^83^, and Matplotlib version 3.5.2^84^ packages for scientific computing in Python version 3.8.10 were also used. Visualization and rendering of the simulations were performed with the molecular graphics viewer VMD version 1.9.4^85^.

### Figure preparation

Figures were prepared with UCSF ChimeraX version 1.3, VMD version 1.9.4 and Prism version 9.3.1 (GraphPad Inc). Final illustrations were prepared in Affinity Designer version 1.10.5 (Serif Ltd).

### Reporting summary

Further information on research design is available in the Nature Portfolio Reporting Summary linked to this article.

## Supplementary information

Supplementary information

Peer Review file

Description of Additional Supplementary Files

Supplementary Movie 1

Supplementary Movie 2

Reporting Summary

## Data Availability

Cryo-EM density maps, half maps, and masks have been deposited in the Electron Microscopy Data Bank (EMDB) under accession numbers 16120 (ECF-FolT2_ATP_), 16121 (ECF-FolT2_AMP-PNP_), 16122 (ECF module WT_MSP2N2_), 16123 (ECF module WT_DDM_), and 16124 (ECF module 2EQ_cryo_). Models are available through the Protein Data Bank (PDB) under the accession codes 8BMP (ECF-FolT2_ATP_), 8BMQ (ECF-FolT2_AMP-PNP_), 8BMR (ECF module WT_MSP2N2_), and 8BMS (ECF module 2EQ_cryo_). Raw movies have been deposited in the Electron Microscopy Public Image Archive (EMPIAR) under accession numbers 11307 (ECF-FolT2_ATP_), 11308 (ECF-FolT2_AMP-PNP_), 11309 (ECF module WT_MSP2N2_), 11310 (ECF module WT_DDM_), and 11311 (ECF module 2EQ_cryo_). The previously resolved structure of ECF-FolT2 in the apo state used in this study is available through the PDB under the accession code 7NNU. DNA sequences used in this study are available through GenBank under the accession code CR954253.1 with regions 360354 to 362831, 1399702 to 1400232, and 1400317 to 1400847 corresponding to the ECF module operon, FolT1, and FolT2, respectively. Protein sequences used in this study are available through UniProt under the accession codes Q1GBJ0 for EcfA, Q1GBI9 for EcfA’, Q1GBI8 for EcfT, Q1G930 for FolT1, Q1G292 for FolT2, and Q1G7W0 for CbrT. For the coarse-grained molecular dynamics simulations, data (final snapshots, cleaned trajectories, starting structure/simulation parameters) are available through the open repository Zenodo [10.5281/zenodo.8116403]. Source data are provided with this paper.

## Source data

Source data file
