# Supplementary material for: Expulsion mechanism of the substrate-translocating subunit in ECF transporters
Source: Nat Commun. 2023 Jul 25;14:4484. doi: 10.1038/s41467-023-40266-1 (PMC10368641; doi:10.1038/s41467-023-40266-1)
Supplement: Supplementary file 1 — Supplementary information [file 41467_2023_40266_MOESM1_ESM.pdf]

Supplementary Information

Expulsion mechanism of the substrate-translocating subunit in ECF transporters

Chancievan Thangaratnarajah, Mark Nijland, Luís Borges-Araújo, Aike Jeucken, Jan Rheinberger, Siewert J. Marrink, Paulo C.T. Souza, Cristina Paulino, Dirk J. Slotboom

Correspondence: C.P. (cristina.paulino@bzh.uni-heidelberg.de) and D.J.S. (d.j.slotboom@rug.nl)

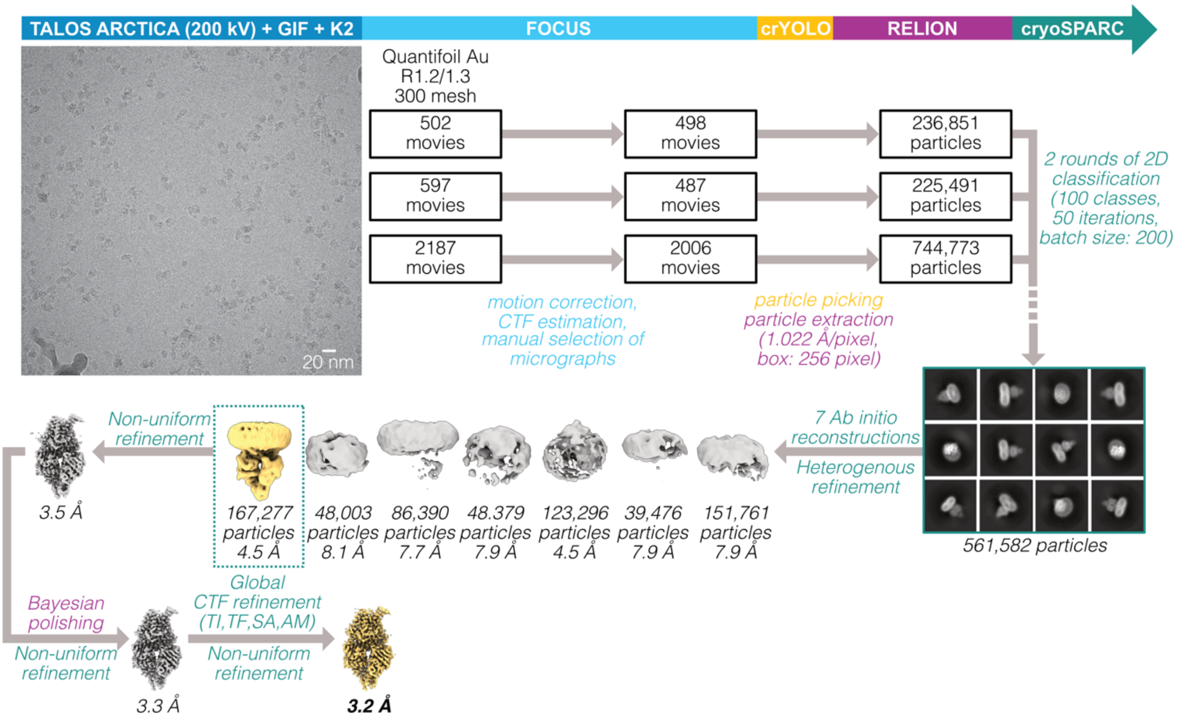

Supplementary Fig. 1 – Cryo-EM reconstruction of ECF-FolT2$_{ATP}$ reconstituted into lipid nanodiscs. Detailed image processing workflow including a representative micrograph at a defocus of volume of -1.4 µm showing the particle distribution of ECF-FolT2$_{ATP}$-MSP2N2 in the presence of 2.9 mM Fos-choline 8 on a Quantifoil Au R1.2/1.3 300 mesh grid. Abbreviations correspond to following: TI = Tilt, TF = Trefoil, SA = Spherical Aberration, AM = Anisotropic magnification.

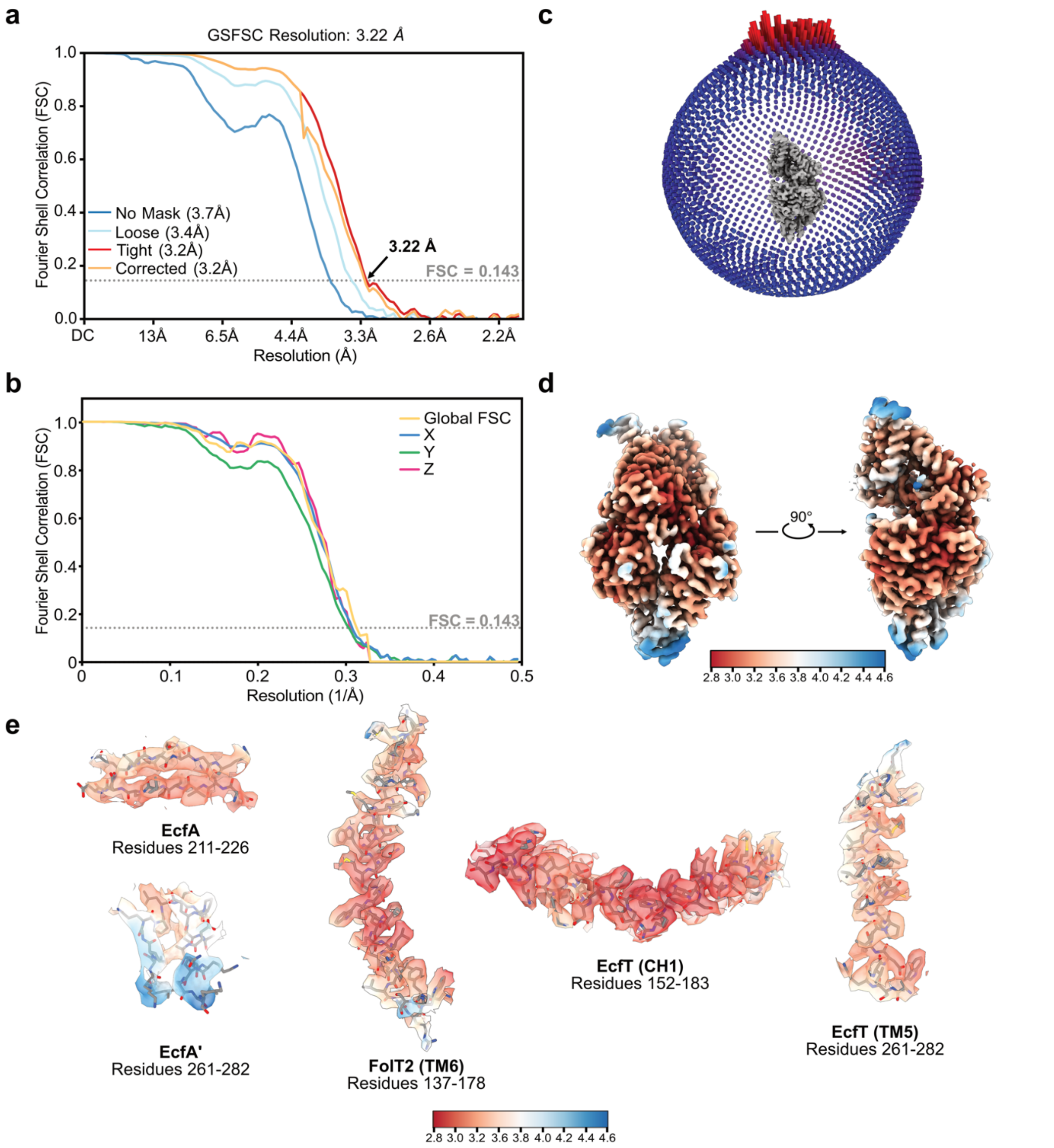

Supplementary Fig. 2 – Validation of the cryo-EM reconstruction of ECF-FolT2$_{ATP}$ reconstituted into lipid nanodiscs. a, FSC plot of the final reconstruction with the arrow indicating the global resolution based on the threshold of FSC = 0.143. **b**, Anisotropy plot of the final reconstruction as determined using the remote 3DFSC processing server[1]. The global FSC curve is represented in yellow. The directional FSCs along the x, y and z axes are displayed in blue, green and pink, respectively. The sphericity value reported by 3DFSC was 0.972 out of 1. **c**, Angular distribution plot of particles included in the asymmetric 3D reconstruction. The number of particles with their respective orientation is represented by length and colour of the cylinders. **d**, Final reconstructed map coloured according to the local resolution estimated in cryoSPARC[2]. **e**, Selected densities (semi-transparent surface renderings) for indicated residue ranges are shown in relation to the fitted atomic model (shown in stick representation). The surface renderings of the selected regions are coloured according to the local resolution estimated.

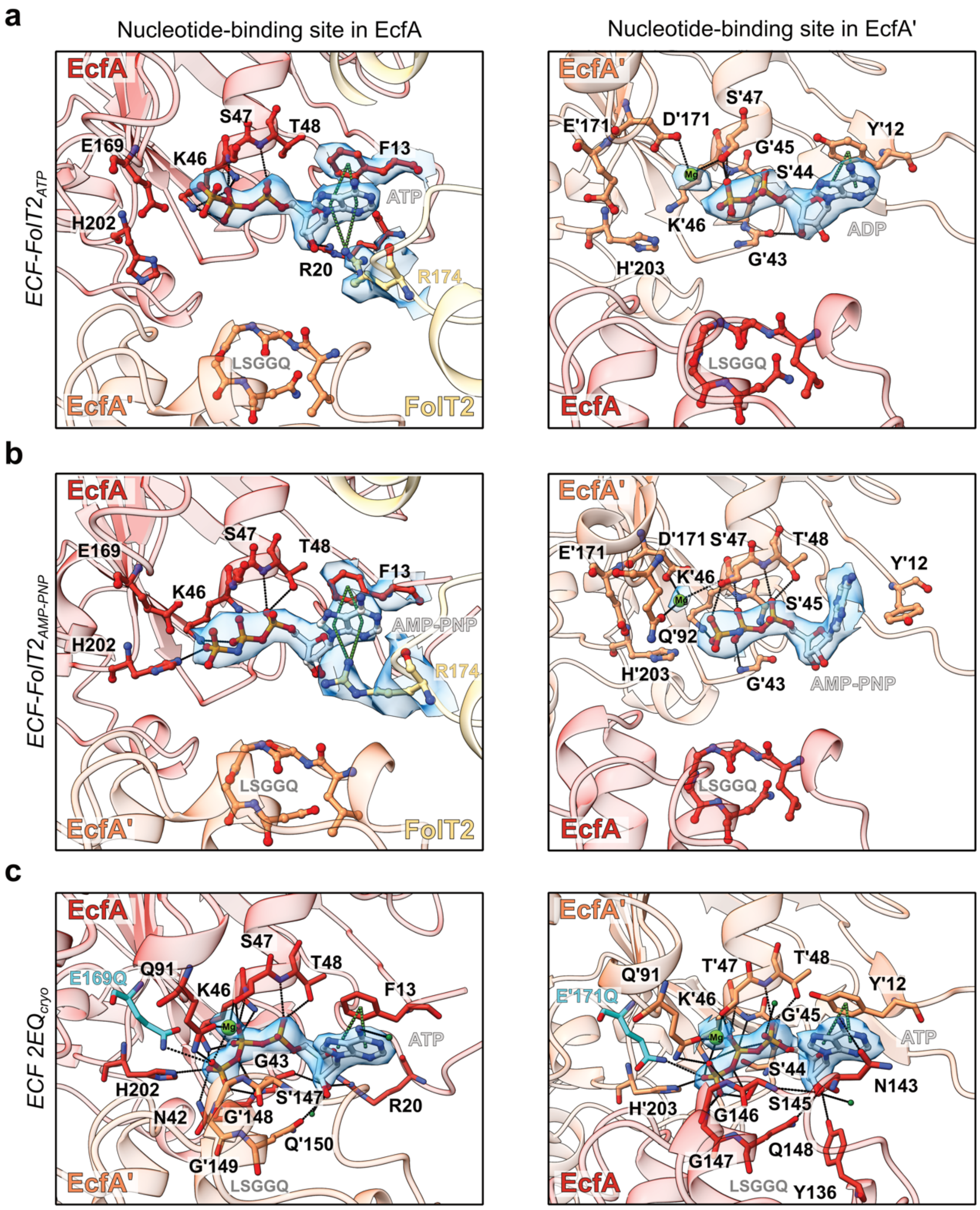

Supplementary Fig. 3 – Nucleotide binding to the ATPase subunits EcfA and EcfA'. a-c, Close-up views of the nucleotide-binding sites in EcfA (left) and EcfA' (right) of ATPase open ECF-FolT2$_{ATP}$ (**a**), ECF-FolT2$_{AMPPNP}$ (**b**), and of the mutant solitary ECF module 2EQ$_{cryo}$ with closed ATPases (**c**). Structures are shown in cartoon representation with key residues labelled, displayed in stick representation and coloured according to the individual subunits with the exception of the (teal) glutamine residues that substitute the conserved glutamates in the Walker B motifs in the mutant solitary ECF module 2EQ$_{cryo}$ (**c**). Nucleotides are shown in stick representation. Modelled magnesium ions and water molecules are shown as light and dark green spheres, respectively. Corresponding cryo-EM densities are shown for all bound nucleotides and magnesium ions, and for F13$_{EcfA}$ and R174$_{FolT2}$ in both ECF-FolT2 structures. Hydrogen bonds and π-interactions are shown as thin black and thick green dotted lines. The individual subunits/regions are coloured as in Fig. 1.

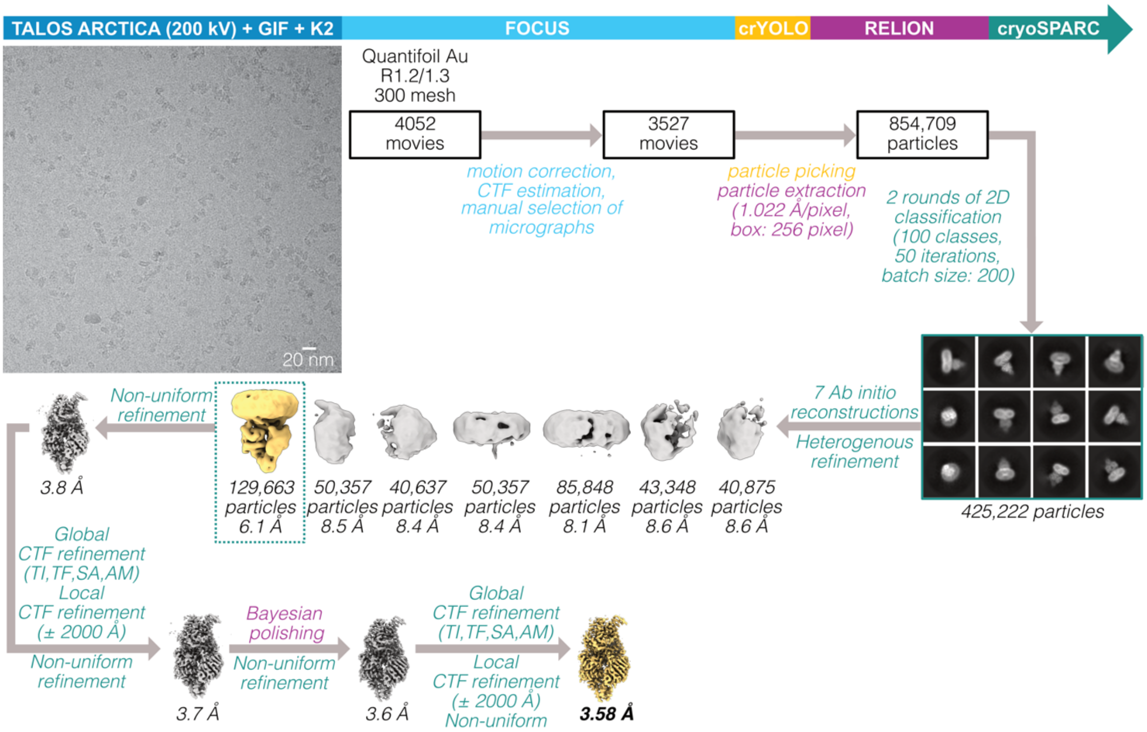

Supplementary Fig. 4 - Cryo-EM reconstruction of ECF-FolT2$_{AMPPNP}$ reconstituted into lipid nanodiscs. Detailed image processing workflow including a representative micrograph at a defocus of volume of -1.4 µm showing the particle distribution of ECF-FolT2$_{AMPPNP}$-MSP2N2 in the presence of 2.9 mM Fos-choline 8 on a Quantifoil Au R1.2/1.3 300 mesh grid. Abbreviations correspond to following: TI = Tilt, TF = Trefoil, SA = Spherical Aberration, AM = Anisotropic magnification.

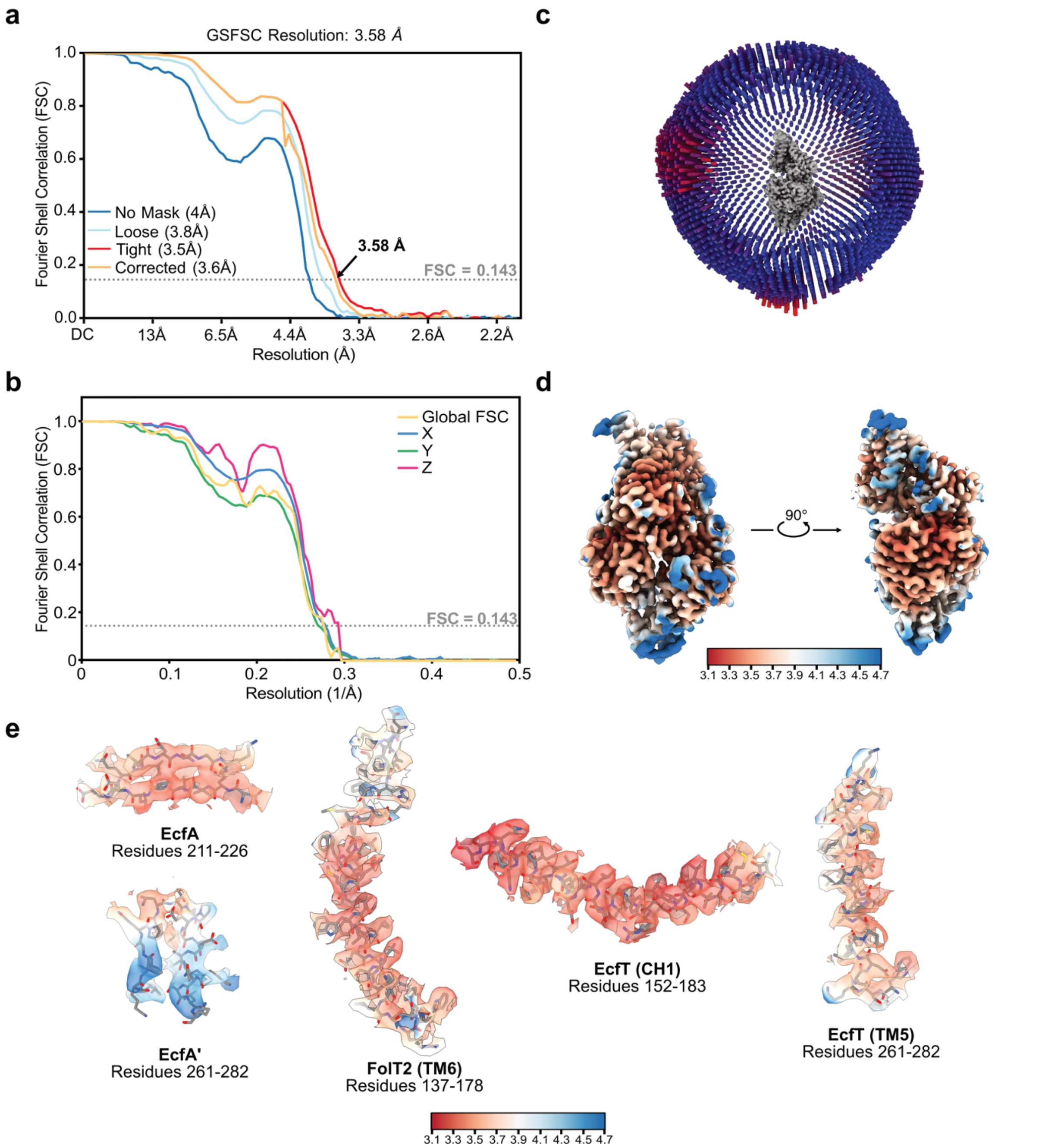

Supplementary Fig. 5 – Validation of the cryo-EM reconstruction of ECF-FolT2AMP-PNP reconstituted into lipid nanodiscs. a, FSC plot of the final reconstruction with the arrow indicating the global resolution based on the threshold of FSC = 0.143. **b**, Anisotropy plot of the final reconstruction as determined using the remote 3DFSC processing server[1]. The global FSC curve is represented in yellow. The directional FSCs along the x, y and z axes are displayed in blue, green and pink, respectively. The sphericity value reported by 3DFSC was 0.989 out of 1. **c**, Angular distribution plot of particles included in the asymmetric 3D reconstruction. The number of particles with their respective orientation is represented by length and colour of the cylinders. **d**, Final reconstructed map coloured according to the local resolution estimated in cryoSPARC[2]. **e**, Selected densities (semi-transparent surface renderings) for indicated residue ranges are shown in relation to the fitted atomic model (shown in stick representation). The surface renderings of the selected regions are coloured according to the local resolution estimated.

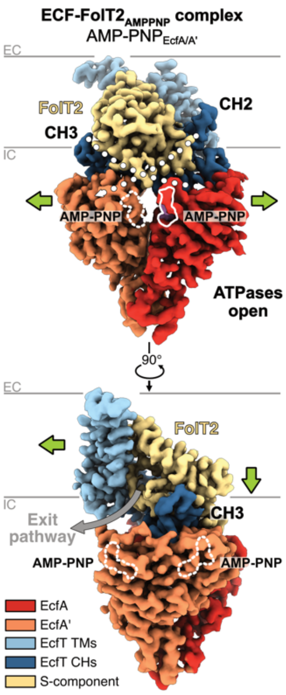

Supplementary Fig. 6 – Cryo-EM map of the AMP-PNP-bound ECF-FolT2$_{\text{AMPPNP}}$ complex. View from the membrane plane onto the FolT2 (top) and rotated by 90° onto the site of the exit pathway (grey arrow) for folate from the cavity in FolT2. The shape of the coupling helices (CH2 and CH3) is indicated by white dotted lines with the terminal triangle indicating the conserved 'X-Arg-X' anchor motifs. Bound AMP-PNP molecules are indicated by white contours (solid lines - located towards the front, dashed lines - located towards the back) and labels. The green arrows indicate the direction in which the structural elements have relocated when compared to panel **Fig 1c**. The approximate boundaries (grey lines) of the membrane and the extracellular (EC) and intracellular (IC) sides are indicated. The individual subunits/regions are coloured as in Fig. 1.

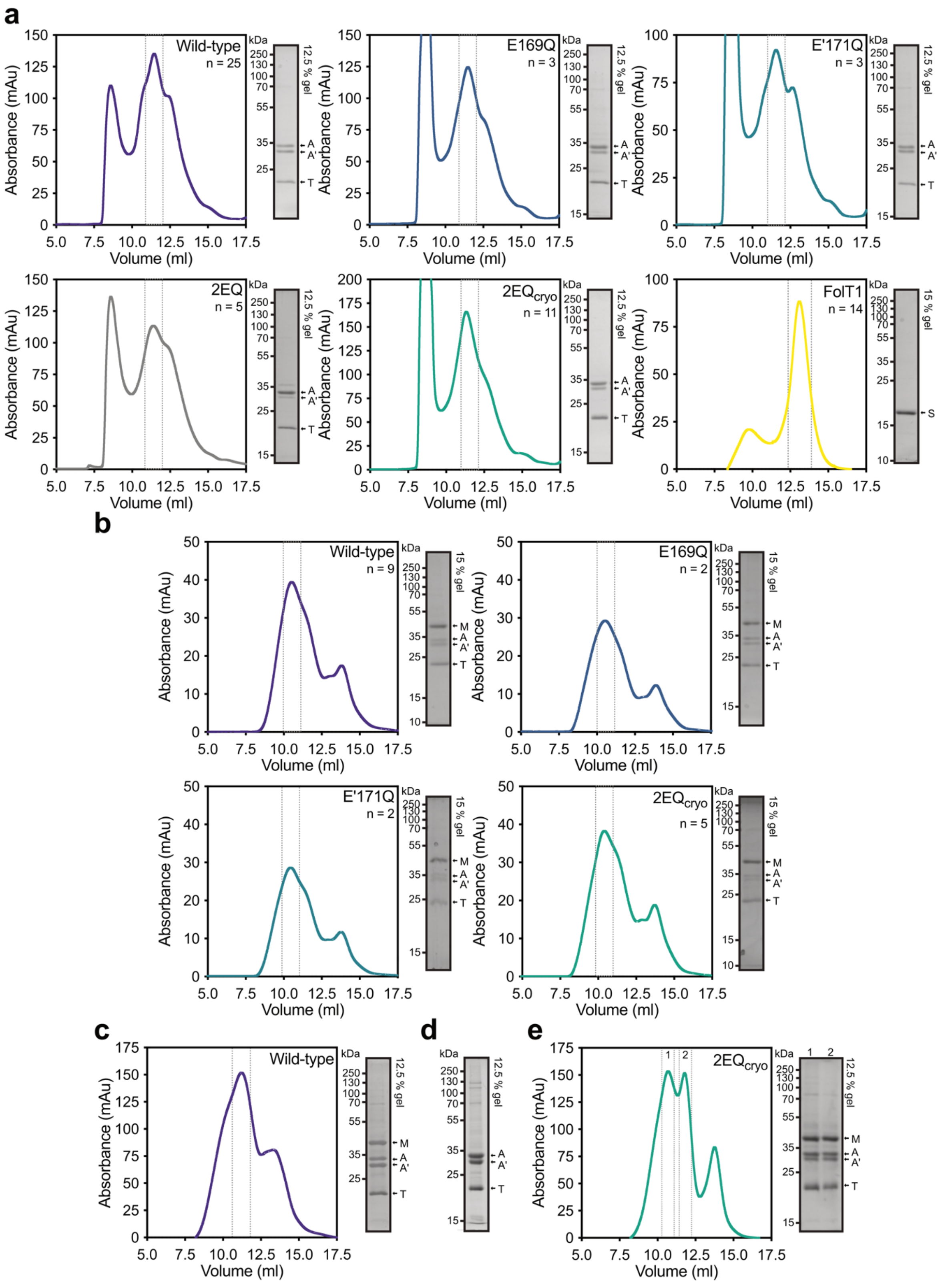

Supplementary Fig. 7 – Purification of solitary ECF module variants and FolT1. **a-e**, Representative elution profiles from a Superdex 200 Increase 10/300 GL size-exclusion chromatography column. Pooled elution fractions are indicated by grey dotted lines across the peak fraction. Final samples after size-exclusion chromatography were separated on home-made 12.5% (w/v) or 15% (w/v) SDS-PAGE gels. Molecular weight (kDa) markers and the bands corresponding to the individual proteins in the sample are indicated (A for EcfA, A' for EcfA', T for EcfT, and M for MSP2N2). **a**, Solitary ECF module variants and FolT1 in DDM micelles. **b**, Solitary ECF

module variants reconstituted into MSP2N2 lipid nanodiscs. **c-e**, The final samples used for cryo-EM grid vitrification of the wild-type solitary ECF module embedded in MSP2N2 lipid nanodiscs (**c**) or DDM detergent micelles (**d**), and of the mutant solitary ECF module $2EQ_{cryo}$ embedded in lipid nanodiscs (**e**). The ‘n’ numbers reflect the number of each experiment repeated independently with similar results. The original uncropped SDS-PAGE gel images are provided as a Source Data file.

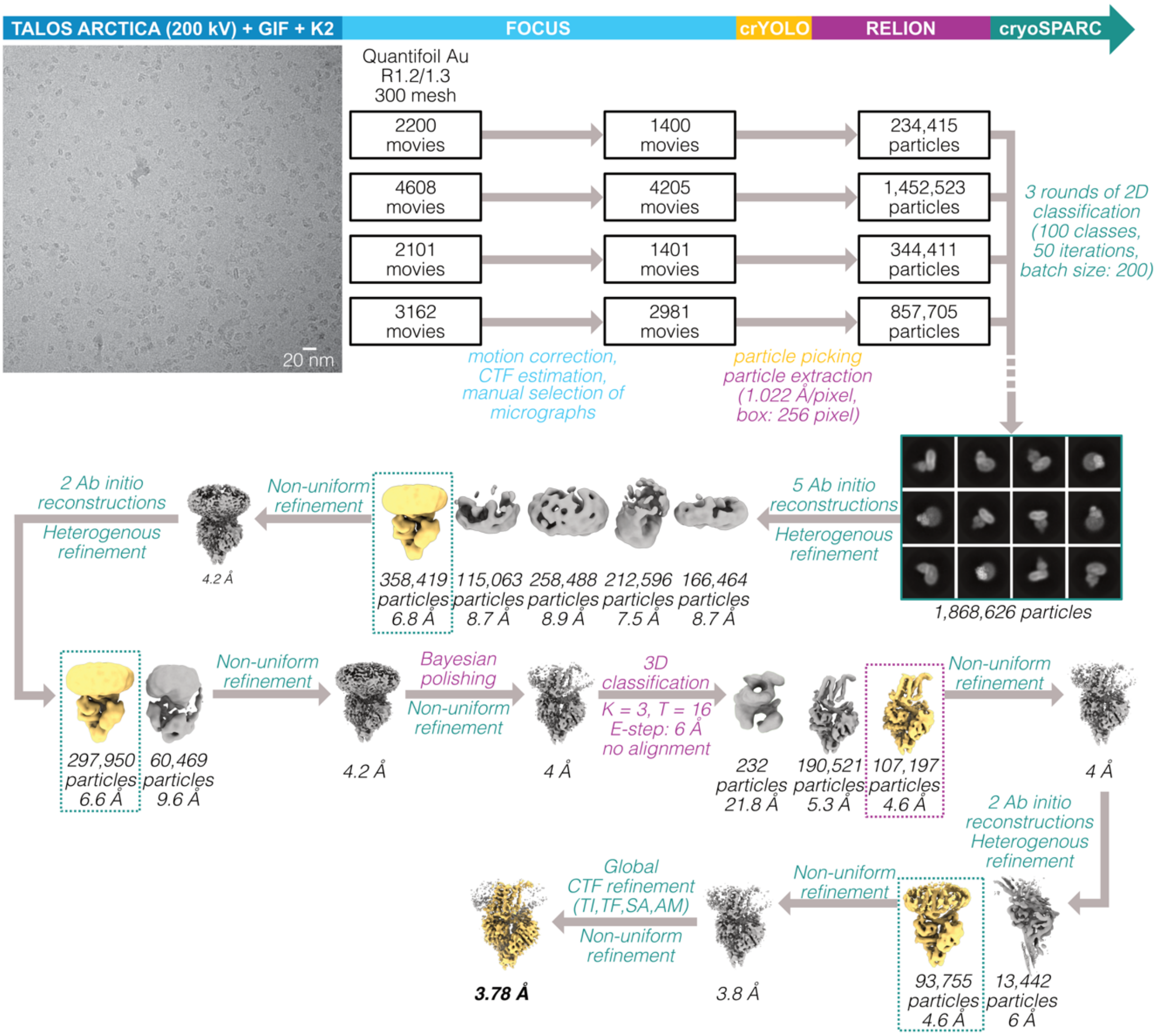

Supplementary Fig. 8 - Cryo-EM reconstruction of the wild-type solitary ECF module reconstituted into lipid nanodiscs. Detailed image processing workflow including a representative micrograph at a defocus of volume of -1.4 µm showing the particle distribution of wild-type solitary ECF module-MSP2N2 in the presence of 2.9 mM Fos-choline 8 on a Quantifoil Au R1.2/1.3 300 mesh grid. Abbreviations correspond to following: K = classes, T = tau_fudge, TI = Tilt, TF = Trefoil, SA = Spherical Aberration, AM = Anisotropic magnification.

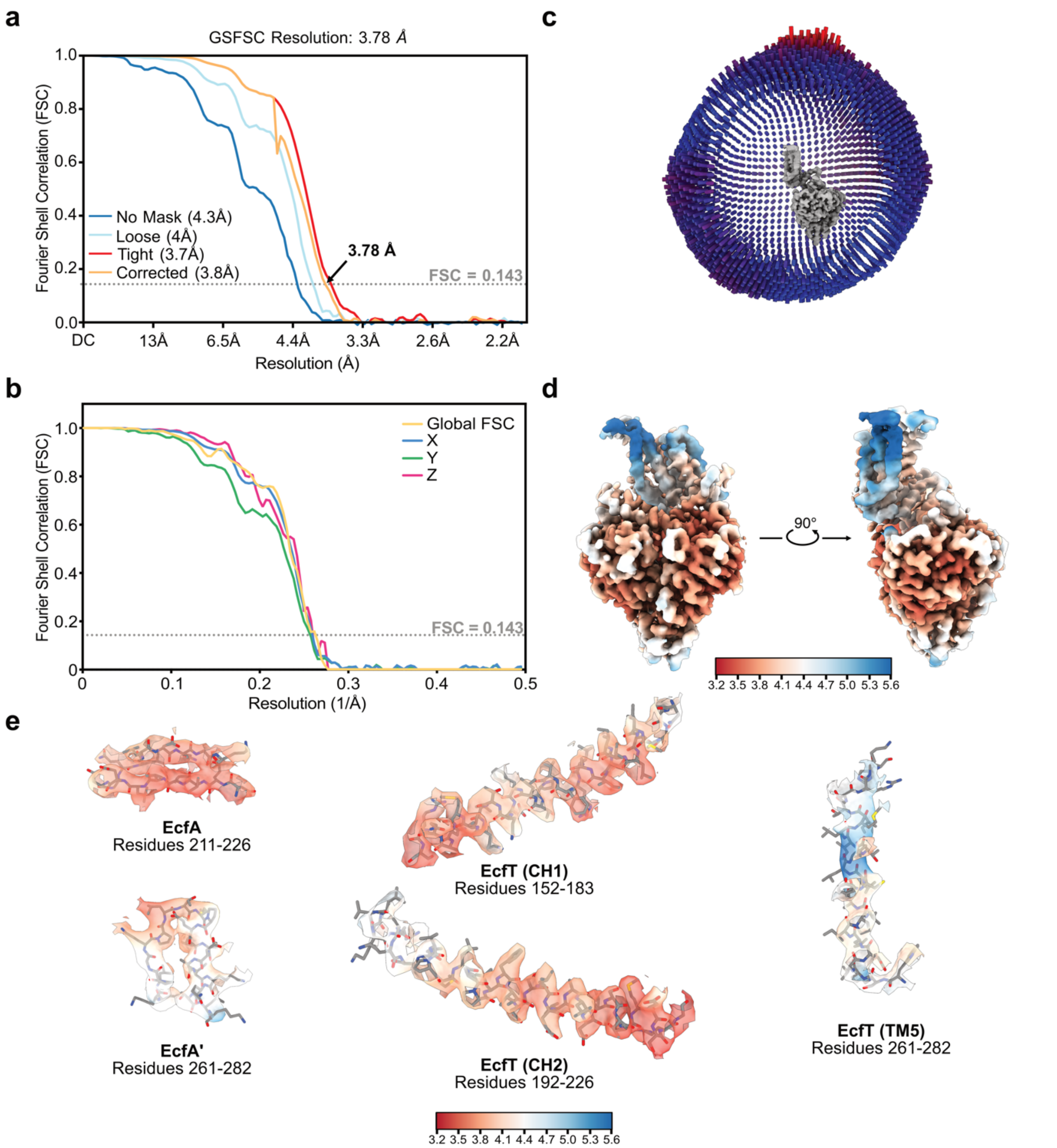

Supplementary Fig. 9 – Validation of the wild-type solitary ECF module reconstituted into lipid nanodiscs. a, FSC plot of the final reconstruction with the arrow indicating the global resolution based on the threshold of FSC = 0.143. **b**, Anisotropy plot of the final reconstruction as determined using the remote 3DFSC processing server[1]. The global FSC curve is represented in yellow. The directional FSCs along the x, y and z axes are displayed in blue, green and pink, respectively. The sphericity value reported by 3DFSC was 0.975 out of 1. **c**, Angular distribution plot of particles included in the asymmetric 3D reconstruction. The number of particles with their respective orientation is represented by length and colour of the cylinders. **d**, Final reconstructed map coloured according to the local resolution estimated in cryoSPARC[2]. **e**, Selected densities (semi-transparent surface renderings) for indicated residue ranges are shown in relation to the fitted atomic model (shown in stick representation). The surface renderings of the selected regions are coloured according to the local resolution estimated.

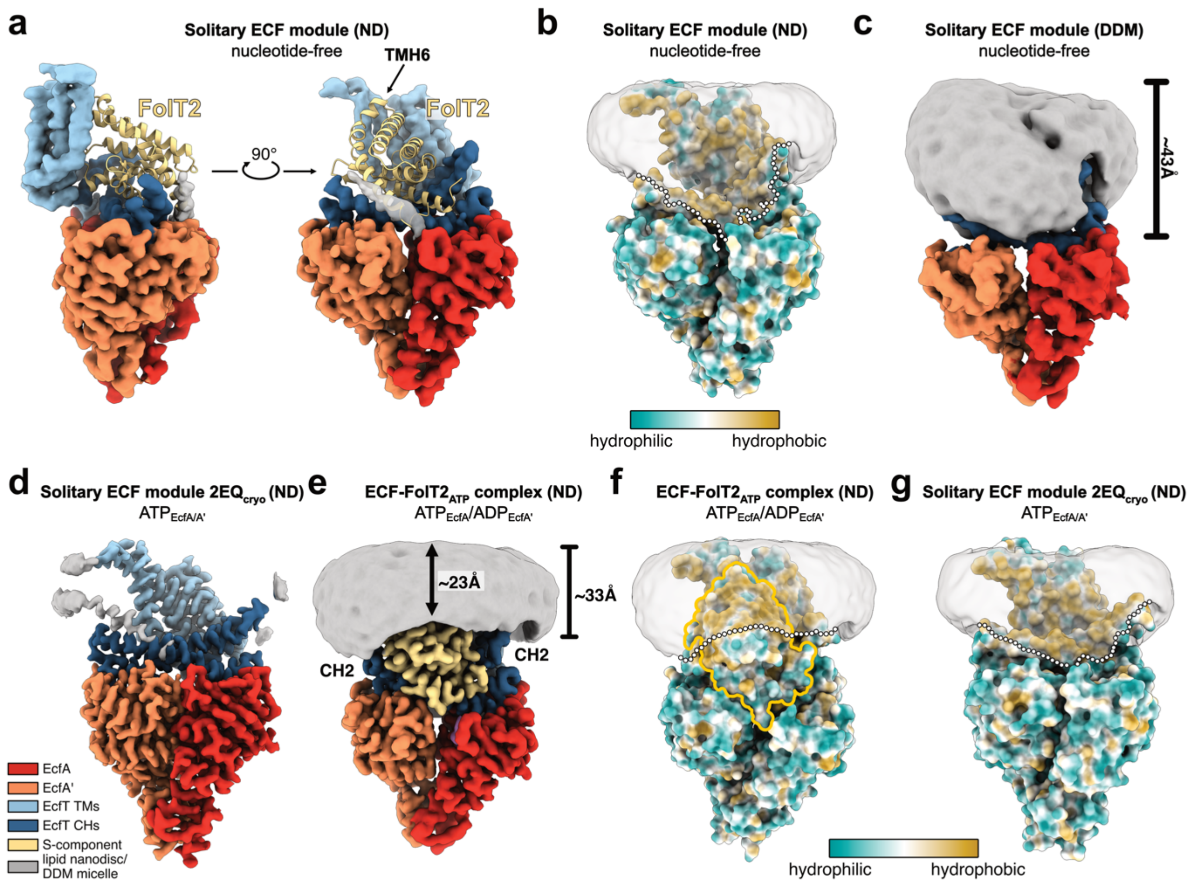

Supplementary Fig. 10 – Structural profiles of the surrounding environment observed in the cryo-EM maps. a, Cryo-EM map of the wild-type solitary ECF module viewed from the membrane to highlight the retained docking site for the S-component and an observed density (grey) between the ATPase dimer. A model of FolT2 from ECF-FolT2 was docked into the vacant docking site. The cryo-EM map was contoured at 5 σ. **b**, Model of the wild-type solitary ECF module viewed from the membrane plane is shown in surface representation and coloured by hydrophobicity. **c**, Cryo-EM map of the wild-type solitary ECF module in detergent micelles. The approximate thickness of the detergent micelle (labelled) was determined using Chimera X[3] and indicated with a black bar. The detergent micelle density (grey) was obtained from an unsharpened map lowpass filtered to 6 Å and contoured at 8 σ. **d**, Cryo-EM map of the mutant solitary ECF module 2EQ$_{cryo}$ view from the membrane plane to highlight an observed density (grey) between the ATPase dimer. The cryo-EM map was contoured at 7.5 σ. **e,** Cryo-EM map of the nucleotide-bound ECF-FolT2$_{ATP}$ complex viewed from the membrane plane. The approximate nanodisc thickness (labelled) in various regions was determined using Chimera X and indicated with black arrows and bars. The nanodisc density (grey) was obtained from an unsharpened map lowpass filtered to 6 Å and contoured at 3 σ. **f-g**, Models of the ECF-FolT2$_{ATP}$ complex (**f**) and mutant solitary ECF module 2EQ$_{cryo}$ (**g**) viewed from the membrane plane are shown in surface representation and coloured by hydrophobicity. FolT2 bound in the ECF-FolT2$_{ATP}$ complex is highlighted with a yellow contour (**f**). **a-d**, The subunits/regions are coloured as in Fig. 1. **b,f,g**, Nanodisc cryo-EM densities are shown in grey and made transparent. Hydrophobic surface boundaries are indicated as white dotted lines.

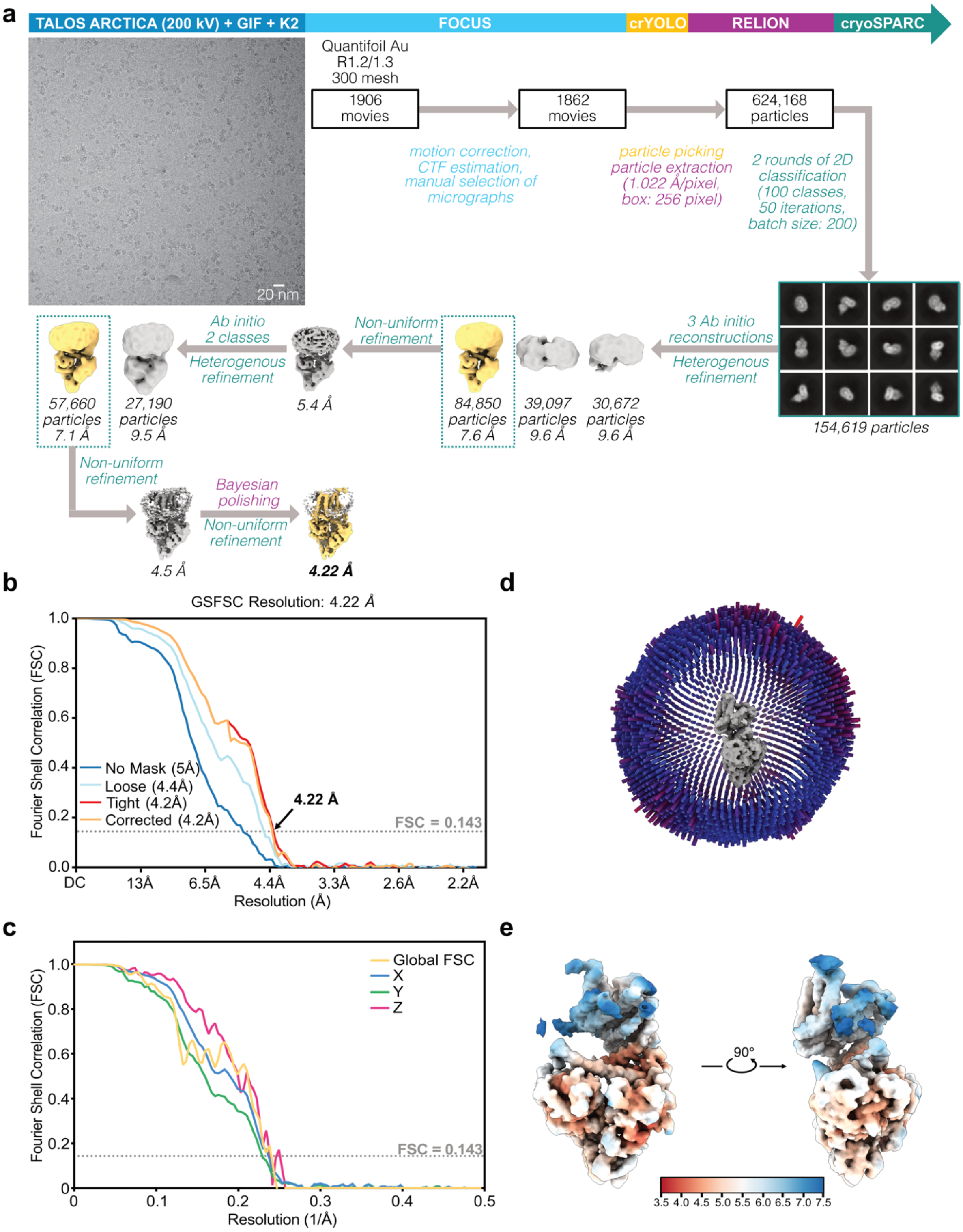

Supplementary Fig. 11 - Cryo-EM reconstruction and validation of the wild-type ECF module in DDM micelles. a, Detailed image processing workflow including a representative micrograph at a defocus of volume of -1.4 µm showing the particle distribution of the wild-type ECF module-DDM in the presence of 2.9 mM Fos-choline 8 on a Quantifoil Au R1.2/1.3 300 mesh grid. **b**, FSC plot of the final reconstruction with the arrow indicating the global resolution based on the threshold of FSC = 0.143. **c**, Anisotropy plot of the final reconstruction as determined using the remote 3DFSC processing server[1]. The global FSC curve is represented in yellow. The directional FSCs along the x, y and z axes are displayed in blue, green and pink, respectively. The sphericity value reported by 3DFSC was 0.754 out of 1. **d**, Angular distribution plot

of particles included in the asymmetric 3D reconstruction. The number of particles with their respective orientation is represented by length and colour of the cylinders. **e**, Final reconstructed map coloured according to the local resolution estimated in cryoSPARC[2].

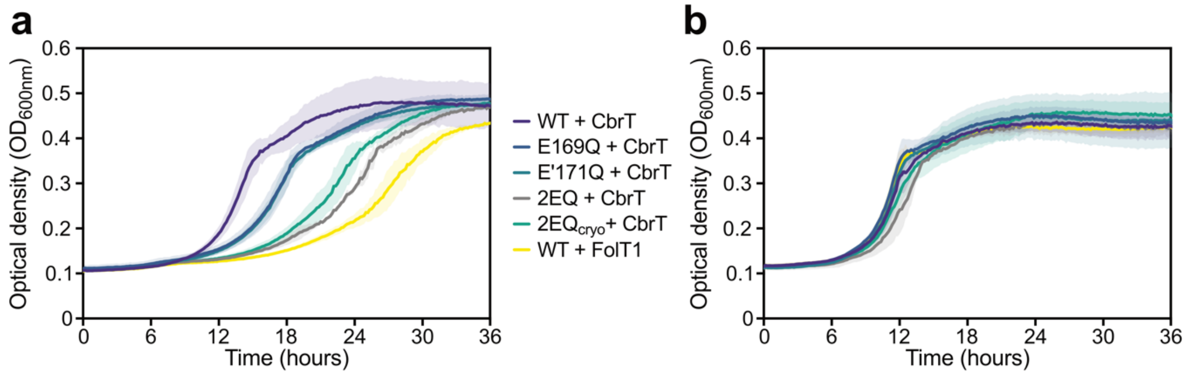

Supplementary Fig. 12 – Activity of the ECF module variants using a cobalamin-dependent growth assay. a, Growth assay in M9 minimal medium supplemented with 1 nM cobalamin using the *E. coli* ΔFEC strain co-expressing the wild-type ECF module + CbrT (purple), mutant ECF module E169Q + CbrT (blue), mutant ECF module E’171Q + CbrT (teal), mutant ECF module 2EQ + CbrT (grey), mutant ECF module $2EQ_{cryo}$ + CbrT (green) and wild-type ECF module + FolT1 (yellow). **b**, Growth assay similar to panel **a**, using M9 medium supplemented with 50 µg ml^{-1} methionine as positive control. **a-b**, Data was obtained from three biological repeats, each containing three technical repeats. The coloured areas indicate the standard deviation of averaged biological replicates. Source data are provided as a Source Data file.

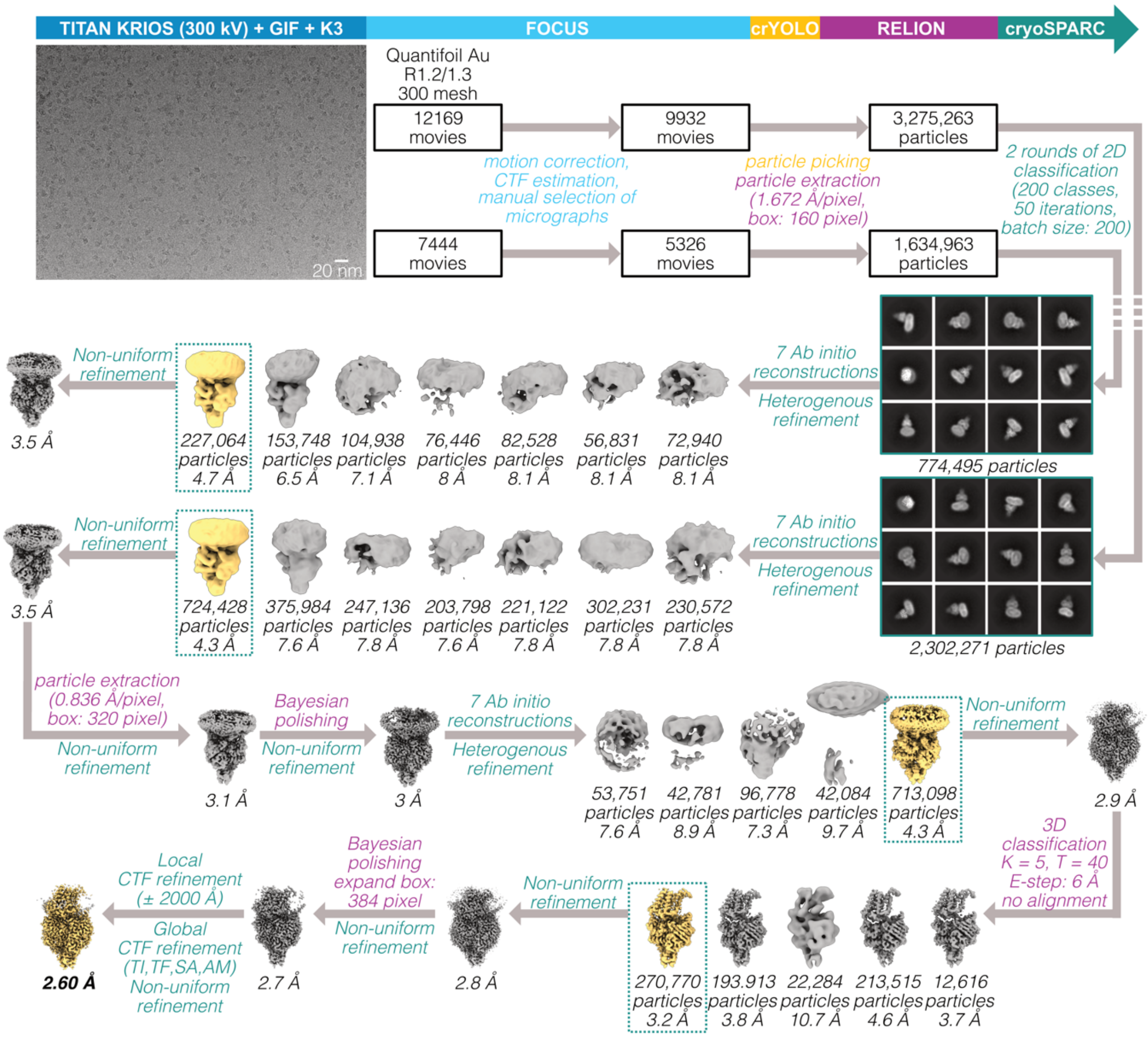

Supplementary Fig. 13 - Cryo-EM reconstruction of the mutant solitary ECF module 2EQ$_{cryo}$ reconstituted into lipid nanodiscs. Detailed image processing workflow including a representative micrograph at a defocus of volume of -1.4 µm showing the particle distribution of mutant solitary ECF module 2EQ$_{cryo}$-MSP2N2 in the presence of 2.9 mM Fos-choline 8 on a Quantifoil Au R1.2/1.3 300 mesh grid. Abbreviations correspond to following: K = classes, T = tau_fudge, TI = Tilt, TF = Trefoil, SA = Spherical Aberration, AM = Anisotropic magnification.

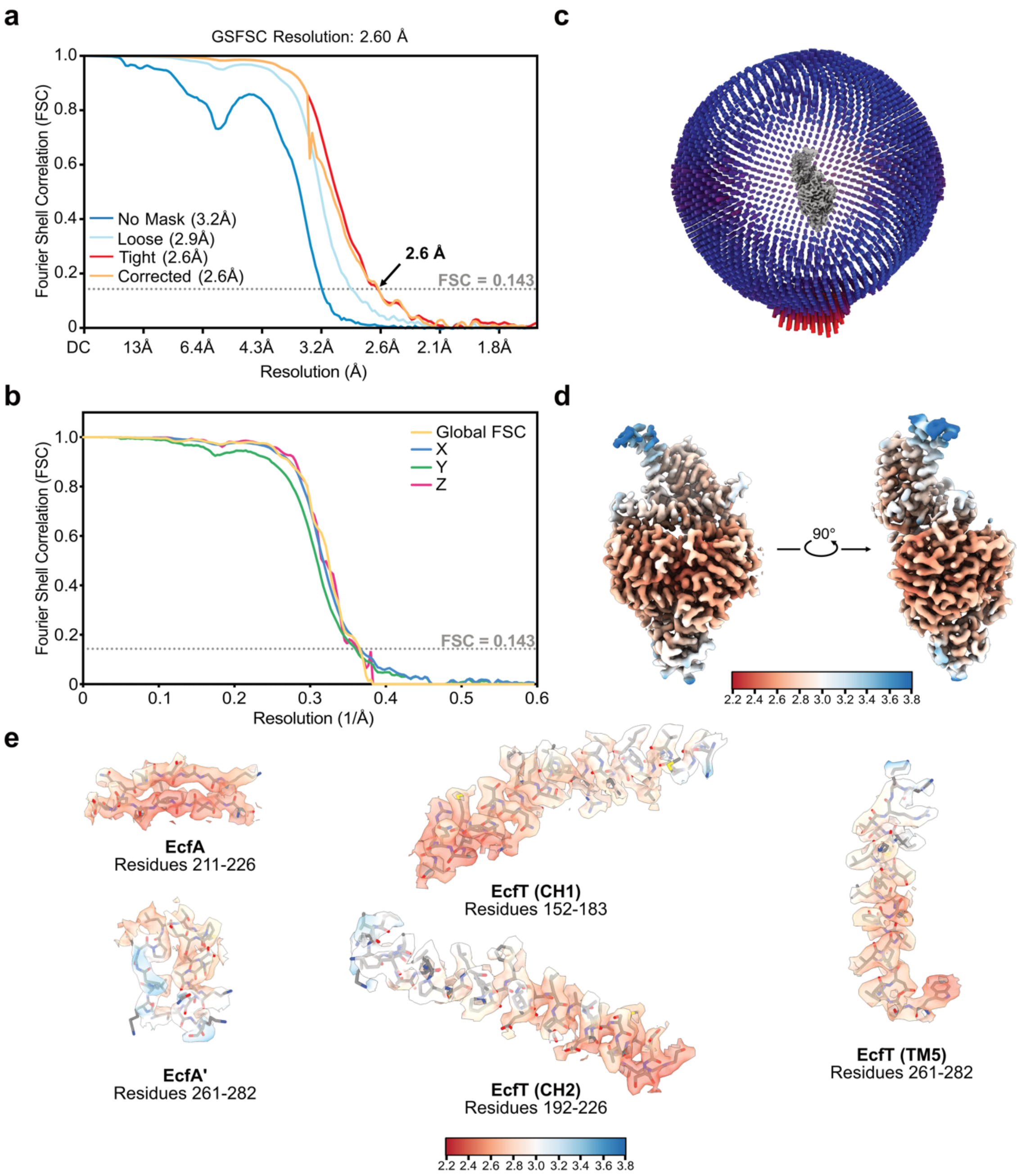

Supplementary Fig. 14 – Validation of the cryo-EM reconstruction of the mutant solitary ECF module 2EQ$_{cryo}$ reconstituted into lipid nanodiscs. a, FSC plot of the final reconstruction with the arrow indicating the global resolution based on the threshold of FSC = 0.143. **b**, Anisotropy plot of the final reconstruction as determined using the remote 3DFSC processing server[1]. The global FSC curve is represented in yellow. The directional FSCs along the x, y and z axes are displayed in blue, green and pink, respectively. The sphericity value reported by 3DFSC was 0.981 out of 1. **c**, Angular distribution plot of particles included in the asymmetric 3D reconstruction. The number of particles with their respective orientation is represented by length and colour of the cylinders. **d**, Final reconstructed map coloured according to the local resolution estimated in cryoSPARC[2]. **e**, Selected densities (semi-transparent surface renderings) for indicated residue ranges are shown in relation to the fitted atomic model (shown in stick representation). The surface renderings of the selected regions are coloured according to the local resolution estimated.

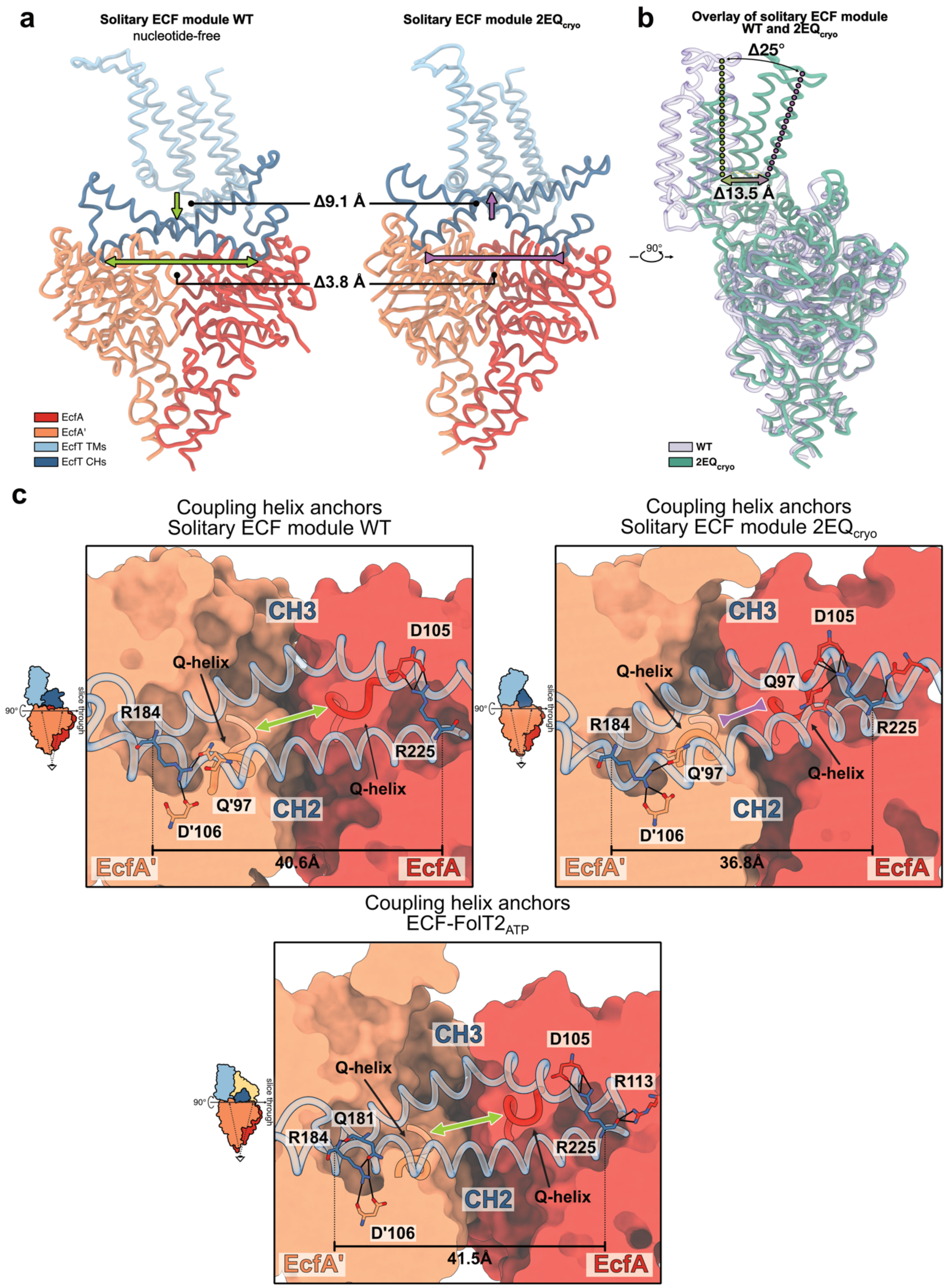

Supplementary Fig. 15 – Movement of structural elements induced by binding of Mg-ATP. a, Model of the wild-type solitary ECF module (left) with open ATPases and the mutant solitary ECF module 2EQ$_{cryo}$ with closed ATPases (right) viewed from the membrane plane are shown in cartoon representation. Arrows highlight (green, purple) the direction and numbers indicate the distance difference of movements of structural elements. **b**, Models of the wild-type solitary ECF module (purple) and mutant solitary ECF module 2EQ$_{cryo}$ (green), viewed from the membrane plane and rotated by 90°

relative to the models shown in panel **a**, are superimposed to highlight the translation and rotation movements of the EcfT transmembrane domain upon Mg-ATP binding. Arrows highlight (gradient from green, purple) the direction and numbers indicate the distance or angle difference of movements of structural elements. **c**, Cross sections of the ATPase dimer as viewed from the cytoplasm of the wild-type solitary ECF module (left), the mutant solitary ECF module 2EQ$_{cryo}$ (right), and the ECF-FolT2$_{ATP}$ complex (bottom) highlight the anchors of the coupling helices (cartoon representation). A schematic representation in the box indicate the position of the slice through and the zoomed in region of the cross section. The conserved arginine residues found at the C-terminal ends of both coupling helices along with interacting residues are shown as sticks and labelled. The Q-helices are indicated and shown in cartoon representation. The arrows indicate the direction of movements of the ATPase dimer from the open to closed conformation (purple) and vice versa (green). **a,c**, The individual subunits/regions are coloured as in Fig. 1.

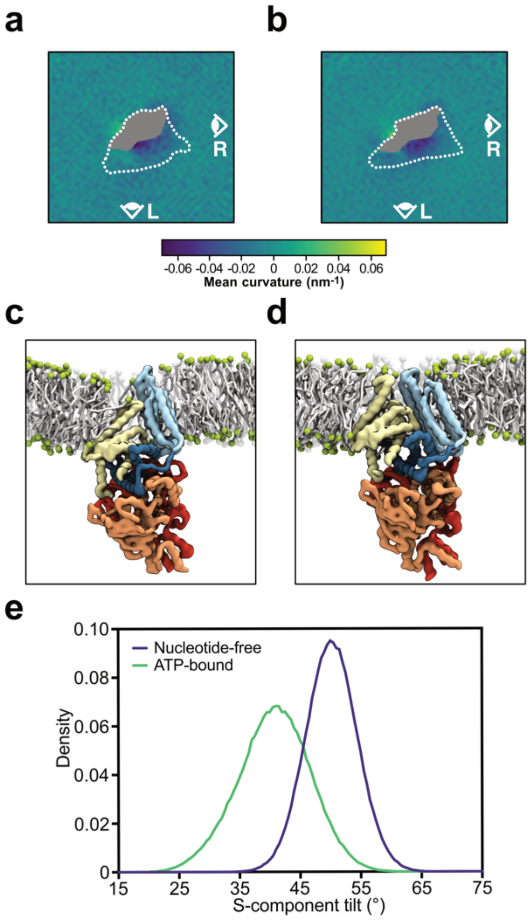

Supplementary Fig. 16 – Additional results from coarse-grained MD simulations. a-b, Curvature maps around both wild-type (**a**) and mutant (**b**) solitary ECF modules. Outline represents the boundaries of the EcfT subunit with the region of transmembrane domain filled in grey. Viewpoint L and R refer to the corresponding panels in Fig. 3. **c-d**, Snapshots from simulations with ECF-FolT2 in nucleotide-free (**c**) and ATP-bound (**d**) states inserted in a bacterial model membrane composed of POPE : POPG : cardiolipin. The individual subunits/regions are coloured as in Fig. 1. Lipid are coloured as in Fig. 3. (**e**) Tilt angle distributions in relation to the membrane normal of the S-component in simulations of the ECF-FolT2 complex in nucleotide-free ATPase open (purple) and ATP-bound ATPase closed (green) states.

Supplementary Table 1 – DNA oligonucleotides sequences of the PCR primers to introduce glutamate-to-glutamine substitutions in EcfA and EcfA'.

**Primer**	**DNA sequence 5' to 3'**
EcfA	
E169Q-F1	AGCCCCAGGTCATCATCCTGGATCAGTCG
E169Q-R1	CGGGTCCAGCATGGAGGTCGACTGATC
EcfA'	
E171Q-F1	GCCTATGAGCCGGAGATCATCTGTTTAGATCAGCCG
E171Q-R1	TCCAGGCCAGCTGCCGGCTGATC

Supplementary Table 2 - Data collection, processing and refinement statistics

	**ECF-FolT2$_{ATP}$** in MSP2N2 lipid nanodiscs **(EMD-16120, PDB 8BMP, EMPIAR 11307)**	**ECF-FolT2$_{AMPPNP}$** in MSP2N2 lipid nanodiscs **(EMD-16121, PDB 8BMQ, EMPIAR 11308)**	**Solitary wild-type ECF module** in MSP2N2 lipid nanodiscs **(EMD-16122, PDB 8BMR, EMPIAR 11309)**	**Solitary wild-type ECF module** in DDM micelles **(EMD-16123, EMPIAR 11310)**	**Solitary ECF module 2EQ$_{cryo}$** in MSP2N2 lipid nanodiscs **(EMD-16124, PDB 8BMS, EMPIAR 113011)**
**Data collection and processing**					
Microscope	Thermo Fisher Talos Arctica				Thermo Fisher Titan Krios G1 (Krios 1)
Energy filter	Gatan GIF Bioquantum LS				Gatan Bioquantum
Detector	Gatan K2 Summit				Gatan K3 Bioquantum
Camera mode	counting mode				Counted Super Resolution Bin 2
Voltage (kV)	200				300
Nominal magnification	130,000x				105,000x
Pixel size (Å)	1.022				0.836
Exposure navigation	Stage shift (EPU) or Beam-image shift with a 3 x 3 pattern (Serial EM)				Aberration Free Image Shift (AFIS)
Number of frames	60				75
Total electron exposure ($e^-/Å^2$)	50.1				60
Defocus range (μm)	-0.5 to -2.0				-0.9 to -1.9
Data collection software	EPU v2.7.0 / Serial EM v3.8.0 beta	Serial EM v3.8.0 beta	Serial EM v3.9.0 beta	Serial EM v3.9.0 beta	EPU v2.8.1
Micrographs collected	502 + 597 / 2187 (total 3286)	4052	2200 + 4608 + 2101 + 3162 (total 12071)	1906	12169 + 7444 (total 19613)
Micrographs used	498 + 487 / 2006 (total 2991)	3527	1400 + 4205 + 1401 + 2981 (total 9987)	1862	9932 + 5326 (total 15,258)
Total number of extracted particles	1,207,115	854,709	2,889,054	154,619	3,275,263 + 1,634,963 (total 4,910,226)
Final number of particles	167,227	129,663	93,755	54,660	270,770
Symmetry imposed	C1	C1	C1	C1	C1
Global map resolution (Å)*	3.22	3.58	3.78	4.25	2.6
Map resolution range (Å)	~2.8 – ~4.6	~3.1 to ~4.7	~3.2 to ~5.6	~3.5 to ~7.5	~2.2 to ~3.8
FSC threshold	0.143	0.143	0.143	0.143	0.143
**Refinement**					
Initial model used (PDB code)	7NNU	7NNU	7NNU (Chains A + B + D)	-	8BMR (This work)
Model resolution	3.3	3.7	4.0	-	2.8
FSC threshold	0.5	0.5	0.5	-	0.5
Map sharpening B-factor ($Å^2$)	-113	-118	-148	-	-90
Model composition					
Chains	4	4	3	-	4
Non-hydrogen atoms	7794	7784	6330	-	6570
Protein residues	989	987	808	-	822
Water	-	-	-	-	72
Ligands					
Mg	1	1	-	-	2
ATP	1	-	-	-	2
ADP	1	-	-	-	-
ANP	-	2	-	-	-
B factors ($Å^2$)					
Protein (min/max/mean)	2.06/159.76/62.98	8.03/94.61/27.03	22.52/236.79/81.32	-	3.56/157.16/56.75
Water (min/max/mean)	-	-	-	-	20.45/72.09/41.65
Ligand (min/max/mean)	55.08/90.25/70.17	23.14/40.61/35.75	-	-	8.63/37.59/24.13
R.m.s. deviation					
Bond lengths (Å)	0.003	0.005	0.003	-	0.003
Bond angles (°)	0.636	0.617	0.610	-	0.521
Validation					
Molprobity score	1.42	1.78	1.78	-	1.38
Clashscore	6.03	7.25	7.90	-	6.04
EMRinger score	2.97	2.32	1.29	-	3.85
Ramachandran plot					
Favoured (%)	97.55	94.46	94.99	-	97.79
Allowed (%)	3.03	5.54	5.01	-	2.21
Outliers (%)	0	0	0	-	0
Rotamer outliers (%)	0	0	0	-	0
Rama-Z					
Whole	0.66 (0.27) (N = 979)	-0.06 (0.27) (N = 975)	-0.04 (0.31) (N = 798)	-	0.83 (0.29) (N.= 814)
Helix	1.09 (0.22) (N = 571)	0.76 (0.23) (N = 547)	0.67 (0.26) (N = 414)	-	1.56 (0.25) (N = 452)
Sheet	0.87 (0.71) (N = 62)	-0.74 (0.60) (N = 70)	0.19 (0.69) (N = 68)	-	-0.80 (0.52) (N = 74)
Loop	-0.66 (0.32) (N = 346)	-1.06 (0.33) (N = 358)	-0.94 (0.36) (N = 316)	-	-0.66 (0.35) (N = 288)

Supplementary References

1. Tan, Y. Z. *et al.* Addressing preferred specimen orientation in single-particle cryo-EM through tilting. *Nat Methods* **14**, 793–796 (2017).
2. Punjani, A., Rubinstein, J. L., Fleet, D. J. & Brubaker, M. A. cryoSPARC: algorithms for rapid unsupervised cryo-EM structure determination. *Nature Methods* (2017) doi:10.1038/nmeth.4169.
3. Goddard, T. D. *et al.* UCSF ChimeraX: Meeting modern challenges in visualization and analysis: UCSF ChimeraX Visualization System. *Protein Science* **27**, 14–25 (2018).
