# Supplementary material for: Expulsion mechanism of the substrate-translocating subunit in ECF transporters
Source: Nat Commun. 2023 Jul 25;14:4484. doi: 10.1038/s41467-023-40266-1 (PMC10368641; doi:10.1038/s41467-023-40266-1)
Supplement: Supplementary file 3 — Description of Additional Supplementary Files [file 41467_2023_40266_MOESM3_ESM.pdf]

## Description of Additional Supplementary Files

**Supplementary Movie 1:** Conformational changes induced by ATP binding in ECF transporters. A morph between the open and close ATPase conformation of the solitary ECF module is shown with structures are shown in cartoon representation, and coloured according to Fig. 1.

**Supplementary Movie 2:** Mechanism of S-component expulsion from the ECF module. Structures of ECF-FolT2 in the inward-facing open and in the ATP-bound conformation, and of the ECF module in the ATP-bound S-component-released and in the open S-component-released conformation are shown. The structures are shown in cartoon representation with bound Mg-ATP molecules displayed as spheres, and coloured according to Fig. 1.
