# Supplementary material for: Expulsion mechanism of the substrate-translocating subunit in ECF transporters
Source: Nat Commun. 2023 Jul 25;14:4484. doi: 10.1038/s41467-023-40266-1 (PMC10368641; doi:10.1038/s41467-023-40266-1)
Supplement: Supplementary file 6 — Reporting Summary [file 41467_2023_40266_MOESM6_ESM.pdf]

nature portfolio

Corresponding author(s): Cristina Paulino, Dirk J. Slotboom

Last updated by author(s): Jul 11, 2023

Reporting Summary

Nature Portfolio wishes to improve the reproducibility of the work that we publish. This form provides structure for consistency and transparency in reporting. For further information on Nature Portfolio policies, see our Editorial Policies and the Editorial Policy Checklist.

Statistics

For all statistical analyses, confirm that the following items are present in the figure legend, table legend, main text, or Methods section.

n/a	Confirmed	
☐	☒	The exact sample size (n) for each experimental group/condition, given as a discrete number and unit of measurement
☐	☒	A statement on whether measurements were taken from distinct samples or whether the same sample was measured repeatedly
☒	☐	The statistical test(s) used AND whether they are one- or two-sided<br>*Only common tests should be described solely by name; describe more complex techniques in the Methods section.*
☒	☐	A description of all covariates tested
☒	☐	A description of any assumptions or corrections, such as tests of normality and adjustment for multiple comparisons
☐	☒	A full description of the statistical parameters including central tendency (e.g. means) or other basic estimates (e.g. regression coefficient) AND variation (e.g. standard deviation) or associated estimates of uncertainty (e.g. confidence intervals)
☒	☐	For null hypothesis testing, the test statistic (e.g. F, t, r) with confidence intervals, effect sizes, degrees of freedom and P value noted<br>*Give P values as exact values whenever suitable.*
☒	☐	For Bayesian analysis, information on the choice of priors and Markov chain Monte Carlo settings
☒	☐	For hierarchical and complex designs, identification of the appropriate level for tests and full reporting of outcomes
☒	☐	Estimates of effect sizes (e.g. Cohen's d, Pearson's r), indicating how they were calculated

Our web collection on statistics for biologists contains articles on many of the points above.

Software and code

Policy information about availability of computer code

Data collection	Cryo-EM data: EPU version 2.7.0 or 2.8.1 (Thermo Fisher Scientific), or Serial EM 3.8.0 beta or 3.9.0 beta. On-the-fly processing: FOCUS version 1.1.0 (managed through the SBGrid software manager version 2.5.6). Growth assay data: SoftMax Pro version 7.1.2 (Molecular Devices). ATPase assay data: SparksControl version 2.3 (Tecan). MD simulation data: GROMACS version 2020.7 using the Martini 3 coarse-grained model.
Data analysis	Cryo-EM data processing (managed through the SBGrid software manager version 2.5.6): MotionCor2 version 1.4.0, CTFFIND4.1.14, crYOLO version 1.7.5, 1.7.6 or 1.8.2, Relion 3.1.3, UCSF PyEM collection of Python scripts, DeepEMhancer. Other cryo-EM data processing software: cryoSPARC 3.2 or 3.3. Model building and refinement: COOT version 0.9.8.1, PHENIX version 1.20.1-4487. Cryo-EM map and model visualisation: ChimeraX version 1.3. MD simulation data analysis: Python version 3.8.10, MDAnalysis package version 2.2.0, IPython version 8.4.0, NumPy version 1.12.0, SciPy version 1.8.1, Scikit-Learn version 1.1.1, Voro++ version 0.4.6, Matplotlib version 3.5.2. MD simulation data visualisation and rendering: VMD version 1.9.4. Growth, uptake, and ATPase assay data analysis and visualisation: Prism version 9.3.1 (GraphPad Inc.).

For manuscripts utilizing custom algorithms or software that are central to the research but not yet described in published literature, software must be made available to editors and reviewers. We strongly encourage code deposition in a community repository (e.g. GitHub). See the Nature Portfolio guidelines for submitting code & software for further information.

Data

Policy information about availability of data

All manuscripts must include a data availability statement. This statement should provide the following information, where applicable:

- Accession codes, unique identifiers, or web links for publicly available datasets
- A description of any restrictions on data availability
- For clinical datasets or third party data, please ensure that the statement adheres to our policy

Cryo-EM density maps, half maps, and masks have been deposited in the Electron Microscopy Data Bank (EMDB) under accession numbers 16120 [https://www.ebi.ac.uk/emdb/EMD-16120] (ECF-FolT2ATP), 16121 [https://www.ebi.ac.uk/emdb/EMD-16121] (ECF-FolT2AMP-PNP), 16122 [https://www.ebi.ac.uk/emdb/EMD-16122] (ECF module WTMSP2N2), 16123 [https://www.ebi.ac.uk/emdb/EMD-16123] (ECF module WTDDM), and 16124 [https://www.ebi.ac.uk/emdb/EMD-16124] (ECF module 2EQcryo). Models are available through the Protein Data Bank (PDB) under the accession codes 8BMP [https://doi.org/10.2210/pdb8BMP/pdb] (ECF-FolT2ATP), 8BMQ [https://doi.org/10.2210/pdb8BMQ/pdb] (ECF-FolT2AMP-PNP), 8BMR [https://doi.org/10.2210/pdb8BMR/pdb] (ECF module WTMSP2N2), and 8BMS [https://doi.org/10.2210/pdb8BMS/pdb] (ECF module 2EQcryo). Raw movies have been deposited in the Electron Microscopy Public Image Archive (EMPIAR) under accession numbers 11307 [https://doi.org/10.6019/EMPIAR-11307] (ECF-FolT2ATP), 11308 [https://doi.org/10.6019/EMPIAR-11308] (ECF-FolT2AMP-PNP), 11309 [https://doi.org/10.6019/EMPIAR-11309] (ECF module WTMSP2N2), 11310 [https://doi.org/10.6019/EMPIAR-11310] (ECF module WTDDM), and 11311 [https://doi.org/10.6019/EMPIAR-11311] (ECF module 2EQcryo). The previously resolved structure of ECF-FolT2 in the apo state used in this study is available through the PDB under the accession code 7NNU [https://doi.org/10.2210/pdb7NNU/pdb]. DNA sequences used in this study are available through GenBank under the accession code CR954253.1 [https://www.ncbi.nlm.nih.gov/nucleotide/CR954253.1] with regions 360354 to 362831, 1399702 to 1400232, and 1400317 to 1400847 corresponding to the ECF module operon, FolT1, and FolT2, respectively. Protein sequences used in this study are available through UniProt under the accession codes Q1GBJ0 [https://www.uniprot.org/uniprotkb/Q1GBJ0] for EcfA, Q1GBI9 [https://www.uniprot.org/uniprotkb/Q1GBI9] for EcfA', Q1GBI8 [https://www.uniprot.org/uniprotkb/Q1GBI8] for EcfT, Q1G930 [https://www.uniprot.org/uniprotkb/Q1G930] for FolT1, Q1G292 [https://www.uniprot.org/uniprotkb/Q1G929] for FolT2, and Q1G7W0 [https://www.uniprot.org/uniprotkb/Q1G7W0] for CbrT. For the coarse-grained molecular dynamics simulations, data (final snapshots, cleaned trajectories, starting structure/simulation parameters) are available through the open repository Zenodo [https://doi.org/10.5281/zenodo.8116403]. Source data are provided with this paper.

Research involving human participants, their data, or biological material

Policy information about studies with human participants or human data. See also policy information about sex, gender (identity/presentation), and sexual orientation and race, ethnicity and racism.

Reporting on sex and gender	N/A
Reporting on race, ethnicity, or other socially relevant groupings	N/A
Population characteristics	N/A
Recruitment	N/A
Ethics oversight	N/A

Note that full information on the approval of the study protocol must also be provided in the manuscript.

Field-specific reporting

Please select the one below that is the best fit for your research. If you are not sure, read the appropriate sections before making your selection.

☒ Life sciences ☐ Behavioural & social sciences ☐ Ecological, evolutionary & environmental sciences

For a reference copy of the document with all sections, see nature.com/documents/nr-reporting-summary-flat.pdf

Life sciences study design

All studies must disclose on these points even when the disclosure is negative.

Sample size	No sample size determination was performed. Experiments were performed multiple times with similar results and further inclusion of data did not change the results.
Data exclusions	No data was excluded.
Replication	Experiments were repeated multiple times and the number of replicates and errors are indicated in the manuscript. All attempts at replication were successful.
Randomization	Samples and organisms were not allocated into experimental groups, thus randomization is not relevant for our study. However, single colonies for growth assays were picked randomly.

Blinding	There was no group allocation during data collection and/or analysis making blinding not necessary.

Reporting for specific materials, systems and methods

We require information from authors about some types of materials, experimental systems and methods used in many studies. Here, indicate whether each material, system or method listed is relevant to your study. If you are not sure if a list item applies to your research, read the appropriate section before selecting a response.

Materials & experimental systems

n/a	Involved in the study
☒	☐ Antibodies
☒	☐ Eukaryotic cell lines
☒	☐ Palaeontology and archaeology
☒	☐ Animals and other organisms
☒	☐ Clinical data
☒	☐ Dual use research of concern
☒	☐ Plants

Methods

n/a	Involved in the study
☒	☐ ChIP-seq
☒	☐ Flow cytometry
☒	☐ MRI-based neuroimaging
