# Supplementary material for: Expulsion mechanism of the substrate-translocating subunit in ECF transporters
Source: Nat Commun. 2023 Jul 25;14:4484. doi: 10.1038/s41467-023-40266-1 (PMC10368641; doi:10.1038/s41467-023-40266-1)
Supplement: Supplementary file 2 — Peer Review file [file 41467_2023_40266_MOESM2_ESM.pdf]

nature portfolio

Peer Review File

Expulsion mechanism of the substrate-translocating subunit in ECF transporters

Open Access This file is licensed under a Creative Commons Attribution 4.0 International License, which permits use, sharing, adaptation, distribution and reproduction in any medium or format, as long as you give appropriate credit to the original author(s) and the source, provide a link to the Creative Commons license, and indicate if changes were made. In the cases where the authors are anonymous, such as is the case for the reports of anonymous peer reviewers, author attribution should be to 'Anonymous Referee' followed by a clear attribution to the source work. The images or other third party material in this file are included in the article's Creative Commons license, unless indicated otherwise in a credit line to the material. If material is not included in the article's Creative Commons license and your intended use is not permitted by statutory regulation or exceeds the permitted use, you will need to obtain permission directly from the copyright holder. To view a copy of this license, visit http://creativecommons.org/licenses/by/4.0/.

Reviewers' comments:

Reviewer #1 (Remarks to the Author):

The manuscript led by Thangaratnarajah etal, reported the different conformational structures of the solitary ECF module of ECF transporter complex. Using the whole transporter complex, the authors took numerous efforts in obtaining different conformational structures other that the reported inward-facing conformation, but failed. Then, the authors chose the recombinantly purified solitary ECF moule to continue the try. The major concern is that the EcfT, which is dynamic and couples the EcfA/A' with EcfS, is fully exposed directly to the environmental lipid or artificial detergent or nanodisc in the sample used. Without the presence of EcfS, any changes in the EcfT induced by ATP binding or EcfA/A' closure is a result of its interaction with the binding lipids or detergents, and are most likely artificial. Therefore, the different conformations observed in ATPase-open and close state of solitary ECF module do not necessarily represent the natural feature in ECF transporter complex. The MD analyses may be based on the artificial structure, and the conclusion of the work is largely an over interpretation of the artificial structural data. Several other concerns are listed below:

1. Line 125, The binding of nucleotides to the inward-facing conformation of ABC transports has been reported in a number of structures, i.e., the most recent case ABCG1 (doi.org/10.1016/j.celrep.2022.110298). The fuzzy density for the gama phosphate could be due to the environmental difference, and it should not be listed as supporting evidence for the hypothesis. Here, the Extended Data Fig2a should be cited.

2. Line134, "the Pi-interaction between F13 in EcfA and R174 from FolT12" The density of R174 and F13 should be added. The authors claim that "The direct interaction between S and the nucleotide may protect against the hydrolysis of ATP", and it is an interesting hypothetical point. An ATPase assay needs to be provided to verify the possibility by using mutations. For example, the ATPase activity of protein sample reconstituted in nanodisc and DDM.

3. Line141, As the authors mentioned that ECF-FolT2 complex in DDM rapidly aggregated, does it mean during addition of ATP? While the addition of ATP during purification of ECF complex using detergent buffer had been reported in previous studies of ECF transporters, no such phenomena (aggregation) had been mentioned.

4. Line157, Through recombinant expression and purification, the authors obtained the solitary ECF module complex containing EcfT/A/A', which retain the ATPase activity. Has the authors tested the difference of the ATPase activity between ECF module and the whole transporter? As it has been reported in the literature (doi:10.1038/cr.2017.38) that the group-I ECF module CbiQO also showed a basal ATPase activity comparing to the CbiMNQO, though the Kcat was more than ten-times lower ($2.5 min^{-1}$ vs $29.7 min^{-1}$). It seems that group-I and II ECF transporters share similar character.

5. Typo in Extended Figure-1b/3b/6b/8b, FSC=0.143

6. The authors mentioned that there was an ordered density in the space between the ATPase dimer, and they interpreted it as MSP2N2. Seeing from Extended Figure-7a, the density can also be interpreted as lipids or detergents. Since the protein sample in nanodisc was prepared using DDM purified protein. If so, the conformation of the solitary ECF module could be ruined by the lipid or detergent, in the absence of FolT.

7. Page 8-9, the structural changes here could be artificial, since the EcfT in the solitary ECF module sample is fully exposed to the lipid or detergent. Without the presence of EcfS, any changes in the EcfT induced by ATP binding or EcfA/A' closure is a result of its interaction with the binding lipids or detergent, and are most likely artificial.

8. No representative densities of structural elements were provided.

9. The MD analyses are based on the artificial structure, and the model is highly hypothetical.

Reviewer #2 (Remarks to the Author):

A difficulty in approaching a review of this report is that it is underpinned by an interpretation of a significant body of previous work that, notwithstanding its widespread acceptance, skirts or ignores significant issues in ABC ECF research, in favour of a narrative that defers, without sufficient justification, to the structural data.

In preface, these issues are outlined below.

1. Stoichiometry.

The structures unambiguously require a 1:1:1:1 (ABC:ABC:T:S) subunit stoichiometry for the ECF transport complex. Supporting this, light-scattering experiments with purified group II ECF transporters in detergent solution found that the S, A1, A2 and T subunits mainly exist in a 1:1:1:1 stoichiometry (ter Beek JBC 2011; Karpowich Nat Struct Mol Biol 2015). However, in contradiction, Karpowich and Da-Neng Wang (PNAS 2013) found for the T. maritima ECF Riboflavin Transporter expressed in E. coli, a subunit stoichiometry of 2S:2T:1A:1A' and that different S subunits could bind simultaneously to a single transport complex. Moreover, in vivo fluorescence analyses of the subgroup I biotin transporter (BioMNY) of R. capsulatus found that the S unit BioY oligomerizes in the living cell (Finkenwirth Biochem. J. 2010). This finding was further supported by additional analyses in whole membranes and in vivo

(Kirsch J Bact 2012). A two T model is also supported by cross-linking experiments using whole membranes containing the biotin ECF transporter expressed in E. coli, which suggested a dimeric T subunit.

Indeed, such is the uncertainty around this issue, the Uniprot entries for ABC ECF subunits state a subunit stoichiometry of 1:1:2:2 (ABC:ABC:T:S) for the complete transport complex.

Further to this question, Setyawati et al (eLife 2020) co-reconstituted ECF transporters for folate (ECF-FolT2) and pantothenate (ECF-PanT) into proteoliposomes, and assayed kinetics of active transport. Remarkably, the rate of folate transport was 5 fold higher in liposomes containing the ECF-PanT transporter together with free FolT2 than for those containing ECF-FolT2 alone. In addition, pantothenate transport was 2 fold higher for ECF-PanT plus FolT2 than for ECF-PanT alone. Moreover, the rate of pantothenate transport by ECF-PanT was higher in the presence of a higher ECF-PanT: FolT2 ratio of 1:26 than at 1:7., at an ECF-PanT: FolT2 ratio of 1:26. Furthermore, not only were both folate and pantothenate transport rates significantly higher than for transport complexes containing their respective S subunits alone, but the presence of one substrate at levels 50 fold greater than its transport Km still allowed transport of the other at rates elevated relative to those observed for their respective substrate transport complex alone.

All of this is clearly consistent with the findings of Karpowich and Da-Neng Wang (PNAS 2013) that different S subunits can bind simultaneously to a single transport complex, and with the notion that such a heterogeneous complex functions significantly more efficiently than the group II ECF with a single type of S subunit. Indeed, this explanation can provide a simple rationale for the existence of, and salient difference between, groups I and II ECFs, whereas the 1:1:1:1 stoichiometry suggested by the structures cannot. Yet, despite the obvious and direct nature of such inferences, they were ignored in favour of abstruse and unsubstantiated conjectures required by the structures (Setyawati 2020).

In summary while in vitro analyses conducted with purified complexes reconstituted in detergent solution or structural studies in lipid nanobodies support a 1:1:1:1 stoichiometry, those conducted in whole cells, natural membranes and proteoliposomes do not.

2. Dynamic exchange transport mechanism.

While the exchange of different S subunits with the ECF module has been demonstrated, that dissociation of the S subunit from the ECF module is part of each transport cycle, as required by the structures, has not. Rather, evidence supporting exchange of the S subunit with group II ECFs is generally accepted as a proxy for evidence of a dynamic exchange transport mechanism. Against this, Finkenwirth et al. (Biochemistry 2017) found in detergent solution “purified BioM2NY complexes (group I) were stable during all steps, ATP binding was a prerequisite for biotin capture and ATP hydrolysis for subsequent biotin release.” And that “S units of subclass I ECF transporters do not have to dissociate from holotransporter complexes for high-affinity substrate binding.” In addition, Finkenwirth et al. (JBC 2015) found through cross-linking studies of BioM2NY in lipid nanodisks “a reorientation rather than a disruption of the BioY/BioN interface during catalysis.” This study also found that ATP binding stimulated substrate capture by the transport complex, and subsequent ATP hydrolysis led to substrate release. Thus, since there is little basis for expecting markedly different mechanisms between groups I and II

ECFs, particularly on the basis of structural analyses, these Finkenwirth et al. data are in complete disagreement with the dynamic capture and release of the S subunit as being integral to the transport mechanism, and thereby question the veracity of the structures.

3. Toppling mechanism.

The toppling mechanism originated from, and is required by, the structures. Whilst it is generally accepted that the toppled conformation presents the substrate to the cytosol, in fact the substrate binding site would be as close to the centre of the membrane as to the cytosol, toward which there exists no exclusive protein channel to facilitate its exit; the substrate could just as easily diffuse into the lipid bilayer, and indeed its difficult to see how its passage would not be impeded by lipids (see Fig. 1a lower panel in this manuscript. The folate binding site in the S subunit (yellow) extends approximately from the bilayer centre to the start of the grey arrow marked "Exit pathway", no protein blocks the view along the plane of the membrane into the binding site. See also Fig 5 State 1). Moreover, while the deployment of loops that occlude the substrate in the isolated holo-S subunit is inferred to be crucial to the energetics mediating the toppling mechanism, some S subunits do not have equivalent loops.

While Karpowich et al. (Nat Struct Mol Biol 2015) found a pattern of accessibility of loops in the RibU S subunit consistent with the toppled conformation, nevertheless, as the authors state "... these data do not define the relative orientation of RibU in the transporter complex." Moreover, in the detergent preparation used in this study, the ATPase activity showed a Hill coefficient of 1 whereas in the study of Setyawati et al (eLife 2020) discussed above, the ECF in proteoliposomes displayed an ATPase activity with a Hill coefficient of 2, indicating cooperativity between the two ATPase subunits in hydrolysing ATP. Thus, how closely the conformation of the transport complex in the Karpowichet al. (Nat Struct Mol Biol 2015) study resembled that of the natural state is open to question.

Using the structures, coarse grain MD simulations of the holo-S subunit in a lipid bilayer did not find spontaneous toppling events or their precursors on the microscond timescale (Faustino Nat Comm 2020). It is also noteworthy that while in this study the authors found the non-native lipid POPC tended to destabilise the toppled holo-S subunit relative to simulations using a bacterial membrane model, in their in vitro investigation into the exchange of the S subunits discussed above (Setyawati eLife 2020), which is cited as supporting the dynamic exchange transport mechanism, they included POPC in the proteoliposomes, which, according to the authors' own theoretical analysis thus, could have affected the rate of S subunit exchange.

Extensive mutational analysis in vivo of the structurally predicted interface between the S and T subunits in the L. brevis group II pantothenate ECF transporter (Zhang PNAS 2014) found that for 19 distinct mutants, none displayed loss of the S subunit alone from the ECF complex, while 5, inexplicably, displayed significant dissociation between the T subunit and the ATPase dimer. If this interface had been predicted by bioinformatic analysis, for example, such mutational data would be considered inconclusive, at the very best. Moreover, Neubauer et al. J Bacteriol. 2009 found in vivo, that mutations in the T subunit of a Type II ECF resulted in the S subunit copurifying with the ATPase subunits, in the absence of the T subunit. These data are completely unintelligible from the perspective of the structures, in which the S subunit interacts almost exclusively with the T subunit.

Although this is necessarily not an exhaustive and complete appraisal of the data, we believe it nonetheless reasonable to say that if the data were weighted according to how closely the experimental

conditions approached the natural state, and no greater weight were given to structural data than to any other, the structures and their implicit mechanism would be regarded as far from being confirmed.

p 3-4 Introduction. As discussed above, the background presented in the Introduction should be moderated to reflect the totality of the data rather than giving unjustified weight to the structural data.

p 5 "Nucleotide binding fails to induce conformational changes in nanodiscs" The authors attempt to explain the structural data presented here from the perspective that they necessarily represent a physiological state of the transport complex. They therefore assume as correct that which they are trying to show. Where the data deviate from expectations based on this assumption, they resort to generally unsubstantiated conjectures.

Accordingly, since previous work requires a mechanism whereby ATP hydrolysis results in release of the S subunit, the failure of ATP to effect this change is conjectured to be due to restrictions imposed by the the nanodisc assembly. Thus "... the lipid nanodisc assembly may obstruct conformational rearrangements required to expel the S-component as two opposing forces might lead to conformational strain."

Since the transport complex has proceeded to the closed ADP/ATP state, presumably with the convex conformation of the CHs, why could it not remain there awaiting full S subunit release?

Surely, if this were a situation of an equilibrium between two opposing forces we could expect to see at least some alteration in the S/T interface?

The authors presume the open conformation of the ATPase dimer is a stage of the transport cycle, and that an asymmetric ADP/ATP state is "unexpected", when there is ample evidence supporting an alternating asymmetric mechanism for the ABC ATPase dimer, whereby both composite active sites are never simultaneously separated (J&G 2013).

How does hydrolysis of the first ATP cooperatively facilitate hydrolysis of the second ATP (Setyawati 2020) when the ADP/ATP structure does not differ from the structure in the absence of nucleotides or from the double AMP-PNP bound state?

The insertion of the carboxy-terminal end of the S-component into the open composite ATPase active site is presumed to have a physiological significance: however, doesn't the observation that the nanodisc belt protein was positioned in a similar way suggest it may be a non-specific interaction? In the absence of direct evidence that dissociation of the S subunit triggers ATP hydrolysis, what is to distinguish this feature from a meaningless artefact? Moreover, given that this interpretation requires that this feature is a crucial and conserved part of the mechanism for all ECFs, do the sequences of the C-terminal tails of S subunits reflect an interaction with an invariant moiety such as adenine, and the conserved nature of the ABC composite NBS generally? We might even expect a conserved motif at this point?

p 6-7 "In vitro complex assembly and structure of the solitary ECF module"

"Next, we shifted our attention to the solitary ECF module of ECF-FolT2 to avoid hampered S-component expulsion in the nanodisc environment." The presumption here of the veracity an earlier unsubstantiated conjecture reads more like an exercise in subliminal persuasion than scientific discourse

at the highest level: this graphically illustrates the corruptive nature of the underlying conviction that the structures cannot be wrong.

p 7 “Structure of the solitary ECF module in the ATP-bound closed state.”

Here, we have the extraordinary and unprecedented inference, required by the structures, that the coupling helices, which have analogues in all ABCs, are “thrust” from the membrane interface into the lipid environment by ~9 Å. This in turn presumes that the structure of the TMD is correct and thereby the bilayer bounds can be accurately estimated. Importantly, the energetics and physical plausibility of such a scenario are completely unclear.

Since no other data supporting the ECF conformation observed in this analysis are presented, it should also be assessed from a perspective whereby it may represent a non-physiological, perhaps distorted and/or partially denatured form of the enzyme.

Notably, thus, the structure is of a double mutant that has been found in numerous structural studies of closely related ATPases to produce a symmetrical closed NBD dimer. However, there is ample evidence for these and other ABCs that this symmetrical state is not physiological, it is an artefact of the mutations.

Indeed, the ADP/ATP structure observed here suggests the NBD dimer closes and ATP is hydrolysed in one NBS, while the other is blocked by the S subunit tail: this requires an asymmetric conformation of the NBDs. Therefore, the symmetrical ATP/ATP bound NBD sandwich dimer observed here for the 2EQ mutant cannot also be part of the transport mechanism: either only one, or neither, of the two states reported here is a physiological state.

In addition, doubt should be raised that despite the significant alteration in the conformation of the coupling helices, so called because they are expected to mediate coupling between the TMDs and the NBD in all ABCs, the structures do not indicate that this change thereby stimulates or allows ATP hydrolysis in response to the release of the S subunit.

p 9 “Conformation-specific membrane perturbations”

In this section the authors attempt to explain unprecedented and unexpected alterations to the membrane suggested by their cryo-EM structures. Presupposing the structures are correct, they then incorporate these projected differences into the enzyme mechanism largely through unsubstantiated conjecture.

Clearly, another possibility is that the structures are non-native conformations, and the authors are looking at the consequent artefacts. Thus, there may be no mechanistic significance in lipids being displaced by water away from the highly electrostatically charged intracytoplasmic end of the S subunit in the structures, and the membrane being thereby distorted in MD simulations. The orientation of the S subunit may be an artefact; there is nothing to distinguish these possibilities.

With respect to the MD simulations, it is important to remember that they are subject to the same GIGO rule as all computer analysis: that they maintain and accord with the input structures on the 5µs timescale is not meaningful, or diagnostic as to the physiological veracity of their input structures. This is the more so for coarse grain simulations, where the secondary structure is defined and maintained according to the input structure.

In summary, this section comprises a number of conjectures around the role of membrane deformation in the enzyme mechanism that are unprecedented and only supported by relatively brief simulations that are biased by the starting structures.

4. Comments on the proposed mechanism.

According to the structures, the substrate-bound S subunit binds to the apo ECF module with an open NBD dimer, and substrate is released into the cytosol, whereupon ATP binds, the NBD dimer closes and the S subunit is released, triggering ATP hydrolysis and reset.

However, since the isolated ECF module can hydrolyse ATP in vitro, and can close to the sandwich dimer according to the structures, what is to stop the ECF futilely wasting ATP while it awaits binding of the holo-S? Surely the contradictory finding of Finkenwirth et al. (Biochemistry 2017) that “BioM2NY complexes (group I) were stable during all steps, ATP binding was a prerequisite for biotin capture and ATP hydrolysis for subsequent biotin release.” makes more sense in this regard?

Although the ATP/ATP NBD dimer is proposed to close after substrate release, the structures give no insight into how such signalling occurs, since the isolated ATPase dimer structures differ little from the apo full complex, and the apo full complex differs little from the ADP/ATP-bound full complex.

5. Conclusions.

Here the authors find a new conformation of an ATP-bound ABC ECF. In favour of this conformation being physiological, it provides an arguably plausible, if somewhat crude and indeed dubious mechanism by which ATP hydrolysis could eject the S subunit, as required by previous structures and the toppling mechanism. In view of the many reservations inconsistencies discussed above, and the relatively elf-referential nature of the structural understanding of the enzyme mechanism, however, this is not sufficient to justify publication of this report in Nat Comm.

Indeed it might be said that this report represents a high water mark in the splintering of ABC transporter mechanisms into ever more weird, wonderful and ultimately improbable scenarios, dictated by the structural data. Notablty, all the while the soluble ATPase domains remain resolutely constant, not reflecting in anything even approaching proportion the putative structural variations observed in the TMDs, for which there remains no rationale, not even of the most conjectural kind: on this the field remains utterly mute.

Reviewer #3 (Remarks to the Author):

In the manuscript "Expulsion mechanism of the substrate-translocating subunit in ECF tr,ansporters" Thangaratnarajah et al. utilized cryo-EM and single particle analysis to investigate multiple conformations of an ECF transporter. Their data and interpretation suggest a possible mechanism of this membrane protein.

The study is very well planned and executed, the data compelling, and this work's overall quality is also very good. In principle I have only minor comments that the authors will surely be able to address:

Most of the structures were obtained in lipid nanodisc environment -which is totally fine. However, along the manuscript the authors raise several times major concerns about artefacts that were introduced by this environment (See lines 140, 123-124, 120). This is very concerning to read and only resoved in line 185 where a comparison between DDM and ND is presented. I highly recommend to sacrifcie this supsense for the sake of clarity and tone down the statements about the artifically induced conformations.

It seems that Extended Extendend Fig2 is swapped - please correct or else correct all references in the text.

In line 116 the authors refer to (Extended Data I Fig. 1b) which I think is incorrect – instead they wanted to reference Extended Da. Fig. 2b ?

In line 128 and 129 they reference Extended Data Fig 1a – which would be a micrograph ?

Lines 133 and 134... maybe at additional points as well...

In line, 144 the authors state: "Unexpectedly, the protein complex rapidly aggregated, preventing structure determination. Nonetheless, this observation indicates that conformational changes take place under turnover conditions in detergent solution. The aggregation possibly occurred due to the expulsion of the S-component and the associated fission of the micelle leading to the exposure of hydrophobic surfaces to the aqueous environment."

While this could be an explanation for their observation this argument is only valid if it was the addition of ATP Mg2+, etc that caused the proteins to aggregate - no other potentially unkown effect. This can be quickly checked in negative stain with a before and after image of the same protein sample in detergent.

The authors discuss that the nanodisc environment hampers the repulsion of the S-component from the complex. I think this could be a valid explanation but I wonder why they then stick to this particular environment and do not choose another one for this experiment and thus prove this hypothesis.

It would be helpful to label the S-component as such in Figure 1.

Also, it would help to introduce FolT2 as S-component earlier in the text (currently it is in line 154).

Reviewers' comments:

Reviewer #1 (Remarks to the Author):

The manuscript led by Thangaratnarajah etal, reported the different conformational structures of the solitary ECF module of ECF transporter complex. Using the whole transporter complex, the authors took numerous efforts in obtaining different conformational structures other that the reported inward-facing conformation, but failed. Then, the authors chose the recombinantly purified solitary ECF moule to continue the try. The major concern is that the EcfT, which is dynamic and couples the EcfA/A' with EcfS, is fully exposed directly to the environmental lipid or artificial detergent or nanodisc in the sample used. Without the presence of EcfS, any changes in the EcfT induced by ATP binding or EcfA/A' closure is a result of its interaction with the binding lipids or detergents, and are most likely artificial. Therefore, the different conformations observed in ATPase-open and close state of solitary ECF module do not necessarily represent the natural feature in ECF transporter complex. The MD analyses may be based on the artificial structure, and the conclusion of the work is largely an over interpretation of the artificial structural data.

Response:

*We thank the reviewer for taking the time to review our manuscript and providing us with feedback, but we strongly disagree with the notion that "*without the presence of EcfS, any changes in the EcfT induced by ATP binding or EcfA/A' closure is a result of its interaction with the binding lipids or detergents, and are most likely artificial*."*

Both in vivo and in vitro studies have shown that multiple S-component dynamically interact with the same ECF module in type II ECF transporters (1–5)*, and consequently, a solitary ECF module must -at least transiently- exist in the lipid bilayer exposing the docking site for the S-component to the lipid bilayer (this is mentioned in the Introduction, page 3, lines 65-67 and now also in the Results, page 6, lines 175-177). It is therefore not an artificial condition for a solitary ECF module to reside in the lipid bilayer.*

While the structural basis of the docking of different S-components on a shared ECF module (4) is highlighted by the numerous structures of ECF transporter complexes from Lactobacillus brevis [ECF-FolT (6), ECF-PanT (7) and ECF-HmpT (8)] and Lactobacillus delbrueckii [ECF-FolT2 (9), ECF-CbrT (10) and ECF-PanT (2)], the release of an S-component from the ECF module and the (transient) exposure of the docking site for the S-component to the lipid bilayer are structurally and mechanistically poorly understood. Therefore, the study of a solitary ECF module in the lipid bilayer is of high relevance.

We show in our manuscript that a solitary ECF module in the absence of a co-expressed S-component can be purified as a stable complex, which has been also reported for the Type I cobalt ECF transporter (11) and the Type II riboflavin ECF transporter (12). We also showed that the purified solitary ECF module retained ATPase activity and more importantly the capacity to interact and drive substrate transport when co-reconstituted into liposomes with a separately purified S-component (Figure 2b). Therefore, the purified solitary module is not trapped in an inactive state and is a functionally relevant complex.

The structure of the solitary ECF module in the nucleotide-free state (Figure 1b) reveals that the structural integrity in the absence of an S-component is maintained. Here, the solitary ECF module still adopts the same conformation as the ECF module in the ECF transporter complexes from the same species, namely ECF-FolT2 (Figure 1a), ECF-PanT, and ECF-CbrT (rmsd is between 1.6-1.8 Å). It also reveals that the docking site for the S-component is intact and not altered as a consequence of the purification of the solitary ECF module in the absence of an S-component, again indicating structural integrity of the module.

The docking site for the S-component in EcfT is hydrophobic and non-polar in nature (Extended Data Figure 10f and g), and therefore compatible to interact with the surrounding environment i.e. lipids, lipid nanodisc or detergent micelle in the absence of a bound S-component. The exposure of binding/interaction sites to the surrounding lipid or detergent environment has been observed also in other membrane protein structures. For example, the ER membrane protein complex (EMC) is responsible for inserting transmembrane helices into the lipid bilayer. Cryo-EM structures of human and yeast EMC complexes reveal a hydrophobic vestibule with an intramembrane opening, which is exposed

to the surrounding environment i.e. lipid nanodiscs or detergent micelle and serves as a pathway for transmembrane helix insertion (13–16).

Apart from the relative orientation of the S-component with respect to the lipid bilayer plane, large structural rearrangements of the transmembrane helices of S-components are not expected. The structures of the substrate-bound and substrate-free S-components only differ in the conformation of the extracellular gating loops while the rest of the S-component remains largely static (7, 9). This suggests that conformational changes are expected in the ECF module that drive the reorientation and expulsion of the S-component, consistent with our observations.

We therefore conclude that we have structurally captured the solitary ECF module in a lipid nanodisc environment in a physiologically and functionally relevant state.

Several other concerns are listed below:

1. Line 125, The binding of nucleotides to the inward-facing conformation of ABC transports has been reported in a number of structures, i.e., the most recent case ABCG1 (doi.org/10.1016/j.celrep.2022.110298). The fuzzy density for the gama phosphate could be due to the environmental difference, and it should not be listed as supporting evidence for the hypothesis. Here, the Extended Data Fig2a should be cited.

Response:

We have now corrected this and changed it from Extended Data Fig. 1a to Extended Data Fig. 3a. We also thank the reviewer for pointing out the cryo-EM structure of ABCG1 (17), as an example for an nucleotide-bound ABC transporter in the inward-facing conformation. We would like to point out that a mutant variant of ABCG1 (the Walker B mutant E242Q) was used for the single particle cryo-EM analysis, which is expected to bind ATP but is either severely impaired in hydrolysing ATP, or is completely inactive. Moreover, it is not stated in the 'Materials and Methods' section of the article at what temperature the sample was incubated with 10 mM Mg-ATP for 15 min prior to sample vitrification, which would affect the hydrolysis rate of ATP. In our case, we used the 'wild-type' folate-specific ECF transporter complex (ECF-FolT2), which is fully competent to hydrolyse ATP as previously shown (18), and incubated our sample with 5 mM Mg-ATP at 37°C for 10 min prior to sample vitrification. The asymmetric occupancy of ATP in the nucleotide-binding site of EcfA and ADP in the nucleotide-binding site of EcfA' in the structure of ECF-FolT2$_{ATP}$ was indeed unexpected, an observation made also in other ABC transporters e.g. TmrAB (19). We have now clarified this by extending following sentence in our manuscript (underlined):

Page 5, line 128: "The nucleotide occupancy in the nucleotide-binding sites of ECF-FolT2ATP is asymmetric with ATP and ADP bound to the EcfA and EcfA' subunits, respectively (Extended Data Fig. 3a), <u>which has been also observed in other ABC transporters[21]</u>."

Moreover, we are also confident that ATP is bound in the nucleotide-binding site of EcfA because the equivalent structure with the non-hydrolysable ATP analogue AMP-PNP (ECF-FolT2$_{AMP\text{-}PNP}$) shows a similar density in the nucleotide-binding site of EcfA (Extended Data Fig. 3b).

2. Line134, "the Pi-interaction between F13 in EcfA and R174 from FolT12" The density of R174 and F13 should be added. The authors claim that "The direct interaction between S and the nucleotide may protect against the hydrolysis of ATP", and it is an interesting hypothetical point. An ATPase assay needs to be provided to verify the possibility by using mutations. For example, the ATPase activity of protein sample reconstituted in nanodisc and DDM.

Response:

We have now included the density for F13$_{EcfA}$ and R174$_{FolT2}$ in Extended Data Fig. 3a and also 3b. We agree with the reviewer that our interpretation of the observed interaction is hypothetical, and agree that future experiments are needed to test the hypothesis further, but we also find it beyond the scope of our manuscript. We did not aim to solve the structure of the full complex in the open conformation with nucleotides bound, we rather aimed for previously unseen conformations which could explain the mechanism of S-component expulsion. The complex just happened to remain in the open state. We now emphasize in the text that the interpretation on the consequence of the interaction is speculative, as follows:

In the results section:

Page 6, line 158: "<u>We hypothesise that the</u> direct interaction between the S-component and the nucleotide could affect the hydrolysis of ATP."

and in the discussion:

Page 13, line 442: "This observation <u>may</u> hint at the requirement of S-component expulsion associated with ATP hydrolysis at the second site."

3. Line141, As the authors mentioned that ECF-FolT2 complex in DDM rapidly aggregated, does it mean during addition of ATP? While the addition of ATP during purification of ECF complex using detergent buffer had been reported in previous studies of ECF transporters, no such phenomena (aggregation) had been mentioned.

Response:

The ECF-FolT2 complex in DDM micelles visibly aggregated (turbid solution) upon incubation with Mg-ATP prior to sample vitrification (but not without addition of Mg-ATP). A similar behaviour was also observed with Mg-AMP-PNP. Indeed, the purification of the ECF transporter complex in the presence of Mg-ATP has been reported previously (1). In that case, no visible aggregation of the protein sample has been mentioned. Possibly, the lack of visible aggregation was due to the low protein concentration used in those experiments, while we used high protein concentrations (> 6 mg/ml) when Mg-ATP or Mg-AMP-PNP was added only at the final step. As high protein concentrations were required for adequate particle distribution for single particle analysis of the ECF transporter complex in DDM micelles, we could not use much lower protein concentrations for structure determination. We have now made it clear in the text as follows:

Page 6, line 165: "However, the protein complex rapidly aggregated <u>prior to sample vitrification</u>, preventing structure determination. "

We also note that the ECF transporter that Karpowich et al. (1) reported on is not the same as the one we have studied. It is well known that protein homologues may behave differently from each in biochemical procedures.

4. Line157, Through recombinant expression and purification, the authors obtained the solitary ECF module complex containing EcfT/A/A', which retain the ATPase activity. Has the authors tested the difference of the ATPase activity between ECF module and the whole transporter? As it has been reported in the literature (doi:10.1038/cr.2017.38) that the group-I ECF module CbiQO also showed a basal ATPase activity comparing to the CbiMNQO, though the Kcat was more than ten-times lower (2.5min-1 vs 29.7min-1). It seems that group-I and II ECF transporters share similar character.

Response:

We have now tested the ATPase activity of the ECF module (data from 2 biological repeats) in complex with an S-component (ECF-FolT2) using the same assay as we used for the ECF module alone (see figure below).

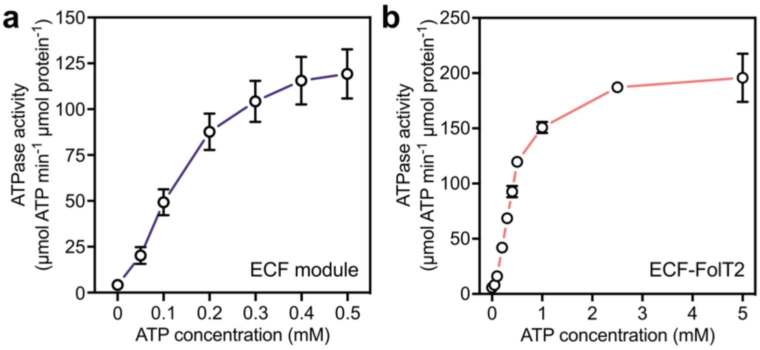

The presence of the S-component reduces the Km value (ECF module - 136.2 µM vs ECF-FolT2 – 489.4 µM). This is possibly caused by unequal alteration of the two ATP hydrolysis sites when the S-component is bound, maybe through direct interaction with one of the ATP molecules. Of course, this is a descriptive rather than full mechanistic interpretation, but the differences in Km values are certainly not at odds with the structures. The Vmax values only differ ~1.5 fold (ECF module - 132.6 µmol min^{-1} $µmol^{-1}$ vs ECF-FolT2 – 212.3 µmol min^{-1} $µmol^{-1}$). Given how difficult it is to accurately determine protein concentrations (including the differences in reconstitution efficiency in nanodiscs, with the amount of empty nanodiscs in the two samples likely varying), we believe that we should not over-interpret such differences.

We are aware that the differences in ATPase activity in a similar experiment using the Type I cobalt ECF transporter are much bigger i.e. over 10-fold (11). Notably, this is a completely different protein complex as the ECF module contains two identical ATPases, while in our protein complex, the ECF module contains two unidentical ATPases EcfA and EcfA', which share around 34 % sequence identity. We believe that it is not a good idea to use CbiMNQO biochemical data to interpret our structures.

5. Typo in Extended Figure-1b/3b/6b/8b, FSC=0.143.

Response:

The typo has been corrected in the above-mentioned figures.

6. The authors mentioned that there was an ordered density in the space between the ATPase dimer, and they interpreted it as MSP2N2. Seeing from Extended Figure-7a, the density can also be interpreted as lipids or detergents. Since the protein sample in nanodisc was prepared using DDM purified protein. If so, the conformation of the solitary ECF module could be ruined by the lipid or detergent, in the absence of FolT.

Response:

The density in the space between the ATPase dimer can be fitted with an alpha helix that is around 30 residues long (see figure below). We therefore attributed this density to the MSP2N2 belt protein. Nonetheless, we cannot categorially identify species giving the density, and it is likely that the hydrophobic surface can also interact with lipids, as it should in a bilayer environment. All of this is consistent with the natural function of the protein, as discussed above.

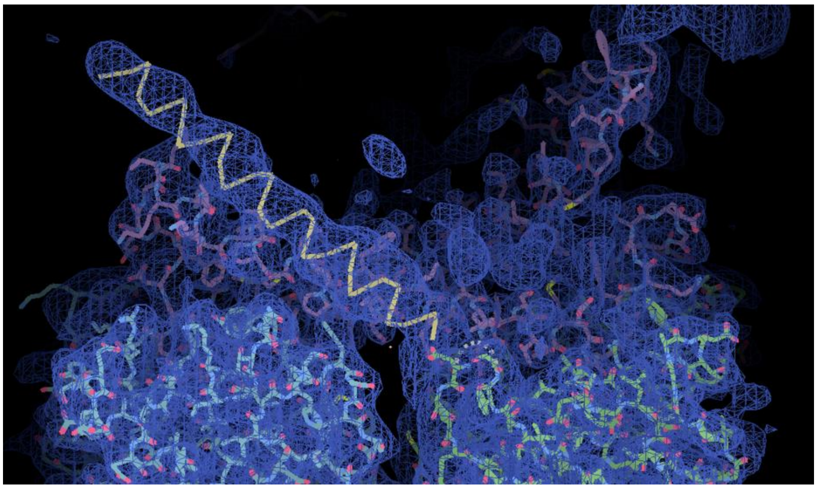

7. Page 8-9, the structural changes here could be artificial, since the EcfT in the solitary ECF module sample is fully exposed to the lipid or detergent. Without the presence of EcfS, any changes in the EcfT induced by ATP binding or EcfA/A' closure is a result of its interaction with the binding lipids or detergent, and are most likely artificial.

Response:

We have addressed this comment extensively above: The docking site for the S-component in EcfT is hydrophobic and non-polar in nature (Extended Data Figure 10f and g), and therefore compatible to interact with the surrounding environment i.e. lipids, lipid nanodisc or detergent micelle in the absence of a bound S-component. This is in fact a natural (non-artificial) function and (at least transient) state of the protein.

8. No representative densities of structural elements were provided.

Response:

We have now included density maps of selected regions for each structure (see Extended Data Figure 2, 5, 9, and 14) The density maps are coloured according to the local resolution estimation highlighting the differences in resolution. The numbering in the main text has been amended accordingly.

9. The MD analyses are based on the artificial structure, and the model is highly hypothetical.

Response:

We have elaborated above that the structure of the solitary ECF module is a physiologically and functionally relevant state.

Reviewer #2 (Remarks to the Author):

A difficulty in approaching a review of this report is that it is underpinned by an interpretation of a significant body of previous work that, notwithstanding its widespread acceptance, skirts or ignores significant issues in ABC ECF research, in favour of a narrative that defers, without sufficient justification, to the structural data.

We find it very difficult to address this reviewer's comments. The reviewer appears to profoundly dislike the entire field of structural biology, and appears to project his/her aversion on our manuscript. In general, we would say that biochemists and structural biologists alike are very much aware of the fact that they are studying biological macromolecules outside a living cell, which -by definition- is an artificial environment. Therefore, we and others take great care in scrutinizing data (from whichever source) to provide a hypothesis for a mechanistic model. This is also the reason why we had incorporated in vivo studies in our manuscript (Extended Data Figure 12). It is usually not possible to incorporate all available data in one single consistent model, sometimes because data quality is occasionally problematic, sometimes because the data come from different model systems, each with their own peculiarities. We find some of the comments below pretty outrageous, non-scientific, destructive and unprofessional, as we will try to explain.

In preface, these issues are outlined below.

1. Stoichiometry.

The structures unambiguously require a 1:1:1:1 (ABC:ABC:T:S) subunit stoichiometry for the ECF transport complex. Supporting this, light-scattering experiments with purified group II ECF transporters in detergent solution found that the S, A1, A2 and T subunits mainly exist in a 1:1:1:1 stoichiometry (ter Beek JBC 2011; Karpowich Nat Struct Mol Biol 2015). However, in contradiction, Karpowich and Da-Neng Wang (PNAS 2013) found for the T. maritima ECF Riboflavin Transporter expressed in E. coli, a subunit stoichiometry of 2S:2T:1A:1A′ and that different S subunits could bind simultaneously to a single transport complex. Moreover, in vivo fluorescence analyses of the subgroup I biotin transporter (BioMNY) of R. capsulatus found that the S unit BioY oligomerizes in the living cell (Finkenwirth Biochem. J. 2010). This finding was further supported by additional analyses in whole membranes and in vivo (Kirsch J Bact 2012). A two T model is also supported by cross-linking experiments using whole membranes containing the biotin ECF transporter expressed in E. coli, which suggested a dimeric T subunit.
Indeed, such is the uncertainty around this issue, the Uniprot entries for ABC ECF subunits state a subunit stoichiometry of 1:1:2:2 (ABC:ABC:T:S) for the complete transport complex

Response:

Numerous crystal (2, 6–11) and cryo-EM structures (18) of ECF transporters complexes from 3 different organisms, with different substrate specificities, and from different laboratories over the last 10 years unequivocally demonstrate that the stable core of ECF transporter complexes exist in a 1:1:1:1 (A:A:T:S) subunit stoichiometry. Notably, all studies contradicting the 1:1:1:1: subunit stoichiometry were published before April 2013, when the first crystal structures of ECF transporter complexes became available (6, 8). It is well known that crosslinking and in vivo fluorescence methods must be interpreted with care, and can give rise to artefacts, just like any other method. While our data does not categorically exclude the existence of higher order super-complexes in vivo, our data as well as the majority of the existing data on ECF transporters (in vitro and in vivo) show that a 1:1:1:1 (A:A:T:S) subunit stoichiometry provides a stable and functional core complex. As such a mechanistic model for such a complex is legit and required.

As for the Uniprot entries, this reviewer comment is extremely problematic, because the reviewer chooses to ignore the well-known limitations of Uniprot annotations. Uniprot data on the subunit stoichiometry are based on an automatic annotation rule (UniRule HAMAP-Rule: MF_01710) or by similarity. There are also entries stating both subunit stoichiometries.

For example - Uniprot entry A2RI02:

Interaction

Subunit

Forms a stable energy-coupling factor (ECF) transporter complex composed of 2 membrane-embedded substrate-binding proteins (S component), 2 ATP-binding proteins (A component) and 2 transmembrane proteins (T component). In L.lactis forms a stable complex with EcfA' and EcfT and substrate-binding components. In E.coli forms a stable complex with EcfA, EcfT and individually with 3 tested substrate-binding components (BioY, NiaX and ThiT) with a stoichiometry of 1.1:1:1. The core ECF complex interacts with a number of substrate-specific binding components, including BioY, BioY2, HmpT, NiaX, PanT, QueT, RibU and ThiT. UniRule Annotation 1 Publication

Protein-protein interaction databases

STRING | 416870.llmg_0288

(https://www.uniprot.org/uniprotkb/A2RI02/entry#interaction – accessed 26.02.2023 at 11:15)

The reviewer seems to be cherry picking Uniprot entries, which by themselves already cannot be taken at face value.

Further to this question, Setyawati et al (eLife 2020) co-reconstituted ECF transporters for folate (ECF-FolT2) and pantothenate (ECF-PanT) into proteoliposomes, and assayed kinetics of active transport. Remarkably, the rate of folate transport was 5 fold higher in liposomes containing the ECF-PanT transporter together with free FolT2 than for those containing ECF-FolT2 alone. In addition, pantothenate transport was 2 fold higher for ECF-PanT plus FolT2 than for ECF-PanT alone. Moreover, the rate of pantothenate transport by ECF-PanT was higher in the presence of a higher ECF-PanT: FolT2 ratio of 1:26 than at 1:7., at an ECF-PanT: FolT2 ratio of 1:26. Furthermore, not only were both folate and pantothenate transport rates significantly higher than for transport complexes containing their respective S subunits alone, but the presence of one substrate at levels 50 fold greater than its transport Km still allowed transport of the other at rates elevated relative to those observed for their respective substrate transport complex alone.

All of this is clearly consistent with the findings of Karpowich and Da-Neng Wang (PNAS 2013) that different S subunits can bind simultaneously to a single transport complex, and with the notion that such a heterogeneous complex functions significantly more efficiently than the group II ECF with a single type of S subunit. Indeed, this explanation can provide a simple rationale for the existence of, and salient difference between, groups I and II ECFs, whereas the 1:1:1:1 stoichiometry suggested by the structures cannot. Yet, despite the obvious and direct nature of such inferences, they were ignored in favour of abstruse and unsubstantiated conjectures required by the structures (Setyawati 2020).

In summary while in vitro analyses conducted with purified complexes reconstituted in detergent solution or structural studies in lipid nanobodies support a 1:1:1:1 stoichiometry, those conducted in whole cells, natural membranes and proteoliposomes do not.

Response:

We feel that the reviewer now starts repeating the careful review process of other journals and past studies, and we do not think that this is the right place to do so. Only one remark: co-reconstitution of multiple proteins in a single reconstitution process often gives rise to different protein activities than separate reconstitutions of the individual components. Therefore, great care has to be taken not to overinterpret absolute activity values when comparing reconstitutions that took place in very different conditions.

Also of note: Karpowich and Da-Neng Wang's later paper in 2015 reports on a 1:1:1:1 stoichiometry (1).

2. Dynamic exchange transport mechanism.

While the exchange of different S subunits with the ECF module has been demonstrated, that dissociation of the S subunit from the ECF module is part of each transport cycle, as required by the structures, has not. Rather, evidence supporting exchange of the S subunit with group II ECFs is generally accepted as a proxy for evidence of a dynamic exchange transport mechanism. Against this, Finkenwirth et al. (Biochemistry 2017) found in detergent solution "purified BioM2NY complexes (group I) were stable during all steps, ATP binding was a prerequisite for biotin capture and ATP hydrolysis for subsequent biotin release." And that "S units of subclass I ECF transporters do not have to dissociate from holotransporter complexes for high-affinity substrate binding." In addition, Finkenwirth et al. (JBC 2015) found through cross-linking studies of BioM2NY in lipid nanodisks "a reorientation rather than a disruption of the BioY/BioN interface during catalysis." This study also found that ATP binding stimulated substrate capture by the transport complex, and subsequent ATP hydrolysis led to substrate release. Thus, since there is little basis for expecting markedly different mechanisms between groups I and II ECFs, particularly on the basis of structural analyses, these Finkenwirth et al. data are in complete

disagreement with the dynamic capture and release of the S subunit as being integral to the transport mechanism, and thereby question the veracity of the structures.

Response:

BioM2NY is a group I ECF transporter and a very different system from ECF-FolT2, which is a group II ECF transporter. Our work is on ECF-FolT2, for which a much more complete set of data is available than for BioM2NY.

3. Toppling mechanism.

The toppling mechanism originated from, and is required by, the structures. Whilst it is generally accepted that the toppled conformation presents the substrate to the cytosol, in fact the substrate binding site would be as close to the centre of the membrane as to the cytosol, toward which there exists no exclusive protein channel to facilitate its exit; the substrate could just as easily diffuse into the lipid bilayer, and indeed its difficult to see how its passage would not be impeded by lipids (see Fig. 1a lower panel in this manuscript. The folate binding site in the S subunit (yellow) extends approximately from the bilayer centre to the start of the grey arrow marked “Exit pathway”, no protein blocks the view along the plane of the membrane into the binding site. See also Fig 5 State 1). Moreover, while the deployment of loops that occlude the substrate in the isolated holo-S subunit is inferred to be crucial to the energetics mediating the toppling mechanism, some S subunits do not have equivalent loops.

Response:

Unlike what the reviewer claims, the cytosolic exit path is accessible from the cytosol. The membrane is thinned at that location. Some S-components indeed do not have long loops covering the substrate binding site (yet still short loops!), and those S-components may or may not behave differently from FolT2. We describe data on ECF-FolT2 because this is a system on which a multitude data from completely different types of experiments is available.

While Karpowich et al. (Nat Struct Mol Biol 2015) found a pattern of accessibility of loops in the RibU S subunit consistent with the toppled conformation, nevertheless, as the authors state “... these data do not define the relative orientation of RibU in the transporter complex.” Moreover, in the detergent preparation used in this study, the ATPase activity showed a Hill coefficient of 1 whereas in the study of Setyawati et al (eLife 2020) discussed above, the ECF in proteoliposomes displayed an ATPase activity with a Hill coefficient of 2, indicating cooperativity between the two ATPase subunits in hydrolysing ATP. Thus, how closely the conformation of the transport complex in the Karpowichet al. (Nat Struct Mol Biol 2015) study resembled that of the natural state is open to question.

Response:

We find that this comment does not have anything to do with our manuscript. This is really awkward as this comment relates to the article by Karpowich et al. (1).

Using the structures, coarse grain MD simulations of the holo-S subunit in a lipid bilayer did not find spontaneous toppling events or their precursors on the microscond timescale (Faustino Nat Comm 2020). It is also noteworthy that while in this study the authors found the non-native lipid POPC tended to destabilise the toppled holo-S subunit relative to simulations using a bacterial membrane model, in their in vitro investigation into the exchange of the S subunits discussed above (Setyawati eLife 2020), which is cited as supporting the dynamic exchange transport mechanism, they included POPC in the proteoliposomes, which, according to the authors’ own theoretical analysis thus, could have affected the rate of S subunit exchange.

Response:

Again, we find that this comment does not have anything to do with our manuscript. Even so, the reviewer is mistaken here. We always add a fraction of PC to our reconstitutions, which is completely fine as long as there is a sufficient amount of other lipids present.

Extensive mutational analysis in vivo of the structurally predicted interface between the S and T subunits in the L. brevis group II pantothenate ECF transporter (Zhang PNAS 2014) found that for 19 distinct mutants, none displayed loss of the S subunit alone from the ECF complex, while 5, inexplicably,

displayed significant dissociation between the T subunit and the ATPase dimer. If this interface had been predicted by bioinformatic analysis, for example, such mutational data would be considered inconclusive, at the very best.

Response:

In contrast to what the reviewer suggests, the interface does consist of a conserved motive (AxxxA) in the S-component. EcfT does not have to be conserved between species, as long as it is compatible with S-component interaction within a single species. The fact that mutagenesis studies are considered inconclusive is not uncommon.

Moreover, Neubauer et al. J Bacteriol. 2009 found in vivo, that mutations in the T subunit of a Type II ECF resulted in the S subunit copurifying with the ATPase subunits, in the absence of the T subunit. These data are completely unintelligible from the perspective of the structures, in which the S subunit interacts almost exclusively with the T subunit.

Response:

This result is perhaps indeed difficult to explain at this moment, but should we really make this experiment the basis for rejecting a largely consistent model? We think not. In addition, the experiment described also has its own possible flaws, related to the biochemistry (out of the in vivo situation) of mutated, and possible aggregating complexes.

Although this is necessarily not an exhaustive and complete appraisal of the data, we believe it nonetheless reasonable to say that if the data were weighted according to how closely the experimental conditions approached the natural state, and no greater weight were given to structural data than to any other, the structures and their implicit mechanism would be regarded as far from being confirmed.

Response:

We disagree. Although perhaps not all available data (collected on diverse proteins) can be incorporated in a single model yet, we believe that our interpretation is consistent with most of the data at present. Nonetheless, it may eventually be wrong, which is how the scientific process works. We believe that mechanistic models are an essential part of this scientific discourse.

p 3-4 Introduction. As discussed above, the background presented in the Introduction should be moderated to reflect the totality of the data rather than giving unjustified weight to the structural data.

Response:

Due to the word limit, we scarified the totality of field for introducing the structural data because of the nature of the results presented in our manuscript. A future review may be more appropriate.

p 5 "Nucleotide binding fails to induce conformational changes in nanodiscs" The authors attempt to explain the structural data presented here from the perspective that they necessarily represent a physiological state of the transport complex. They therefore assume as correct that which they are trying to show. Where the data deviate from expectations based on this assumption, they resort to generally unsubstantiated conjectures.
Accordingly, since previous work requires a mechanism whereby ATP hydrolysis results in release of the S subunit, the failure of ATP to effect this change is conjectured to be due to restrictions imposed by the the nanodisc assembly. Thus "... the lipid nanodisc assembly may obstruct conformational rearrangements required to expel the S-component as two opposing forces might lead to conformational strain."
Since the transport complex has proceeded to the closed ADP/ATP state, presumably with the convex conformation of the CHs, why could it not remain there awaiting full S subunit release?
Surely, if this were a situation of an equilibrium between two opposing forces we could expect to see at least some alteration in the S/T interface?

Response:

We state that the full folate-specific ECF transporter complex (ECF-FolT2) did not undergo conformational changes and is identical to the conformation of ECF-FolT2 in the absence of nucleotides

i.e ECF-FolT2 did not proceed to the to a closed conformation in the presence of nucleotides (page 5, lines 102-106). Accordingly, we did not observe any conformational changes at the interface between EcfT and the S-component. We do not understand why "surely" we should expect to see some alteration in the S/T interface. Given the nature of single particle cryo-EM analysis, we do not see why this surely should happen.

The authors presume the open conformation of the ATPase dimer is a stage of the transport cycle, and that an asymmetric ADP/ATP state is "unexpected", when there is ample evidence supporting an alternating asymmetric mechanism for the ABC ATPase dimer, whereby both composite active sites are never simultaneously separated (J&G 2013).

Response:

As stated above, the asymmetric occupancy of ATP in the nucleotide-binding site of EcfA and ADP in the nucleotide-binding site of EcfA' in the structure of ECF-FolT2$_{ATP}$ was indeed unexpected, and there is evidence for this in the literature. We apologise for not citing this work and clarified this by extending following sentence in our manuscript (underlined):

Page 5, line 128: "The nucleotide occupancy in the nucleotide-binding sites of ECF-FolT2ATP is asymmetric with ATP and ADP bound to the EcfA and EcfA' subunits, respectively (Extended Data Fig. 3a), <u>which has been also observed in other ABC transporters[21]</u>."

How does hydrolysis of the first ATP cooperatively facilitate hydrolysis of the second ATP (Setyawati 2020) when the ADP/ATP structure does not differ from the structure in the absence of nucleotides or from the double AMP-PNP bound state?

Response:

We do not understand what the issue is that the reviewer seems to create. The ADP/ATP *and* AMP-PNP/AMP-PNP *bound structures are in the open conformation. To hydrolyse one ATP the protein must have gone through a more closed conformation, at least for one of the ATPase sites. However, this (possibly transient) conformation is not captured it the cryo-EM analysis.*

The insertion of the carboxy-terminal end of the S-component into the open composite ATPase active site is presumed to have a physiological significance: however, doesn't the observation that the nanodisc belt protein was positioned in a similar way suggest it may be a non-specific interaction? In the absence of direct evidence that dissociation of the S subunit triggers ATP hydrolysis, what is to distinguish this feature from a meaningless artefact? Moreover, given that this interpretation requires that this feature is a crucial and conserved part of the mechanism for all ECFs, do the sequences of the C-terminal tails of S subunits reflect an interaction with an invariant moiety such as adenine, and the conserved nature of the ABC composite NBS generally? We might even expect a conserved motif at this point?

Response:

The carboxy-terminal end of various S-components is indeed conserved in form of the presence of positively charged residues as indicated on page 6 lines 157-158 in our manuscript.

p 6-7 "In vitro complex assembly and structure of the solitary ECF module"
"Next, we shifted our attention to the solitary ECF module of ECF-FolT2 to avoid hampered S-component expulsion in the nanodisc environment." The presumption here of the veracity an earlier unsubstantiated conjecture reads more like an exercise in subliminal persuasion than scientific discourse at the highest level: this graphically illustrates the corruptive nature of the underlying conviction that the structures cannot be wrong.

Response:

Well, the language becomes rather unscientific and personal here. We will counter with data: It is well known that nanodiscs can restrict the conformational freedom of embedded membrane proteins. This has been shown for example for the ABC transporter MsbA (20). We ourselves have preliminary experimental data on a different ECF transporter showing that full S-component release is not possible in nanodiscs.

p 7 “Structure of the solitary ECF module in the ATP-bound closed state.”
Here, we have the extraordinary and unprecedented inference, required by the structures, that the coupling helices, which have analogues in all ABCs, are “thrust” from the membrane interface into the lipid environment by ~9 Å. This in turn presumes that the structure of the TMD is correct and thereby the bilayer bounds can be accurately estimated. Importantly, the energetics and physical plausibility of such a scenario are completely unclear.

Response:

Molecular dynamics simulations show the energetic and physical plausibility.

Since no other data supporting the ECF conformation observed in this analysis are presented, it should also be assessed from a perspective whereby it may represent a non-physiological, perhaps distorted and/or partially denatured form of the enzyme.

Response:

The reviewer is correct in his/her statement that the 2EQ mutant variant is a mutant, and that interpretation of a mutant structure should always be done with care. The reviewer is also right that a mutant by definition is non-physiological, because, yes, it is a mutant, but the remark about denaturisation is unfounded, as explained in the response to reviewer 1.

Notably, thus, the structure is of a double mutant that has been found in numerous structural studies of closely related ATPases to produce a symmetrical closed NBD dimer. However, there is ample evidence for these and other ABCs that this symmetrical state is not physiological, it is an artefact of the mutations. Indeed, the ADP/ATP structure observed here suggests the NBD dimer closes and ATP is hydrolysed in one NBS, while the other is blocked by the S subunit tail: this requires an asymmetric conformation of the NBDs. Therefore, the symmetrical ATP/ATP bound NBD sandwich dimer observed here for the 2EQ mutant cannot also be part of the transport mechanism: either only one, or neither, of the two states reported here is a physiological state.

Response:

This particular mutant has been instrumental in the analysis of the ATP binding sites in many ABC transporters and induces a symmetrical conformation because ATP hydrolysis is prevented or slowed down. This exact symmetrical conformation may not occur in the wild-type, but nonetheless it provides extremely helpful insights, just like it has done for a plethora of other ABC transporters (17, 19, 21–30). In all these cases there simply has been no other way to structurally probe the ATP hydrolysis site. Does the reviewer question the validity of all this published work in the ABC transporter field? While we certainly respect other viewpoints and disagreements, which is what scientific discourse is all about, is it not poor reviewing practice to project all of this frustration on a single manuscript? Would it not be more appropriate to voice these concerns in a review for an open scientific discussion, similar to what has been published by Jones and George in FEBS Letters (31). Finally, it should be noted that the mutant variant we used is not completely dead (Extended Data Figure 12). The residual activity is an argument to assume that by and large there is not much artificial about the protein.

In addition, doubt should be raised that despite the significant alteration in the conformation of the coupling helices, so called because they are expected to mediate coupling between the TMDs and the NBD in all ABCs, the structures do not indicate that this change thereby stimulates or allows ATP hydrolysis in response to the release of the S subunit.

Response:

We do not see why this should be a mechanistic problem. Futile ATPase activity (note that is in the order of 2 per second only, and that the expression levels of the ECF module are low in vivo) is found in many systems in vivo. As long as it serves a purpose by providing a selective advantage (for instance acquiring an important micronutrient) there is nothing problematic about futile ATP hydrolysis. It should be noted that there are even more expensive ways of acquiring micronutrient: For instance, humans take up cobalamin by hydrolysing the 400 amino acid protein intrinsic factor. It thus costs about 2000 ATP molecules to import a single cobalamin. Still we have evolved to do so.

p 9 “Conformation-specific membrane perturbations”
In this section the authors attempt to explain unprecedented and unexpected alterations to the membrane suggested by their cryo-EM structures. Presupposing the structures are correct, they then incorporate these projected differences into the enzyme mechanism largely through unsubstantiated conjecture.
Clearly, another possibility is that the structures are non-native conformations, and the authors are looking at the consequent artefacts. Thus, there may be no mechanistic significance in lipids being displaced by water away from the highly electrostatically charged intracytoplasmic end of the S subunit in the structures, and the membrane being thereby distorted in MD simulations. The orientation of the S subunit may be an artefact; there is nothing to distinguish these possibilities.

Response:

If the reviewer is so convinced that high resolution structures must be artefacts, then there is not much point further arguing. We have explained above why we believe that we are not looking at gross artefacts.

With respect to the MD simulations, it is important to remember that they are subject to the same GIGO rule as all computer analysis: that they maintain and accord with the input structures on the 5µs timescale is not meaningful, or diagnostic as to the physiological veracity of their input structures. This is the more so for coarse grain simulations, where the secondary structure is defined and maintained according to the input structure.
In summary, this section comprises a number of conjectures around the role of membrane deformation in the enzyme mechanism that are unprecedented and only supported by relatively brief simulations that are biased by the starting structures.

Response:

In contrast to the reviewer’s statement, for each system we actually performed 5 replicas of 15 µs (so, a total of 75 µs of sampling for each system) and not 5 µs. Considering the speed-up of CG models, this sampling possibly represents in real time 2-4 times this sampling. Combined with the previous work (32), we have (one of) the largest sampling anyone ever performed for ECFs before. For sure we can get more sampling or even run all-atom simulations to refine predictions. However, with this line of arguments of the reviewer, any simulation data ever published in Nature Communication should be discarded.

4. Comments on the proposed mechanism.
According to the structures, the substrate-bound S subunit binds to the apo ECF module with an open NBD dimer, and substrate is released into the cytosol, whereupon ATP binds, the NBD dimer closes and the S subunit is released, triggering ATP hydrolysis and reset.
However, since the isolated ECF module can hydrolyse ATP in vitro, and can close to the sandwich dimer according to the structures, what is to stop the ECF futilely wasting ATP while it awaits binding of the holo-S? Surely the contradictory finding of Finkenwirth et al. (Biochemistry 2017) that “BioM2NY complexes (group I) were stable during all steps, ATP binding was a prerequisite for biotin capture and ATP hydrolysis for subsequent biotin release.” makes more sense in this regard? Although the ATP/ATP NBD dimer is proposed to close after substrate release, the structures give no insight into how such signalling occurs, since the isolated ATPase dimer structures differ little from the apo full complex, and the apo full complex differs little from the ADP/ATP-bound full complex.

Response:

There are numerous ABC transporters that exhibit non-productive/futile ATPase activity. Again, the reviewer expects ECF-FolT2 and BioM2NY to behave in an identical way. We emphasize that they are very different proteins. In addition, we have shown previously in proteoliposomes that ATPase activity of ECF-FolT2 is not stimulated by folate (9), consistent with our model.

5. Conclusions.
Here the authors find a new conformation of an ATP-bound ABC ECF. In favour of this conformation being physiological, it provides an arguably plausible, if somewhat crude and indeed dubious mechanism by which ATP hydrolysis could eject the S subunit, as required by previous structures and the toppling mechanism. In view of the many reservations inconsistencies discussed above, and the

relatively elf-referential nature of the structural understanding of the enzyme mechanism, however, this is not sufficient to justify publication of this report in Nat Comm.
Indeed it might be said that this report represents a high water mark in the splintering of ABC transporter mechanisms into ever more weird, wonderful and ultimately improbable scenarios, dictated by the structural data. Notablty, all the while the soluble ATPase domains remain resolutely constant, not reflecting in anything even approaching proportion the putative structural variations observed in the TMDs, for which there remains no rationale, not even of the most conjectural kind: on this the field remains utterly mute.

Response:

We feel that the reviewer now resorts to dangerous non-scientific arguments. In our view, the highest water mark of weird and wonderful mechanisms is reflected in the diversity of life on earth. Weird, yes, wonderful, yes, improbable, perhaps, but evolution does not care about improbability. Selection for long enough allows for the improbable to happen. Where does the improbable happen? At the molecular level. So implicitly, the reviewer denies evolution here, which kills any constructive scientific discussion!

Reviewer #3 (Remarks to the Author):

In the manuscript "Expulsion mechanism of the substrate-translocating subunit in ECF transporters" Thangaratnarajah et al. utilized cryo-EM and single particle analysis to investigate multiple conformations of an ECF transporter. Their data and interpretation suggest a possible mechanism of this membrane protein.
The study is very well planned and executed, the data compelling, and this work's overall quality is also very good. In principle I have only minor comments that the authors will surely be able to address:

We thank the reviewer for taking his time to review our manuscript and for providing us with enthusiastic, constructive and positive feedback, and also pointing out a few minor errors, which we now have addressed.

Most of the structures were obtained in lipid nanodisc environment -which is totally fine. However, along the manuscript the authors raise several times major concerns about artefacts that were introduced by this environment (See lines 140, 123-124, 120). This is very concerning to read and only resoved in line 185 where a comparison between DDM and ND is presented. I highly recommend to sacrifcie this supsense for the sake of clarity and tone down the statements about the artifically induced conformations.

Response:

We thank the reviewer for this constructive comment. Indeed, we have been very open about our concerns that artefacts might occur, and tried to evaluate the risk of potentially artificially induced conformations experimentally by comparing structures in DDM and ND. We have now largely toned down the statement of artificially induced conformations in the manuscript, and introduced following changes as follows:

Page 5, line 124: "We argued that the lipid nanodisc assembly may act as a constraint, hindering conformational rearrangements required to expel the S-component. Similar restriction of conformational freedom by the nanodisc belt has been observed also for the ABC transporter MsbA[24]."

Page 5, line 124: "~~While ATP binding would induce the closure of the ATPase dimer and the expulsion of the S-component and hydrolysis, the lipid nanodisc constriction would counter the expulsion of the S-component and potentially the closure of the ATPase dimer and hydrolysis.~~"

Page 6, line 164: "~~To avoid the confinement of belt proteins,~~ We then tried to obtain samples for cryo-EM under turnover conditions with ECF-FolT2 in DDM micelles, instead of lipid nanodiscs."

Page 6, line 175: *"Next, we shifted our attention to the solitary ECF module of ECF-FolT2~~. to avoid hampered S-component expulsion in the nanodisc environment.,~~ which according to in vitro and in vitro studies must represent, at least transiently, an existing functional state in cells."*

Page 12, line 436: "We ~~attribute this discrepancy to a possibly the constricted nanodiscs environment that, by preventing the dissociation of the~~ hypothesize that the S-component, which remains associated with the ECF module in the nanodisc environment, hinders the conformational transition needed for ~~a full transition to a closed and convex state~~ hydrolysis of the second ATP molecule. This observation may hint at the requirement of S-component expulsion ~~before~~ associated with ATP hydrolysis at the second site."

Moreover, the comparison between the structure in lipid nanodiscs and DDM micelles refers to the solitary ECF module. To exclude any ambiguity, we have now clarified this in the text as follows:

Page 7, line 232: "The conformation of the solitary ECF module is virtually identical, but without the extra protein-resembling density, excluding a nanodisc-induced artefactual conformation."

It seems that Extended Extendend Fig2 is swapped - please correct or else correct all references in the text.
In line 116 the authors refer to (Extended Data I Fig. 1b) which I think is incorrect – instead they wanted to refere[21]nce Extended Da. Fig. 2b?
In line 128 and 129 they reference Extended Data Fig 1a – which would be a micrograph?

Lines 133 and 134... maybe at additional points as well...

Response:

Indeed, the references to the figures were not correct, which we have corrected and refer now to Extended Data Fig 3 a or b instead of Extended Data Fig 1 a or b where appropriate.

In line, 144 the authors state: "Unexpectedly, the protein complex rapidly aggregated, preventing structure determination. Nonetheless, this observation indicates that conformational changes take place under turnover conditions in detergent solution. The aggregation possibly occurred due to the expulsion of the S-component and the associated fission of the micelle leading to the exposure of hydrophobic surfaces to the aqueous environment."
While this could be an explanation for their observation this argument is only valid if it was the addition of ATP Mg2+, etc that caused the proteins to aggregate - no other potentially unkown effect. This can be quickly checked in negative stain with a before and after image of the same protein sample in detergent.

Response:

As already indicated in our response to reviewer #1, the ECF-FolT2 complex in DDM micelles visibly aggregated (turbid solution) when incubated with Mg-ATP prior to sample vitrification, but did not aggregate when incubated in absence of the nucleotide. We have now made it clear in the text as follows:

Page 6, line 151: "However, the protein complex rapidly aggregated <u>prior to sample vitrification</u>, hindering structure determination."

We have also observed a similar behaviour with Mg-AMP-PNP. No detectable trace was obtained when the sample was analysed by size-exclusion chromatography after aggregates were removed by high-speed centrifugation. We however did not observe any visible aggregation in the absence of Mg-ATP or Mg-AMP-PNP over a prolonged period of time, which still showed a single monodisperse peak after reinjection of a small concentrated sample stored 4 °C.

The authors discuss that the nanodisc environment hampers the repulsion of the S-component from the complex. I think this could be a valid explanation but I wonder why they then stick to this particular environment and do not choose another one for this experiment and thus prove this hypothesis.

Response:

Our attempts to investigate the expulsion of the S-component from the complex embedded within detergent micelles failed due to aggregation. We have also attempted to directly extract the full complex from the membranes using different SMA polymers, which was also unsuccessful. The ideal environment to provide a proof to our hypothesis would be to carry out structural studies in liposomes. While this is possible as demonstrated by the cryo-EM structures of AcrB (33), Eag Kv channel (34) and the ryanodine receptor (35), the relatively small sizes of the ECF module (~92 kDa) and the expelled S-component (~20 kDa) are currently prohibitive. Especially the detection of the S-component would be extremely difficult because of its small size and the lack of any extra-membranous features. Small protein binders such Fabs, nanobodies, sybodies etc. could enable structural efforts, which however requires the identification of such binders that do not affect protein function and merely function to increase the overall mass of the protein.

It would be helpful to label the S-component as such in Figure 1.

Response:

We have now amended in Fig 1a and labelled the S-component as such in the legend and indicated the identity of the S-component next to the protein as in Fig 1d. To be consistent, the label was also amended in Extended Data Fig 6 and Extended Data Fig 10, and in the former, the identity of the S-component is indicated next to the protein as in Fig 1a.

Also, it would help to introduce FolT2 as S-component earlier in the text (currently it is in line 154).

Response:

We have now introduced FolT2 as an S-component now earlier as follows:

Page 5, line 109: "The S-component <u>for folate (FolT2)</u> remains docked to the EcfT subunit in the substrate-free inward-facing toppled state (Fig. 1a)."

References

1. Karpowich, N. K., Song, J. M., Cocco, N., and Wang, D.-N. (2015) ATP binding drives substrate capture in an ECF transporter by a release-and-catch mechanism. *Nat Struct Mol Biol*. **22**, 565–571
2. Setyawati, I., Stanek, W. K., Majsnerowska, M., Swier, L. J. Y. M., Pardon, E., Steyaert, J., Guskov, A., and Slotboom, D. J. (2020) In vitro reconstitution of dynamically interacting integral membrane subunits of energy-coupling factor transporters. *eLife*. **9**, e64389
3. Henderson G. B., Zevely E. M., and Huennekens F. M. (1979) Mechanism of Folate Transport in Lactobacillus casei: Evidence for a Component Shared with the Thiamine and Biotin Transport Systems. *Journal of Bacteriology*. **137**, 1308–1314
4. Rodionov, D. A., Hebbeln, P., Eudes, A., ter Beek, J., Rodionova, I. A., Erkens, G. B., Slotboom, D. J., Gelfand, M. S., Osterman, A. L., Hanson, A. D., and Eitinger, T. (2009) A Novel Class of Modular Transporters for Vitamins in Prokaryotes. *JB*. **191**, 42–51
5. Eudes, A., Erkens, G. B., Slotboom, D. J., Rodionov, D. A., Naponelli, V., and Hanson, A. D. (2008) Identification of Genes Encoding the Folate- and Thiamine-Binding Membrane Proteins in Firmicutes. *Journal of Bacteriology*. **190**, 7591–7594
6. Xu, K., Zhang, M., Zhao, Q., Yu, F., Guo, H., Wang, C., He, F., Ding, J., and Zhang, P. (2013) Crystal structure of a folate energy-coupling factor transporter from Lactobacillus brevis. *Nature*. **497**, 268–271
7. Zhang, M., Bao, Z., Zhao, Q., Guo, H., Xu, K., Wang, C., and Zhang, P. (2014) Structure of a pantothenate transporter and implications for ECF module sharing and energy coupling of group II ECF transporters. *Proceedings of the National Academy of Sciences*. **111**, 18560–18565
8. Wang, T., Fu, G., Pan, X., Wu, J., Gong, X., Wang, J., and Shi, Y. (2013) Structure of a bacterial energy-coupling factor transporter. *Nature*. **497**, 272–276
9. Swier, L. J. Y. M., Guskov, A., and Slotboom, D. J. (2016) Structural insight in the toppling mechanism of an energy-coupling factor transporter. *Nat Commun*. **7**, 11072
10. Santos, J. A., Rempel, S., Mous, S. T., Pereira, C. T., ter Beek, J., de Gier, J.-W., Guskov, A., and Slotboom, D. J. (2018) Functional and structural characterization of an ECF-type ABC transporter for vitamin B12. *eLife*. **7**, e35828
11. Bao, Z., Qi, X., Hong, S., Xu, K., He, F., Zhang, M., Chen, J., Chao, D., Zhao, W., Li, D., Wang, J., and Zhang, P. (2017) Structure and mechanism of a group-I cobalt energy coupling factor transporter. *Cell Research*. **27**, 675–687
12. Karpowich, N. K., and Wang, D.-N. (2013) Assembly and mechanism of a group II ECF transporter. *Proceedings of the National Academy of Sciences*. **110**, 2534–2539
13. Pleiner, T., Tomaleri, G. P., Januszyk, K., Inglis, A. J., Hazu, M., and Voorhees, R. M. (2020) Structural basis for membrane insertion by the human ER membrane protein complex. *Science*. **369**, 433
14. Miller-Vedam, L. E., Bräuning, B., Popova, K. D., Schirle Oakdale, N. T., Bonnar, J. L., Prabu, J. R., Boydston, E. A., Sevillano, N., Shurtleff, M. J., Stroud, R. M., Craik, C. S., Schulman, B. A., Frost, A., and Weissman, J. S. (2020) Structural and mechanistic basis of the EMC-dependent biogenesis of distinct transmembrane clients. *eLife*. **9**, e62611
15. O'Donnell, J. P., Phillips, B. P., Yagita, Y., Juszkiewicz, S., Wagner, A., Malinverni, D., Keenan, R. J., Miller, E. A., and Hegde, R. S. (2020) The architecture of EMC reveals a path for membrane protein insertion. *eLife*. **9**, e57887
16. Bai, L., You, Q., Feng, X., Kovach, A., and Li, H. (2020) Structure of the ER membrane complex, a transmembrane-domain insertase. *Nature*. **584**, 475–478
17. Xu, D., Li, Y., Yang, F., Sun, C.-R., Pan, J., Wang, L., Chen, Z.-P., Fang, S.-C., Yao, X., Hou, W.-T., Zhou, C.-Z., and Chen, Y. (2022) Structure and transport mechanism of the human cholesterol transporter ABCG1. *Cell Reports*. **38**, 110298
18. Thangaratnarajah, C., Rheinberger, J., Paulino, C., and Slotboom, D. J. (2021) Insights into the bilayer-mediated toppling mechanism of a folate-specific ECF transporter by cryo-EM. *Proc Natl Acad Sci USA*. **118**, e2105014118
19. Hofmann, S., Januliene, D., Mehdipour, A. R., Thomas, C., Stefan, E., Brüchert, S., Kuhn, B. T., Geertsma, E. R., Hummer, G., Tampé, R., and Moeller, A. (2019) Conformation space of a heterodimeric ABC exporter under turnover conditions. *Nature*. **571**, 580–583
20. Galazzo, L., Meier, G., Januliene, D., Parey, K., De Vecchis, D., Striednig, B., Hilbi, H., Schäfer, L. V., Kuprov, I., Moeller, A., Bordignon, E., and Seeger, M. A. (2022) The ABC transporter MsbA adopts the wide inward-open conformation in *E. coli* cells. *Sci. Adv.* **8**, eabn6845
21. Oldham, M. L., Khare, D., Quiocho, F. A., Davidson, A. L., and Chen, J. (2007) Crystal structure of a catalytic intermediate of the maltose transporter. *Nature*. **450**, 515–521
22. Johnson, Z. L., and Chen, J. (2017) Structural Basis of Substrate Recognition by the Multidrug

Resistance Protein MRP1. *Cell*. [online] http://linkinghub.elsevier.com/retrieve/pii/S0092867417301319 (Accessed February 23, 2017)
23. Manolaridis, I., Jackson, S. M., Taylor, N. M. I., Kowal, J., Stahlberg, H., and Locher, K. P. (2018) Cryo-EM structures of a human ABCG2 mutant trapped in ATP-bound and substrate-bound states. *Nature*. [online] http://www.nature.com/articles/s41586-018-0680-3 (Accessed November 7, 2018)
24. Kieuvongngam, V., and Chen, J. (2022) Structures of the peptidase-containing ABC transporter PCAT1 under equilibrium and nonequilibrium conditions. *Proc. Natl. Acad. Sci. U.S.A.* **119**, e2120534119
25. Korkhov, V. M., Mireku, S. A., Veprintsev, D. B., and Locher, K. P. (2014) Structure of AMP-PNP–bound BtuCD and mechanism of ATP-powered vitamin B12 transport by BtuCD–F. *Nat Struct Mol Biol*. **21**, 1097–1099
26. Liu, F., Lee, J., and Chen, J. (2021) Molecular structures of the eukaryotic retinal importer ABCA4. *eLife*. **10**, e63524
27. Sikkema, H. R., van den Noort, M., Rheinberger, J., de Boer, M., Krepel, S. T., Schuurman-Wolters, G. K., Paulino, C., and Poolman, B. (2020) Gating by ionic strength and safety check by cyclic-di-AMP in the ABC transporter OpuA. *Sci Adv*. **6**, eabd7697
28. Olsen, J. A., Alam, A., Kowal, J., Stieger, B., and Locher, K. P. (2019) Structure of the human lipid exporter ABCB4 in a lipid environment. *Nat Struct Mol Biol*. 10.1038/s41594-019-0354-3
29. Chen, Z.-P., Xu, D., Wang, L., Mao, Y.-X., Li, Y., Cheng, M.-T., Zhou, C.-Z., Hou, W.-T., and Chen, Y. (2022) Structural basis of substrate recognition and translocation by human very long-chain fatty acid transporter ABCD1. *Nat Commun*. **13**, 3299
30. Xie, T., Zhang, Z., Yue, J., Fang, Q., and Gong, X. (2022) Cryo-EM structures of the human surfactant lipid transporter ABCA3. *Science Advances*. **8**, eabn3727
31. Jones, P. M., and George, A. M. (2020) Is the emperor wearing shorts? The published structures of ABC transporters. *FEBS Lett.* **594**, 3790–3798
32. Faustino, I., Abdizadeh, H., Souza, P. C. T., Jeucken, A., Stanek, W. K., Guskov, A., Slotboom, D. J., and Marrink, S. J. (2020) Membrane mediated toppling mechanism of the folate energy coupling factor transporter. *Nat Commun*. **11**, 1763
33. Yao, X., Fan, X., and Yan, N. (2020) Cryo-EM analysis of a membrane protein embedded in the liposome. *Proc Natl Acad Sci USA*. 10.1073/pnas.2009385117
34. Mandala, V. S., and MacKinnon, R. (2022) Voltage-sensor movements in the Eag Kv channel under an applied electric field. *Proc. Natl. Acad. Sci. U.S.A.* **119**, e2214151119
35. Melville, Z., Kim, K., Clarke, O. B., and Marks, A. R. (2022) High-resolution structure of the membrane-embedded skeletal muscle ryanodine receptor. *Structure*. **30**, 172-180.e3

REVIEWER COMMENTS

Reviewer #1 (Remarks to the Author):

The authors provide no further experimental evidences to convince me that the ECF module structure obtained is of biological significance. The structure differences observed in the ECF module with/without the ATP in the absence of S component are probably artifacts caused by binding lipids or detergents, especially for the different conformations of the coupling helices. The hypothetical model of the repulsion of S thus lacks necessary experimental support. I do not recommend for publication.

Reviewer #3 (Remarks to the Author):

The reviewers have sufficiently addressed all of my previous concerns. I suggest no further changes.

Reviewer #4 (Remarks to the Author):

The manuscript by Thangaratnarajah et al. reports several cryo-EM structures of solitary ECF in different conformational states and their follow-up coarse-grained molecular dynamics simulations to support a hypothetical transport mechanism for ECF transporters that involves a thermal ratchet mechanism and membrane morphology bidirectionally affecting the conformational state of protein. An important finding of this work seems to be the membrane-assisted conformational rearrangement of S-component from the substrate-free/nucleotide-bound to the substrate-bound/nucleotide-free state. Although this work is carefully done and it gives us some insight regarding the transport mechanism of ECF transporters, I am not convinced the insight provided is significant and novel.

Major points:

1. The cryo-EM structures are interesting. However, I am not convinced that they provide any additional evidence for the proposed mechanism. The authors have previously used the same techniques including cryo-EM and coarse-grained MD to support the same hypothesis (Nat. Comm. 11:1763, 2020 and PNAS 118:e2105014118, 2021). The new structures are certainly important but it is not clear to me how/if they have added much to what the authors have previously suggested.

2. An important part of the hypothesis provided is the membrane involvement. Reading the manuscript, the involvement of membrane morphology in the mechanism seems to be the focus of the manuscript or the most important finding (e.g., see abstratc). However, this is really the outcome of coarse-grained MD simulations and it is not directly related to the cry-EM structures. Given that the simulations are coarse-grained and not all-atom, I am not convinced that these findings are significant, given the fact that the same results were already observed in previous coarse-grained simulations (Nat. Comm. 11:1763, 2020).

Minor points:

1. The E. coli polar lipids used in nanodiscs and the composition used in the simulations are somewhat similar but not the same. For instance, I believe the nanodiscs used in experiments have about 10% CL but the simulations have 5% CL. Is there a reason for discrepancy? Sometimes such seemingly unimportant differences may turn out to be important.

2. Page 26, Line 808: The unit of bond force constant is not right (square angstrom is missing from the denominator).

Opinion on past Review:

I was asked by the editor to provide my opinion regarding the comments of Rev. #2 and authors' response to it. I don't have a strong opinion against the manuscript as the Rev. #2 implies but I agree with Rev. #2 that the authors are not taking other possibilities seriously. I think the authors should have responded to the comments carefully rather than dismissing them as I explain in more detail below.

On the issue of stoichiometry, I am siding more with the authors. While there is always alternative hypotheses and one may find new evidence to support one over another, the 1:1:1:1 stoichiometry is a reasonable assumption to make to interpret the data. The authors could of course have a discussion about it in the manuscript but I don't find this to be that controversial as highlighted by Rev. #1.

On the other issues, unfortunately, the authors have been very dismissive of the Rev. #1 review of the literature, arguing that other interpretations of the data are possible. As I explained in my own review, the authors interpret the new cryo-EM structures within the same framework they had already built based on prior structures and simulations. Rev. #1 has provided a great opportunity for the authors to discuss whether their new structures could be consistent with different hypothetical mechanisms or not.

For instance, is it possible to interpret these structures in a way that are consistent with a dynamic exchange transport mechanism?

Unfortunately, the authors have dismissed the comment by saying the mechanism has been suggested for group I ECF transporter and not group II ECF transporter. Similarly the toppling mechanism suggested by Rev. #2 is a sound mechanism. The authors argue against this mechanism but they could more elaborately discuss whether or not this mechanism is consistent with the new cryoEM data.

Reviewers comments:

Reviewer #1 (Remarks to the Author):

The authors provide no further experimental evidences to convince me that the ECF module structure obtained is of biological significance. The structure differences observed in the ECF module with/without the ATP in the absence of S component are probably artifacts caused by binding lipids or detergents, especially for the different conformations of the coupling helices. The hypothetical model of the repulsion of S thus lacks necessary experimental support. I do not recommend for publication.

Response:

We have now more extensively explained why we think that we are not studying an artefact.

Page 7, lines 325-330: Together, these experiments demonstrate that the solitary ECF module retains its integrity upon purification, and importantly can re-associate with a solitary S-component to form a functional transporter complex, excluding the possibility that the complex had collapsed in an artefactual, inactive state and reinforcing the notion that a solitary ECF module can exist in cells.

Page 7, lines 343-350: Notably, in the solitary ECF module, the docking site for the S-component remained structurally intact and unaltered compared to the docking site in the ECF-FolT2 complex. Yet, in contrast to the full complex, it is accessible to the lipid nanodisc environment (Extended Data Fig. 10a). Moreover, the docking site is hydrophobic (Extended Data Fig. 10b), and therefore compatible to interact with the surrounding environment of the lipid nanodisc. Such membrane-accessible interaction sites have been also observed in structures of the endoplasmic reticulum membrane protein complex (EMC)[28–32].

Reviewer #3 (Remarks to the Author):

The reviewers have sufficiently addressed all of my previous concerns. I suggest no further changes.

Response:

We thank the reviewer for taking the time to review our revised manuscript, and are pleased to hear that all of the previous concerns have been addressed to the reviewer's satisfaction.

Reviewer #4 (Remarks to the Author):

The manuscript by Thangaratnarajah et al. reports several cryo-EM structures of solitary ECF in different conformational states and their follow-up coarse-grained molecular dynamics simulations to support a hypothetical transport mechanism for ECF transporters that involves a thermal ratchet mechanism and membrane morphology bidirectionally affecting the conformational state of protein. An important finding of this work seems to be the membrane-assisted conformational rearrangement of S-component from the substrate-free/nucleotide-bound to the substrate-bound/nucleotide-free state. Although this work is carefully done and it gives us some insight regarding the transport mechanism of ECF transporters, I am not convinced the insight provided is significant and novel.

Response:

We thank the reviewer for taking the time to review our manuscript and providing us with constructive feedback, which we have addressed in detail below.

Major points:

1. The cryo-EM structures are interesting. However, I am not convinced that they provide any additional evidence for the proposed mechanism. The authors have previously used the same techniques including cryo-EM and coarse-grained MD to support the same hypothesis (Nat. Comm. 11:1763, 2020 and PNAS 118:e2105014118, 2021). The new structures are certainly important but it

is not clear to me how/if they have added much to what the authors have previously suggested.

Response:

The major new point is the structure of the ECF module in a closed ATP-bound conformation. This conformation is different from what had been proposed before (based on educated guesses) and shows for the first time the structural mechanism of coupling. We have now rewritten the abstract to make this point clearer.

In addition, the adaptation of the bilayer morphology depending on the conformational state of the transporter is new. In our previous work, we have only a single state, and single morphology i.e. with open ATPase dimers. Now we find that ATP binding and the closure of the ATPase dimer greatly affects the bilayer shape and thickness, and we interpret these changes in the light of the mechanism of transport. The interplay between bilayer morphology, conformational changes in the protein, substrate binding and mechanism of transport is new, even though it is consistent with previous speculation.

2. An important part of the hypothesis provided is the membrane involvement. Reading the manuscript, the involvement of membrane morphology in the mechanism seems to be the focus of the manuscript or the most important finding (e.g., see abstratc). However, this is really the outcome of coarse-grained MD simulations and it is not directly related to the cry-EM structures. Given that the simulations are coarse-grained and not all-atom, I am not convinced that these findings are significant, given the fact that the same results were already observed in previous coarse-grained simulations (Nat. Comm. 11:1763, 2020).

Response:

We apologize for not having been clear here. The major new point is the structure of the ECF module in a closed ATP-bound conformation, alongside structures of the open conformation in conditions that were not previously tested. The changes in membrane morphology that we present (including the MD simulations) are derived directly from the structures. The news value from our current simulations is the observation that membrane perturbations are conformationally specific, and are intricately linked with the mechanism of transport. We have tried to make the news value clearer by changing the abstract text and textual changes throughout the manuscript. In our previous work, we had shown, for a single open state, that the observed curvature and thinning is not an artefact of the CG model, by converting into atomistic representation using the backwards protocol (underlined):

Page 10, lines 469-471: These results are also consistent with previous atomistic simulations performed, which were performed with the ATPase open conformation only[20].

In addition, it is worth noting that the previous curvature and thinning results published in Faustino et al., 2020 (Nature Communications) were obtained with Martini 2 model, while now we performed simulations using Martini 3, the state of the art of this coarse-grained model.

Minor points:

1. The E. coli polar lipids used in nanodiscs and the composition used in the simulations are somewhat similar but not the same. For instance, I believe the nanodiscs used in experiments have about 10% CL but the simulations have 5% CL. Is there a reason for discrepancy? Sometimes such seemingly unimportant differences may turn out to be important.

Response:

We have shown before (Faustino et al., 2020 in Nature Communications) that CL is not required for transport of folate by ECF-FolT2. Therefore, we do not think that the difference in CL concentration between the experiments and the simulations is of great importance, but we agree that it would have been more elegant if we had used the same concentrations. The rational of using 5% was to keep fully consistent and comparable with the previous simulations performed in Faustino et al., 2020 (Nature Communications). In this way, the CG MD simulations performed with Martini 3 in our new study can be validated by the previous Martini 2 and atomistic simulations. This information is now included in the methods section describing the CG MD simulations (underlined)

Page 28, lines 1074-1076: This composition is consistent our previous simulation studies[20], allowing the validation of the new Martini 3 simulations performed here with the previous results obtained with atomistic simulations of the open conformation.

2. Page 26, Line 808: The unit of bond force constant is not right (square angstrom is missing from the denominator).

Response:

We thank the reviewer for noticing this typo. The proper unit here, as used in GROMACS md code is kJ mol^{-1} nm^{-2}.

Opinion on past Review:

I was asked by the editor to provide my opinion regarding the comments of Rev. #2 and authors' response to it. I don't have a strong opinion against the manuscript as the Rev. #2 implies but I agree with Rev. #2 that the authors are not taking other possibilities seriously. I think the authors should have responded to the comments carefully rather than dismissing them as I explain in more detail below.

Response:

We apologize for our seemingly dismissive response, which was triggered by some of the remarks of the reviewer, which we find biased and non-scientific. We do realize now that there were also a few fair points in the extremely extensive review document, and we have now incorporated a more balanced discussion in the manuscript.

On the issue of stoichiometry, I am siding more with the authors. While there is always alternative hypotheses and one may find new evidence to support one over another, the 1:1:1:1 stoichiometry is a reasonable assumption to make to interpret the data. The authors could of course have a discussion about it in the manuscript but I don't find this to be that controversial as highlighted by Rev. #1.

Response:

We agree with the reviewer that science is evolving and new findings may support alternative hypotheses. With respect to the subunit stoichiometry, we have now indicated the subunit stoichiometry in the introduction, but explicitly mention that deviating reports exist before structural data became available and has been discussed elsewhere as follows:

Page 3, lines 140-146: Structural and biophysical analyses of ECF transporters complexes from three different organisms, different substrate specificities, and from different laboratories revealed that the core complex and minimal functional unit exists in a 1:1:1:1 subunit stoichiometry[4–12]. Deviating subunit stoichiometries have been reported[13,14] before the first structural insights became available[8,9], which may indicate the existence of transient higher order complexes and have been previously discussed elsewhere[2].

On the other issues, unfortunately, the authors have been very dismissive of the Rev. #1 review of the literature, arguing that other interpretations of the data are possible. As I explained in my own review, the authors interpret the new cryo-EM structures within the same framework they had already built based on prior structures and simulations. Rev. #1 has provided a great opportunity for the authors to discuss whether their new structures could be consistent with different hypothetical mechanisms or not. For instance, is it possible to interpret these structures in a way that are consistent with a dynamic exchange transport mechanism?

Unfortunately, the authors have dismissed the comment by saying the mechanism has been suggested for group I ECF transporter and not group II ECF transporter.

Response:

We apologize once more for being somewhat dismissive on the few fair points that the reviewer raised. We now have included a more extensive discussion on the alternative mechanism.

Page 13, lines 534-542: Two models have been postulated for the process of substrate transport across the membrane (Fig. 4 – right half) – namely the power stroke and thermal ratchet mechanism[2]. In the power stroke mechanism, it was proposed that a substrate-loaded S-component binds to the ECF module in the nucleotide-bound and closed conformation[2,12]; followed by ATP hydrolysis and opening of the ATPase dimer to power the toppling of the S-component. Instead, in the thermal ratchet mechanism, ATP hydrolysis and opening of the ATPase dimer occurs prior to the binding of a substrate-loaded S-component, and acts as a resetting step to restore the docking site for the S-component. Subsequently, toppling occurs sponteneously[2,4].

Page 13, lines 592-595: *It is noteworthy that the alternative power-stroke mechanism was proposed for the group I biotin ECF transporter BioMNY with a dedicated ECF module for a single S-component[38,39], which may indicate that there is mechanistic diversity among ECF transporters.*

Page 14, lines 646-649: Similarly, in group I transporters such as BioMNY, the S-component may not be separated from the ECF module to a great extent [38,39], explaining differences in apparent complex stability found in in vitro experiments compared to group II transporters.

Similarly the toppling mechanism suggested by Rev. #2 is a sound mechanism. The authors argue against this mechanism but they could more elaborately discuss whether or not this mechanism is consistent with the new cryoEM data.

Response:

We in fact do not argue against the toppling mechanism, and apologize for the misunderstanding. On the contrary, the toppling mechanism is part of the transport cycle and is the foundation of our work! As elaborated in more detail in the discussion section, our new cryo-EM structures provide insights into how the S-component rotates back from the toppled inward-facing (Figure 4 – state 1) to the outward-facing state (Figure 4 – state 2 and 3) upon ATP binding and ATP hydrolysis – a necessary step to complete the transport cycle. Following these steps, the S-component transitions from an outward-facing substrate-bound (Figure 4 – state 4) to a toppled inward-facing substrate-released state (Figure 4 – state 1). We have now labelled the transition from state 4 to state 1 in Figure 4 with “Toppling” to emphasize that the toppling mechanism is part of our proposed transport cycle for group II ECF transporters.
